# The association between white matter tract structural connectivity and information processing speed in relapsing-remitting multiple sclerosis

**DOI:** 10.1007/s10072-023-06817-6

**Published:** 2023-04-27

**Authors:** Magdalena Chylińska, Bartosz Karaszewski, Jakub Komendziński, Adam Wyszomirski, Marek Hałas, Edyta Szurowska, Agnieszka Sabisz

**Affiliations:** 1 Department of Adult Neurology, Faculty of Medicine, Medical University of Gdańsk, Dębinki 7, 80-211 Gdańsk, Poland; 2 Second Department of Radiology, Faculty of Medicine, Medical University of Gdańsk, Gdańsk, Poland

**Keywords:** Multiple sclerosis (MS), Information processing speed (IPS), MRI, Diffusion-weighted (DW), Diffusion tensor imaging (DTI), Tractography, Cognition, TRACULA (TRActs Constrained by UnderLying Anatomy)

## Abstract

**Background:**

Information processing speed (IPS) deterioration is common in relapsing-remitting multiple sclerosis (RRMS) patients [1] and might severely affect quality of life and occupational activity. However, understanding of its neural substrate is not fully elucidated. We aimed to investigate the associations between MRI-derived metrics of neuroanatomical structures, including the tracts, and IPS.

**Methods:**

Symbol Digit Modalities Test (SDMT), Paced Auditory Serial Addition Test (PASAT), and Color Trails Test (CTT) were used to evaluate IPS in 73 RRMS consecutive patients, all undergoing only interferon beta (IFN-*β*) therapy during the study. At the same time, 1.5T MRI including diffusion tensor imaging (DTI) data was acquired for each recruited subject. We analyzed volumetric and diffusion MRI measures (FreeSurfer 6.0) including normalized brain volume (NBV), cortical thickness (thk), white matter hypointensities (WMH), volume (vol), diffusion parameters: mean (MD), radial (RD), axial (AD) diffusivities, and fractional anisotropy (FA) of 18 major white-matter (WM) tracts. Multiple linear regression model with interaction resulted in distinguishing the neural substrate of IPS deficit in the IPS impaired subgroup of patients.

**Results:**

The most significant tract abnormalities contributing to IPS deficit were right inferior longitudinal fasciculus (R ILF) FA, forceps major (FMAJ) FA, forceps minor (FMIN) FA, R uncinate fasciculus (UNC) AD, R corticospinal tract (CST) FA, and left superior longitudinal fasciculus FA (L SLFT). Among volumetric MRI metrics, IPS deficit was associated with L and R thalamic vol. and cortical thickness of insular regions.

**Conclusion:**

In this study, we showed that disconnection of the selected WM tracts, in addition to cortical and deep gray matter (GM) atrophy, might underlie IPS deficit in RRMS patients but more extensive studies are needed for precise associations.

**Supplementary Information:**

The online version contains supplementary material available at 10.1007/s10072-023-06817-6.

## Introduction

Cognitive deficit is widespread in multiple sclerosis (MS), affecting from 50 to 73% of patients [1]. The mostly affected cognitive domains include attention and processing speed, executive functions, immediate and delayed recall or memory, and verbal fluency [1]. The slowing of information processing speed was firstly described by Charcot in 1877 [2] and remains the most prevalent cognitive deficit in multiple sclerosis patients [3]. In a large sample of RRMS, IPS impairment varied by the test used from 27 to 51% [4]. Impaired IPS affects MS patients’ quality of life including their educational and occupational attainment [5, 6]. IPS is usually measured as the amount of information processed in a unit of time or the time needed to process a given amount of information [1]. There are well-established methods of assessment of IPS: Symbol Digit Modalities Test (SDMT) [7], Paced Auditory Serial Addition Test (PASAT) [8], Color Trails Test (CTT) [9], out of which SDMT is one of the most reliable and sensitive, and is strongly associated with some MRI characteristics such as T2 lesion volume and global brain atrophy [10, 11]. Many studies have been performed to identify the biological characteristics associated with CI in MS. One possible mechanism is so-called disconnection syndrome, where — in simplified terms — extensive WM damage affects network collapse between brain regions [12].

MR diffusion tensor imaging (DTI) might provide some information about the microstructural integrity of WM tracts because water diffusion in WM has a preferential orientation, which is restricted by fiber tracts [13]. The DTI metrics most frequently used to assess the WM integrity include FA for the measurement of diffusion directionality, and MD, which is independent of the spatial orientation of tissue structures [14]. Additional DTI metrics are AD and RD, which can offer information such as axonal damage and demyelination [15, 16]. In a post-mortem DTI MR study, MD and FA were associated with myelin content and to a lesser degree axonal count [17]. Decreased FA and increased MD in certain WM tracts were shown to be related with selected neuropsychological symptoms in RRMS patients such as processing speed [18, 19].

Although there are studies in the literature on IPS in RRMS, studies on multimodal analyses of many neuroimaging parameters using TRACULA and brain volumetrics assessing the basis of IPS deficit in RRMS are limited. Literature on the associations between WM tract connectivity and cognitive impairment in IPS appearing in some patients with RRMS is present, with brain atrophy, particularly GM atrophy, among the primarily considered and studied factors in this context [20, 21]. Indeed, previous studies identified some specific patterns of brain atrophy in cognitively impaired MS patients that specifically assume atrophy of the posterior cingulate cortex and bilaterally temporal poles [22]. Processing speed performances specifically have been found to relate to deep GM structure volumes, mainly of the thalamus and putamen [5, 10, 23].

In line with this view, the goal of our study is the assessment of the complex structural underpinnings of IPS deterioration using multimodal MRI evaluation, thus including both grey and white matter, the latter with tractography, in the same sample of RRMS patients on homogenous, IFN-*β* therapy. The analysis of imaging data was performed using the FreeSurfer software for volumetry of whole brain, thalamus, and WMH. Diffusion MRI parameters were used to examine tract-specific contributors of IPS deficit. Specifically, we applied TRACULA, an automated global probabilistic tractography tool with anatomical priors [24], which reconstructs a set of major white-matter pathways by incorporating prior information on the structural segmentation labels that each pathway goes through or is next to, as a function of the position along the length of the pathway. The assessment of IPS was performed using well-established SDMT, PASAT-3, and CTT*. Finally*, we performed an analysis of the correlation between the imaging measures and neuropsychological data. In the clinical dataset, we analyzed SDMT, PASAT, and CTT together with age, gender, and disease duration also in the multivariate regression model. To the best of our knowledge, this is the first study implementing such a multimodal MRI assessment, including FreeSurfer and TRACULA software analyses, to identify structural markers of IPS deficit in an RRMS population that, importantly, remains on homogenous disease modifying therapy (DMT).

## Materials and methods

### Population

Seventy-three patients of the Dept. of Adult Neurology, at the University Clinical Center in Gdańsk (the main hospital of the Medical University of Gdańsk), Poland, with diagnosed RRMS according to the 2010 McDonald Criteria [25], were included in the study prospectively between December 2015 and December 2019.

Inclusion criteria: Polish-speaking patients, with diagnosed RRMS, treated with IFN-β, EDSS score 0–5.5. Exclusion criteria: patients with disorders that might significantly influence neuropsychological testing (i.e., hypo- or hyperthyroidism, depression) or that might be responsible for similar neuroimaging changes (i.e., diagnosed vascular pathology, arterial hypertension, neuroborreliosis), history of traumatic brain injury, contraindications to the MRI study, disability of an arm interfering with writing, an intake of psychoactive drugs, diagnosis of psychiatric conditions, and insufficient quality of the MRI study.

All participants had a negative history of relapse and corticosteroid use during 30 days before MRI acquisition. The demographic and clinical characteristics of the RRMS population are provided in Table 1 and Supplement 1.

**Fig. 1:**
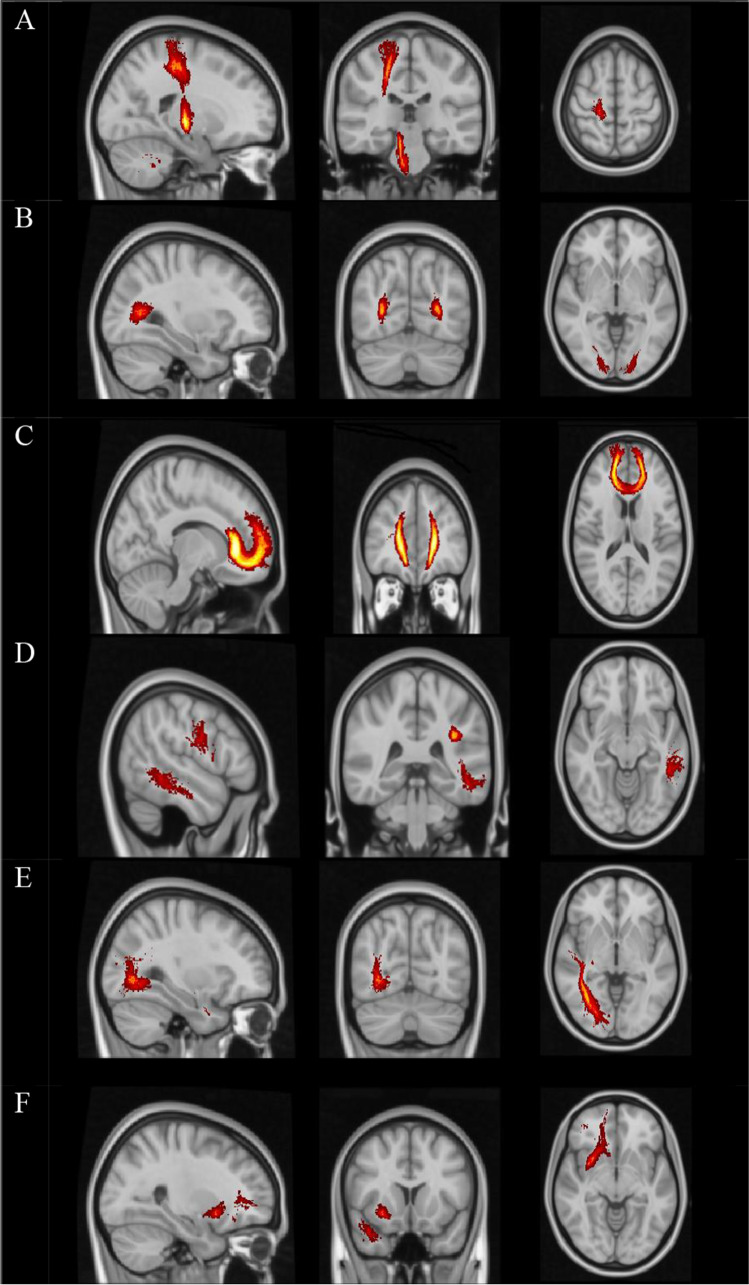
WM tracts correlated with IPS deficit were automatically visualized by use of the FSLeyes package. Presented tracts: **A** R CST, **B** FMAJ, **C** FMIN, **D** L SLFT, **E** R ILF, and **F** R UNC from JHU White-Matter Tractography Atlas were overlaid on standard head T1 images

**Table 1:** Demographic, clinical, and MRI characteristics of IPS preserved and IPS impaired subgroups of RRMS patients

**Clinical measures**	IPS preserved **(*N*=57)**	IPS impaired **(*N*=16)**	***p*-value**
Age [years]			0.405
Mean (SD)	37.84 (9.61)	40.0 (6.96)	
Median (Q1,Q3)	39.0 (29.0, 45.0)	44.0 (34.0, 45.0)	
Female sex			0.696
*N* (%)	42 (73.7)	11 (68.8)	
Disease duration [years]			0.136
Mean (SD)	8.14 (4.194)	10.32 (7.552)	
Median (Q1,Q3)	9.0 (5.0, 10.0)	9.5 (4.000, 14.250)	
Education [years]			0.179
Mean (SD)	15.18 (2.346)	14.25 (2.646)	
Median (Q1,Q3)	16.0 (13.0, 17.0)	13.5 (12.0, 17.0)	
EDSS			**0.011**
Mean (SD)	**2.333 (0.883)**	**3.000 (0.949)**	
Median (Q1,Q3)	**2.500 (1.500, 2.500)**	**3.000 (2.375, 3.500)**	
SDMT			**< 0.001**
Mean (SD)	**48.737 (9.83)**	**34.562 (11.87)**	
Median (Q1,Q3)	**49.0 (44.0, 54.0)**	**34.50 (25.75, 46.25)**	
PASAT			**< 0.001**
Mean (SD)	**47.054 (8.158)**	**35.40 (13.426)**	
Median (Q1,Q3)	**48.0 (41.75, 54.0)**	**33.0 (27.50, 47.50)**	
CTT 1 time			**< 0.001**
Mean (SD)	**35.684 (11.466)**	**57.250 (20.358)**	
Median (Q1,Q3)	**34.0 (27.0, 41.0)**	**55.000 (44.0, 65.25)**	
CTT 2 time			**< 0.001**
Mean (SD)	**74.930 (16.736)**	**114.938 (28.899)**	
Median (Q1,Q3)	**76.0 (61.0, 85.0)**	**113.000 (98.0, 132.75)**	
**MRI measures**			
L thalamus vol. ×10^−4^			**0.013**
Mean (SD)	**46.203 (4.575)**	**42.527 (6.791)**	
Median (Q1,Q3)	**45.425 (44.185, 48.413)**	**45.665 (36.793, 48.567)**	
R thalamus vol. ×10^−4^			**< 0.001**
Mean (SD)	**43.147 (4.465)**	**38.455 (5.587)**	
Median (Q1,Q3)	**43.218 (41.424, 45.329)**	**38.083 (32.907, 43.159)**	
WMH vol. ×10^−4^			**0.004**
Mean (SD)	**27.754 (18.755)**	**50.121 (45.011)**	
Median (Q1,Q3)	**20.015 (13.760, 36.804)**	**32.830 (20.834, 61.080)**	
NBV ×10^−2^			0.058
Mean (SD)	73.737 (4.129)	71.374 (5.002)	
Median (Q1,Q3)	74.269 (71.501, 75.965)	73.378 (67.091, 75.712)	
FMAJ FA ×10^−1^			0.066
Mean (SD)	5.33 (0.39)	5.09 (0.57)	
Median (Q1,Q3)	5.37 (5.11, 5.60)	5.19 (4.92, 5.42)	
FMIN FA × 10^−1^			0.981
Mean (SD)	4.65 (0.41)	4.65 (0.30)	
Median (Q1,Q3)	4.65 (4.34, 0.493)	4.63 (4.45, 4.82)	
R ILF FA ×10^−2^			0.127
Mean (SD)	39.271 (3.924)	37.577 (3.684)	
Median (Q1,Q3)	39.605 (36.977, 42.202)	37.959 (34.431, 40.543)	
R UNC AD ×10^−5^[mm^2^/s]			**0.028**
Mean (SD)	**110.176 (5.247)**	**113.435 (4.674)**	
Median (Q1,Q3)	**110.777 (107.866, 113.615)**	**114.145 (110.211, 115.688)**	
R CST FA × 10^−1^			0.254
Mean (SD)	4.666 (0.224)	4.743 (0.274)	
Median (Q1,Q3)	4.675 (4.549, 4.779)	4.726 (4.523, 4.968)	
SLFT FA ×10^−1^			0.911
Mean (SD)	4.01 (0.29)	4.02 (0.28)	
Median (Q1,Q3)	3.98 (3.83, 4.22)	4.06 (3.87, 4.20)	
L insula cortical thk. [mm]			0.666
Mean (SD)	2.617 (0.177)	2.596 (0.166)	
Median (Q1,Q3)	2.626 (2.498, 2.732)	2.591 (2.463, 2.672)	

*Abbreviations*: *AD*, axial diffusivity; *CC*, corpus callosum; *CST*, corticospinal tract; *CTT*, Color Trails Test; *EDSS*, Expanded Disability Status Score; *FA*, fractional anisotropy; *FMAJ*, corpus callosum–forceps major; *ILF*, inferior longitudinal fasciculus; *IPS*, information processing speed; *L*, left hemisphere; *mm*, millimeters; *NBV*, normalized brain volume; *PASAT*, Paced Auditory Serial Additive Test; *R*, right hemisphere; *s*, second; *SDMT*, Symbol Digit Modalities Test; *thk*, thickness; *UNC*, uncinate fasciculus; *vol*, volume normalized to estimated total intracranial volume; *WMH*, white matter hypointensities

Bolded entries represents values the p value reached significance (*p*<0.05)

### Neurological tests

An expanded neurological assessment, preceded by obtaining of demographic and medical history, was performed for each patient by an experienced neurologist (MC) using the Expanded Disability Status Score (EDSS) [26] at the time of his/her recruitment into the study. The patients did not have any physical disabilities which potentially might interfere with the execution of the neuropsychological testing.

### Neuropsychological assessments

Blind to all clinical and MRI data, an experienced neuropsychologist performed a neuropsychological assessment of all the enrolled patients. All the enrolled patients underwent the following tests:

Symbol Digit Modalities Test (SDMT) — a written version was performed according to the manual instructions [27].

Color Trails Test (CTT) — a new version of the Trail Making Test-TMT (Trail Making Test-TMT); the CTT-1 and 2 times were measured [28].

Paced Auditory Serial Additive Test (PASAT) — a measure of auditory information processing and attention: the task involves the consecutive adding of 60 pairs of digits presented in a series with 3-second intervals (each digit is added to the preceding one); the number of correct responses [29].

Also, Beck Depression Inventory-II (BDI-II) [30] — the Polish version of the questionnaire [31], was performed. In BDI-II, we found 60 patients with minimal (0–13), 11 patients with mild [13-17, 32], and 2 patients with moderate [18-26] depressive symptoms. Patients with more than 28 points in BDI-II were excluded from the study [33].

In the study population, two groups, IPS impaired and IPS preserved, were distinguished. Patients were classified as IPS impaired when the results of at least two cognitive tests were abnormal. The CTT [34] test results were considered abnormal when they were below the Polish limit for a given age and education; SDMT [35] results below 1SD were acknowledged as abnormal; PASAT-3 z-score results below −1 were considered abnormal [29].

### MRI data acquisition

The patients underwent an MRI on the same day as the clinical examinations. The MRI was performed with a 1.5T scanner (Siemens Magnetom Aera) using a 20-channel head/neck coil. The MRI protocol included the standard brain protocol for MS patients (sequences: T1-weighted 3D, FLAIR 3D, DIR 3D, SWI, DWI, T2-weighted sagittal and axial orientation, T1-weighted 3D — 10 min after the injection of the contrast medium) and DTI. The analysis in this study was performed with the T1-weighted 3D MPRAGE sequence and DTI; the parameters were as follows: T1-weighted MPRAGE sequence (transverse orientation, *TR*=1800 ms, *TE*=3.3 ms, *TI*=1000 ms, voxel size 1.4 mm × 1.4 mm × 1.1 mm, FOV 270 mm × 270 mm, 144 slices, *NSA*=1) and DTI (axial plane without angulation, *TR*=6500 ms, *TE*=85 ms, matrix 114 × 114, FOV 260 mm × 260 mm, 50 slices, slice thickness 3 mm, 3 images of *b*=0 s/mm2 and 3 images of *b*=1000 s/mm2 with 30 diffusion gradient directions).

### MRI data analysis

T1-weighted images and DTI images were converted to NII format by MRIConvert (https://lcni.uoregon.edu/downloads/mriconvert). Volumes of brain structures and cortical thickness were measured by the freely available software FreeSurfer, version 6.0 (http://surfer.nmr.mgh.harvard.edu) [36, 37]. The standard FreeSurfer processing stream recon-all was used. Data were visually inspected. The volumes obtained from analyses were normalized to estimate the total intracranial volume. The T1-weighted processing includes the motion correction and averaging (Reuter et al., 2010) [38] of multiple volumetric T1-weighted images, the removal of non-brain tissue, automated Talairach transformation, the segmentation of subcortical white matter and deep gray matter volumetric structures (Fischl et al., 2002; Fischl et al., 2004a) [39, 40], intensity normalization (Sled et al., 1998) [41], automated topology correction (Fischl et al., 2001; Segonne et al., 2007) [42, 43], and surface deformation.

The quality of diffusion tensor images was checked using the in-house procedure. The number of outliers in the model and motion was inspected with Explore DTI software. Patients whose head rotation and movement were more than 1° and 1 mm were excluded from the analysis. Diffusion images were pre-processed by TRACULA FreeSurfer trac-all script [24]. The pre-processing of the diffusion-weighted images consisted of the correction of image distortions due to eddy currents and B0 field inhomogeneities. A probabilistic tractography of 18 major white-matter pathways was performed. The measures of each tract included:

1. Number of sample paths in the WM tract (count),
2. Tract volume (in voxels),
3. Maximum, minimum, and average length of sample paths (in voxels),
4. Length of the highest-probability (a.k.a. maximum a posteriori) path,
5. Axial diffusivity (average over the entire support of the path distribution, weighted average over the entire support of the path distribution, and average over the highest-probability path only),
6. Radial diffusivity (as above),
7. Mean diffusivity (as above),
8. Fractional anisotropy (as above).

The DTI parameters were measured in the below-listed WM tracts, including projection, association, and commissural fibers:

Corticospinal tract (CST)

Anterior thalamic radiation (ATR)

Superior longitudinal fasciculus–parietal bundle (SLFP)

Superior longitudinal fasciculus–temporal bundle (SLFT)

Inferior longitudinal fasciculus (ILF)

Cingulum–cingulate gyrus (supracallosal) bundle (CCG)

Cingulum–angular (infracallosal) bundle (CAB)

Corpus callosum–forceps major (FMAJ)

Corpus callosum–forceps minor (FMIN)

Uncinate fasciculus (UNC)

### Statistical analysis

Clinical and MRI measures are summarized in Table 1 and Supplement 1. Continuous data were presented as mean, standard deviation, and quartiles. The sex variable was reported as count and percentage. We applied the Mann–Whitney *U* test for a comparison of clinical and MRI measures between the two subgroups of patients — IPS preserved and IPS impaired, whereas the chi-squared test was used to compare binomial data. The SDTM, PASAT z-cognitive, CTT-1, and CTT-2 outcomes were analyzed using a univariate linear regression model with MRI measures as independent variables in the two subgroups (IPS preserved and IPS impaired) of patients. A multiple linear regression model was used for clinical outcomes (PASAT z-cognitive, SDMT, CTT-1, CTT-2). This analysis was adjusted for the MRI measures and subgroups (IPS preserved and IPS impaired) and the interaction between them. The results of the regression models are expressed as an estimate (beta coefficient), 95% confidence interval, *p*-value, and coefficient of determination (R-squared). The coefficient of determination (R-squared) was used to assess the goodness-of-fit of the statistical model. Multiple regression models included two types of predictors: [1] variables of interest — MRI measures, subgroups (IPS preserved and IPS impaired) and interaction between them; [2] confounders — age, sex, education, and EDSS. A *p*-value of less than 0.05 was considered to be statistically significant. All statistical analyses were performed using the R statistical package (version 3.6.3; https://www.r-project.org/).

## Results

### Demographic and clinical characteristics

We recruited 80 patients with RRMS diagnosed according to the 2010 McDonald Criteria [25]. Four subjects were excluded due to motion artifacts in the DTI MRI studies; another three subjects were excluded due to severe depressive symptoms in BDI-II. Finally, 73 patients with complete clinical and radiological data entered the analysis.

In the studied cohort, 16 (21.91%) patients were recognized as IPS impaired and 57 (78.08%) patients were IPS preserved. There were no statistically significant differences in age, disease duration, and education duration between the subgroups. We observed significant lower scores in all cognitive tests in the IPS impaired subpopulation. According to the volumetric MR parameters, the IPS impaired subgroup had significantly lower R and L thalamus vol. and greater WMH vol. We also found lower cortical thk. of L pars opercularis and greater R UNC AD in the IPS impaired subgroup. The demographic and clinical characteristics, and MRI metrics of patients are provided in Table 1 and Supplement 1.

### Relation between MRI metrics and IPS

In the RRMS cohort, we found a significant interaction between the IPS preserved and IPS impaired subgroups and MRI measures, indicating that the relationship between MRI metrics and cognitive outcome differed depending on the subgroup.

We indicated DTI metrics of WM tracts most accurately, explaining the results obtained in specified cognitive tests (Table 2, more detailed data presented in Supplement 3). We observed that the CTT-1 results in the subgroup were associated with SLFT FA (*p*=0.019). The CI subgroup CTT-2 results were correlated with R ILF FA (*p*=0.02), R UNC AD (*p*=0.015), FMAJ (*p*=0.36), and FMIN FA (*p*=0.008). We found that the SDMT results were R ILF AD (*p*=0.009), R UNC AD (*p*=0.015), and R CST FA (*p*=0.028). Interestingly, there were no tracts relevant to the PASAT score.

**Table 2:** Multiple regression models with DTI MRI measures, IPS preserved and IPS impaired subgroups of RRMS patients, and interaction between them for cognitive tests. Data adjusted for age, sex, education, and EDSS

	**Estimate (beta coefficient)**	**Lower 95% CI**	**Upper 95% CI**	***p*-value**	***R***^**2**^
**CTT-1**					
**L SLFT FA ×10**^**−2**^	−0.191	−1.545	1.162	0.779	**0.373**
Subgroup IPS impaired (Ref. IPS preserved)	**−106.328**	**−212.5**	**−0.144**	**0.050**	
Interaction (SLFPFA×10^**−**2^, Subgroup)	**3.180**	**0.533**	**5.827**	**0.019**	
Age	0.151	−0.215	0.517	0.412	
Sex (Ref. female)	6.192	−1.010	13.395	0.091	
Education (years)	0.642	−0.791	2.076	0.374	
EDSS	1.919	−1.754	5.591	0.301	
**CTT-2**					
**R ILF FA ×10**^**−2**^	0.029	−1.169	1.228	0.961	**0.552**
Subgroup IPS impaired (Ref. IPS preserved)	**201.126**	**98.089**	**304.164**	**<0.001**	
Interaction (RILFFA×10^**−**2^, subgroup)	**−4.378**	**−7.083**	**−1.673**	**0.002**	
Age	0.337	−0.137	0.811	0.160	
Sex (Ref. female)	9.696	0.535	18.857	0.038	
Education (years)	1.077	−0.696	2.850	0.230	
EDSS	**6.315**	**1.381**	**11.248**	**0.013**	
**R UNC AD ×10**^**−5**^[mm^2^/s]	0.054	−0.869	0.977	0.908	**0.524**
Subgroup IPS impaired (Ref. IPS preserved)	**−287.102**	**−543.5**	**−30.738**	**0.029**	
Interaction (RUNCAD×10^**−**5^[mm^2^/s], subgroup)	**2.853**	**0.580**	**5.125**	**0.015**	
Age	0.395	−0.092	0.881	0.110	
Sex (Ref. female)	**11.641**	**1.920**	**21.361**	**0.020**	
Education (years)	1.232	−0.582	3.047	0.180	
EDSS	**6.015**	**0.881**	**11.150**	**0.022**	
**FMIN FA ×10**^**−2**^	0.157	−1.116	1.430	0.806	**0.523**
Subgroup IPS impaired (Ref. IPS preserved)	**235.649**	**89.924**	**381.374**	**0.002**	
Interaction(FMINFA×10^**−**2^, subgroup)	**−4.331**	**−7.486**	**−1.176**	**0.008**	
Age	0.329	−0.162	0.820	0.186	
Sex (Ref. female)	**10.864**	**0.895**	**20.834**	**0.033**	
Education (years)	0.820	−1.079	2.719	0.392	
EDSS	**7.265**	**2.219**	**12.311**	**0.005**	
**FMAJ FA ×10**^**−2**^	0.049	−1.246	1.344	0.940	**0.518**
Subgroup IPS impaired (Ref. IPS preserved)	**146.881**	**40.833**	**252.929**	**0.007**	
Interaction (FMAJFA×10^**−**2^, subgroup)	**−2.187**	**−4.226**	**−0.149**	**0.036**	
Age	0.337	−0.152	0.826	0.174	
Sex (Ref. female)	8.823	−0.848	18.494	0.073	
Education (years)	1.083	−0.780	2.946	0.250	
EDSS	**6.778**	**1.692**	**11.865**	**0.010**	
**SDMT**					
**R ILF AD ×10**^**−5**^**[mm**^**2**^**/s]**	0.228	−0.184	0.639	0.274	**0.439**
Subgroup IPS impaired (Ref. IPS preserved)	**133.466**	**25.631**	**241.301**	**0.016**	
Interaction (RILFAD ×10^**−**5^[mm^2^/s], subgroup)	**−1.212**	**−2.117**	**−0.306**	**0.009**	
Age	−0.055	−0.295	0.186	0.652	
Sex (Ref. female)	**−5.599**	**−10.32**	**−0.881**	**0.021**	
Education (years)	0.884	−0.031	1.799	0.058	
EDSS	**−3.849**	**−6.408**	**−1.291**	**0.004**	
**R UNC AD ×10**^**−5**^**[mm**^**2**^**/s]**	−0.340	−0.781	0.102	0.129	**0.477**
Subgroup IPS impaired (Ref. IPS preserved)	**124.929**	**2.317**	**247.541**	**0.046**	
Interaction(RUNCAD×10^**−**5^[mm^2^/s], subgroup)	**−1.187**	**−2.273**	**−0.100**	**0.033**	
Age	−0.031	−0.263	0.202	0.794	
Sex (Ref. female)	**−6.094**	**−10.74**	**−1.444**	**0.011**	
Education (years)	**1.111**	**0.243**	**1.979**	**0.013**	
EDSS	**−3.925**	**−6.381**	**−1.470**	**0.002**	
**R CST FA ×10**^**−1**^	6.733	−4.071	17.537	0.218	**0.422**
Subgroup IPS impaired (Ref. IPS preserved)	**98.257**	**1.466**	**195.048**	**0.047**	
Interaction (RCSTFA×10^**−**1^, subgroup)	**−23.105**	**−43.59**	**−2.616**	**0.028**	
Age	−0.065	−0.310	0.179	0.596	
Sex (Ref. female)	−4.275	−9.038	0.487	0.078	
Education	**1.170**	**0.253**	**2.087**	**0.013**	
EDSS	**−3.790**	**−6.352**	**−1.227**	**0.004**	

*Abbreviations*: *AD*, axial diffusivity; *CI*, confidence interval; *CST*, corticospinal tract; *CTT*, Color Trails Test; *e-TIV*, estimated total intracranial volume; *FA*, fractional anisotropy; *FMAJ*, corpus callosum–forceps major; *FMIN*, corpus callosum–forceps minor; *ILF*, inferior longitudinal fasciculus; *IPS*, information processing speed; *L*, left hemisphere; *R*, right hemisphere; *R*^2^, coefficient of determination; *s*, second; *SDMT*, Symbol Digit Modalities Test; *SLFT*, superior longitudinal fasciculus–temporal bundle; *UNC*, uncinate fasciculus

Bolded entries represents values the p value reached significance (*p*<0.05)

**Table 3:** Multiple regression models with volumetric MRI measures, IPS preserved and IPS impaired subgroups of RRMS patients, and interaction between them for cognitive tests. Data adjusted for age, sex, education, and EDSS

	**Estimate (beta coefficient)**	**Lower 95% CI**	**Upper 95% CI**	***p*-value**	***R***^**2**^
**CTT-2**					
**L thalamus vol. ×10**^**4**^	−0.922	−1.927	0.083	0.072	**0.594**
Subgroup IPS impaired (Ref. IPS preserved)	**106.583**	**32.617**	**180.549**	**0.005**	
Interaction (L thalamus vol. ×10^4^, subgroup)	**−1.708**	**−3.368**	**−0.049**	**0.044**	
Age	0.405	−0.050	0.861	0.080	
Sex (Ref. female)	7.085	−1.903	16.074	0.120	
Education	1.377	−0.301	3.055	0.106	
EDSS	**5.343**	**0.561**	**10.125**	**0.029**	
**R thalamus vol. ×10**^**3**^	−3.045	−12.965	6.875	0.542	**0.606**
Subgroup IPS impaired (Ref. IPS preserved)	**166.811**	**91.381**	**242.241**	**< 0.001**	
Interaction (R thalamus vol. ×10^3^, subgroup)	**−34.072**	**−52.761**	**−15.383**	**< 0.001**	
Age	0.464	0.022	0.907	0.040	
Sex (Ref. female)	**10.698**	**1.878**	**19.518**	**0.018**	
Education (years)	1.104	−0.548	2.756	0.187	
EDSS	4.473	−0.233	9.178	0.062	
**L insula thk ×10[mm]**	−0.881	−3.755	1.994	0.543	**0.550**
Subgroup IPS impaired (Ref. IPS preserved)	**265.470**	**104.547**	**426.393**	**0.002**	
Interaction (L insula thk ×10[mm], Subgroup)	**−8.826**	**−15.023**	**−2.629**	**0.006**	
Age	0.443	−0.032	0.918	0.067	
Sex (Ref. female)	**10.186**	**0.899**	**19.473**	**0.032**	
Education	0.716	−1.078	2.510	0.428	
EDSS	5.226	−0.107	10.559	0.055	
**SDMT**					
**L insula thk ×10[mm]**	1.229	−0.159	2.616	0.082	**0.497**
Subgroup IPS impaired (Ref. IPS preserved)	**−103.767**	**−181.43**	**−26.107**	**0.010**	
Interaction (L insula thk ×10[mm] subgroup)	**3.580**	**0.589**	**6.571**	**0.020**	
Age	−0.057	−0.286	0.173	0.623	
Sex (Ref. female)	**−5.269**	**−9.751**	**−0.787**	**0.022**	
Education (years)	**1.372**	**0.506**	**2.238**	**0.002**	
EDSS	**−2.785**	**−5.359**	**−0.212**	**0.034**	

*Abbreviations*: *AD*, axial diffusivity; *CI*, confidence interval; *CTT*, Color Trails Test; *e-TIV*, estimated total intracranial volume; *FA*, fractional anisotropy; *IPS*, information processing speed; *L*, left hemisphere; *mm*, millimeters; *R*, right hemisphere; *R*^2^, coefficient of determination; *s*, second; *SDMT*, Symbol Digit Modalities Test; *thk*, thickness; *vol*, volume normalized to estimated total intracranial volume

Bolded entries represents values the p value reached significance (*p*<0.05)

A significant interaction between the subgroups (IPS preserved and IPS impaired) and MRI volumetric metrics indicated that the relationship between MRI measures and IPS outcome differed depending on the subgroup (Table 3 and Supplement 3). We found a correlation between the results of CTT-2 and L thalamus vol. (*p*=0.044), R thalamus vol. (*p*<0.001), and L insula CTT-2 (*p*=0.006). The SDMT results were significantly associated with L insula vol. (*p*=0.02). None of the analyzed volumetric MRI data showed significance for the CTT-1 and PASAT scores. Furthermore, we acknowledged the significant contribution of age, gender (female), education, and EDSS to IPS deficit Fig.1.

## Discussion

In this cross-sectional study, we endeavor to exclusively evaluate multimodal MRI radiological contributors of IPS deterioration among RRMS patients. We used TRACULA, which is an automated probabilistic tractography, to reconstruct 18 major white matter tracts to detect alterations in WM microstructure. We also assessed selected brain volumetric measurements such as cortical thickness, thalamic vol., WMH vol., and NBV contribution to IPS deficit. In general, we confirmed that IPS deterioration is attributable to some crucial network abnormalities and brain atrophy; specifically, we defined particular WM tracts where structural dysconnectivity might be detrimental for IPS. These include R ILF, L SLFT, R UNC, FMAJ, FMIN, and R CST.

In particular, we observed that R ILF FA was associated with the CTT-2 results, whereas R ILF AD with the SDMT scores. Some of these findings corroborate previous studies [12, 44] evaluating tract-specific components of cognitive deficit in MS. The ILF is one of the major associative pathways connecting the occipital and temporal-occipital areas of the brain with anterior temporal areas, with marked lateralization in the R hemisphere [45]. Previous studies found this bidirectional tract to be crucial in processing and modulating visual cues and thus visually guided decisions and behaviors [46]. In our cohort, the CTT-1 results were associated with L SLFT FA. The superior longitudinal fasciculus (SLF) is considered to be the largest associative fiber bundle system in the brain [47]. The potential functions of the SLF described by the authors of previous studies [47] were motor planning of language function, syntactic processing during language production, regulating the focus of attention in spatial orientation, spatial awareness functioning, and spatial coordinates of upper limbs. Hence, the SLF could be indeed involved in information processing speed, assessed in CCT-1. We also found a significant contribution of CST FA to IPS, assessed in SDMT, which is again consistent with the recent findings of Govindarajan et al. (2021) [48]. An additional finding was the relevance of R UNC AD to CTT-2 and SDMT results. Although the functional role of UNC is still not fully elucidated, it is the major tract connecting the anterior part of the temporal lobe with the orbitofrontal cortex and frontopolar cortex, associated with executive function. In agreement with previous studies [49], R UNC AD was a contributor of the CCT-2 score. UNC together with corpus callosum FA was important contributors of IPS in both adults and the pediatric onset of MS [50]. Our study, furthermore, acknowledges the important role of CC in IPS deterioration. CC is the major interhemispheric WM commissure, influencing processing speed, visuospatial processing, and attention [51]. In agreement with previous studies [44], in our population, a significant correlation was present between FMAJ FA, and to a lesser degree also FMIN FA, and the results obtained in CTT-2.

Brain atrophy is consistently associated with cognitive deterioration in MS [52-54]. This well-known observation corresponds with some of our findings such as that thalamic atrophy plays a crucial role in the deterioration of processing speed, at least in our patient cohort, which also corroborates previous findings (Bergsland et al., 2016 and Houtchens et al., 2007) [52, 55]. The thalamus contains associative nuclei that are in a large portion related to cognitive performance with relatively little functional compensation mechanisms following its degeneration, in addition to that resulting from MS specific pathologies [52]. In this study, furthermore, we observed that altered L insular cortical thickness was associated with IPS. The insula receives afferents from some sensory thalamic nuclei and connects with the amygdala, limbic, and association cortical areas [56]. Association IPS and insular cortical thickness may arise from its involvement in a large number of different functions such as auditory processing, somatosensory, and somatomotor control [56].

In general, our study results add significant support to the concept whereby the disruption of selected WM tracts together with a regional brain atrophy may be the background for IPS deficit in RRMS, making the non-focal progression of the disease relatively straightforward to quantify and thus to monitor.

## Conclusion and limitations

Our results indicate that regional brain atrophy together with the WM tract disruption of R ILF, L SLFT, R UNC, R CST, FMAJ, and FMIN are essential contributors of slowed IPS in the RRMS patient population.

This study has several limitations, including its cross-sectional character and lack of control group. Moreover, in our cohort, there were patients with quite a heterogeneity of disease duration, disability, and age. MRI limitations include mainly the anisotropic voxel size, and potential head movements, which could alter the DTI and volumetric measurements, which we only monitored visually. Although the movement was taken into account in the analysis, it might still alter the metrics. Nevertheless, our work sheds new light onto neuroradiological contributors of IPS deficit, demonstrated in multimodal MRI techniques.

## Supplementary information

Supplementary file 1 Table 1. Demographic, clinical and MRI characteristics of the IPS preserved and IPS impaired subgroups of RRMS patients

Supplementary file 2 Table 2. Univariate linear regression in subgroups: IPS preserved and IPS impaired, for the endpoint SDMT

Supplementary file 3 Table 3. Multiple regression models with MRI measures, IPS preserved and IPS impaired subgroups, and interaction between them for cognitive tests

## References

[1] Chiaravalloti ND, DeLuca J (2008). Cognitive impairment in multiple sclerosis. Lancet Neurol.

[2] Charcot JM (1877). Lectures on the diseases of the nervous system.

[3] Preziosa P, Rocca MA, Pagani E (2016). Structural MRI correlates of cognitive impairment in patients with multiple sclerosis: a multicenter study. Hum Brain Mapp.

[4] Benedict RHB, Cookfair D, Gavett R (2006). Validity of the minimal assessment of cognitive function in multiple sclerosis (MACFIMS). J Int Neuropsychol Soc.

[5] Julian L, Serafin D, Charvet L (2013). Cognitive impairment occurs in children and adolescents with multiple sclerosis: results from a United States network. J Child Neurol.

[6] Costa SL, Genova HM, DeLuca J, Chiaravalloti ND (2017). Information processing speed in multiple sclerosis: past, present, and future. Mult Scler J.

[7] Benedict RH, DeLuca J, Phillips G (2017). Validity of the Symbol Digit Modalities Test as a cognition performance outcome measure for multiple sclerosis. Mult Scler J.

[8] Gronwall DMA (1977). Paced auditory serial-addition task: a measure of recovery from concussion. Percept Mot Skills.

[9] Manca R, Sharrack B, Paling D, Wilkinson ID, Venneri A (2018). Brain connectivity and cognitive processing speed in multiple sclerosis: a systematic review. J Neurol Sci.

[10] Buyukturkoglu K, Zeng D, Bharadwaj S (2021). Classifying multiple sclerosis patients on the basis of SDMT performance using machine learning. Mult Scler.

[11] Rao SM, Martin AL, Huelin R (2014). Correlations between MRI and information processing speed in MS: a meta-analysis. Mult Scler Int.

[12] Dineen RA, Vilisaar J, Hlinka J (2009). Disconnection as a mechanism for cognitive dysfunction in multiple sclerosis. Brain.

[13] Sullivan EV, Pfefferbaum A (2006). Diffusion tensor imaging and aging. Neurosci Biobehav Rev.

[14] Giorgio A, Palace J, Johansen-Berg H (2010). Relationships of brain white matter microstructure with clinical and MR measures in relapsing-remitting multiple sclerosis. J Magn Reson Imaging.

[15] Fink F, Klein J, Lanz M (2010). Comparison of diffusion tensor-based tractography and quantified brain atrophy for analyzing demyelination and axonal loss in MS. J Neuroimaging.

[16] Goldberg-Zimring D, Mewes AUJ, Maddah M, Warfield SK (2005). Diffusion tensor magnetic resonance imaging in multiple sclerosis. J Neuroimaging.

[17] Schmierer K, Wheeler-Kingshott CAM, Boulby PA (2007). Diffusion tensor imaging of post mortem multiple sclerosis brain. NeuroImage.

[18] Roosendaal S, Geurts J, Vrenken H (2009). Regional DTI differences in multiple sclerosis patients. NeuroImage.

[19] Mesaros S, Rocca MA, Kacar K (2012). Diffusion tensor MRI tractography and cognitive impairment in multiple sclerosis. Neurology.

[20] Filippi M, Preziosa P, Rocca MA (2018). MRI in multiple sclerosis: what is changing?. Curr Opin Neurol.

[21] Rocca MA, Amato MP, De Stefano N (2015). Clinical and imaging assessment of cognitive dysfunction in multiple sclerosis. Lancet Neurol.

[22] Steenwijk MD, Geurts JJG, Daams M (2016). Cortical atrophy patterns in multiple sclerosis are non-random and clinically relevant. Brain.

[23] Bisecco A, Stamenova S, Caiazzo G (2018). Attention and processing speed performance in multiple sclerosis is mostly related to thalamic volume. Brain Imaging Behav.

[24] Yendiki A (2011). Automated probabilistic reconstruction of white-matter pathways in health and disease using an atlas of the underlying anatomy. Front Neuroinform.

[25] Polman CH, Reingold SC, Banwell B (2011). Diagnostic criteria for multiple sclerosis: 2010 revisions to the McDonald criteria. Ann Neurol.

[26] Kurtzke JF (1983). Rating neurological impairment in multiple sclerosis: an expanded disability status scale (EDSS). Neurology.

[27] Vogel A, Stokholm J, Jørgensen K (2013). Performances on Symbol Digit Modalities Test, Color Trails Test, and modified Stroop test in a healthy, elderly Danish sample. Aging Neuropsychol Cogn.

[28] D’Elia LF (1996). Satz P.

[29] Fischer JS, Jak AJ, Kniker J. E., Rudick R.A., Cutter G. Multiple Sclerosis Functional Composite (MSFC): Scoring Instructions. Archives of Neuropsychiatry, 2018. 55(Suppl 1):S46Published online 2001. http://main.nationalmssociety.org/docs/HOM/MSFC_Manual_and_Forms.pdf

[30] Beck AT, Steer RA, Brown GK (1987). Beck Depression Inventory.

[31] Beck AT, Steer RA, Brown GK BDI-II Inwentarz Depresji Becka – Wydanie Drugie. wydanie drugie. Pracownia Testów Psychologicznych www.practest.com.pl

[32] Pierpaoli C, Jezzard P, Basser PJ, Barnett A, Di Chiro G (1996). Diffusion tensor MR imaging of the human brain. Radiology.

[33] Nunnari D, De Cola MC, D’Aleo G (2015). Impact of depression, fatigue, and global measure of cortical volume on cognitive impairment in multiple sclerosis. BioMed Res Int.

[34] Louis F, D’Elia SP, Lyons C, Łojek E, Stańczak JCTT (2012). Kolorowy Test Połączeń wersja dla Dorosłych.

[35] Smith A (1982). Symbol Digit Modalities Test (SDMT). Manual (Revised).

[36] Fischl B, Sereno MI, Dale A (1999). Cortical surface-based analysis: II: inflation, flattening, and a surface-based coordinate system. NeuroImage.

[37] Fischl B, Sereno MI, Tootell RBH, Dale AM (1999). High-resolution intersubject averaging and a coordinate system for the cortical surface. Hum Brain Mapp.

[38] Reuter M, Rosas HD, Fischl B (2010). Highly accurate inverse consistent registration: a robust approach. NeuroImage.

[39] Fischl B, Salat DH, Busa E (2002). Whole brain segmentation: automated labeling of neuroanatomical structures in the human brain. Neuron.

[40] Fischl B, van der Kouwe A, Destrieux C (2004). Automatically parcellating the human cerebral cortex. Cereb Cortex.

[41] Sled JG, Zijdenbos AP, Evans AC (1998). A nonparametric method for automatic correction of intensity nonuniformity in MRI data. IEEE Trans Med Imaging.

[42] Fischl B, Liu A, Dale AM (2001). Automated manifold surgery: constructing geometrically accurate and topologically correct models of the human cerebral cortex. IEEE Med Imaging.

[43] Segonne F, Pacheco J, Fischl B (2007). Geometrically accurate topology-correction of cortical surfaces using nonseparating loops. IEEE Trans Med Imaging.

[44] Genova HM, DeLuca J, Chiaravalloti N, Wylie G (2013). The relationship between executive functioning, processing speed, and white matter integrity in multiple sclerosis. J Clin Exp Neuropsychol.

[45] Latini F, Mårtensson J, Larsson EM (2017). Segmentation of the inferior longitudinal fasciculus in the human brain: a white matter dissection and diffusion tensor tractography study. Brain Res.

[46] Herbet G, Zemmoura I, Duffau H (2018). Functional anatomy of the inferior longitudinal fasciculus: from historical reports to current hypotheses. Front Neuroanat.

[47] Janelle F, Iorio-Morin C, D’amour S, Fortin D (2022). Superior longitudinal fasciculus: a review of the anatomical descriptions with functional correlates. Front Neurol.

[48] Govindarajan ST, Liu Y, Parra Corral MA (2021). White matter correlates of slowed information processing speed in unimpaired multiple sclerosis patients with young age onset. Brain Imaging Behav.

[49] Meijer KA, Muhlert N, Cercignani M (2016). White matter tract abnormalities are associated with cognitive dysfunction in secondary progressive multiple sclerosis. Mult Scler J.

[50] Bartlett E, Shaw M, Schwarz C (2019). Brief computer-based information processing measures are linked to white matter integrity in pediatric-onset multiple sclerosis: pediatric multiple sclerosis and DTI imaging. J Neuroimaging.

[51] Huynh-Le MP, Tibbs MD, Karunamuni R (2021). Microstructural injury to corpus callosum and intrahemispheric white matter tracts correlate with attention and processing speed decline after brain radiation. Int J Radiat Oncol.

[52] Houtchens MK, Benedict RHB, Killiany R (2007). Thalamic atrophy and cognition in multiple sclerosis. Neurology.

[53] Benedict RHB, Amato MP, DeLuca J, Geurts JJG (2020). Cognitive impairment in multiple sclerosis: clinical management, MRI, and therapeutic avenues. Lancet Neurol.

[54] Benedict RHB, Weinstock-Guttman B, Fishman I, Sharma J, Tjoa CW, Bakshi R (2004). Prediction of neuropsychological impairment in multiple sclerosis: comparison of conventional magnetic resonance imaging measures of atrophy and lesion burden. Arch Neurol.

[55] Bergsland N, Zivadinov R, Dwyer MG, Weinstock-Guttman B, Benedict RH (2016). Localized atrophy of the thalamus and slowed cognitive processing speed in MS patients. Mult Scler J.

[56] Nieuwenhuys R (2012) The insular cortex: a review. Elsevier. Prog Brain Res 195:123–163. 10.1016/B978-0-444-53860-4.00007-6 10.1016/B978-0-444-53860-4.00007-6 22230626

