# Supplementary material for: The association between white matter tract structural connectivity and information processing speed in relapsing-remitting multiple sclerosis
Source: Neurol Sci. 2023 Apr 27;44(9):3221–32. doi: 10.1007/s10072-023-06817-6 (PMC10415523; doi:10.1007/s10072-023-06817-6)
Supplement: Supplementary file 1 — Table 1. Demographic, clinical and MRI characteristics of the IPS preserved and IPS impaired subgroups of RRMS patients [file 10072_2023_6817_MOESM1_ESM.docx]

| **TABLE 1.** Demographic, clinical and MRI characteristics of the IPS preserved and IPS impaired subgroups of RRMS patients | | | | |
| --- | --- | --- | --- | --- |
| **Clinical measures** | IPS preserved  **(N=57)** | IPS impaired **(N=16)** | | ***p*-value** |
| Age [years] |  | |  | 0.405 |
| Mean (SD) | 37.84 (9.61) | | 40.0 (6.96) |  |
| Median (Q1,Q3) | 39.0 (29.0, 45.0) | | 44.0 (34.0, 45.0) |  |
| Female sex, n (%) | 42 (73.7) | | 11 (68.8) | 0.696 |
| Disease duration [years] |  | |  | 0.136 |
| Mean (SD) | 8.14 (4.194) | | 10.32 (7.552) |  |
| Median (Q1,Q3) | 9.0 (5.0, 10.0) | | 9.5 (4.000, 14.250) |  |
| Education [years] |  | |  | 0.179 |
| Mean (SD) | 15.18 (2.346) | | 14.25 (2.646) |  |
| Median (Q1,Q3) | 16.0 (13.0, 17.0) | | 13.5 (12.0, 17.0) |  |
| EDSS |  | |  | **0.011** |
| Mean (SD) | 2.333 (0.883) | | 3.000 (0.949) |  |
| Median (Q1,Q3) | 2.500 (1.500, 2.500) | | 3.000 (2.375, 3.500) |  |
| SDMT |  | |  | **< 0.001** |
| Mean (SD) | 48.737 (9.83) | | 34.562 (11.87) |  |
| Median (Q1,Q3) | 49.0 (44.0, 54.0) | | 34.50 (25.75, 46.25) |  |
| PASAT |  | |  | **< 0.001** |
| Mean (SD) | 47.054 (8.158) | | 35.40 (13.426) |  |
| Median (Q1,Q3) | 48.0 (41.75, 54.0) | | 33.0 (27.50, 47.50) |  |
| CTT 1 time |  | |  | **< 0.001** |
| Mean (SD) | 35.684 (11.466) | | 57.250 (20.358) |  |
| Median (Q1,Q3) | 34.0 (27.0, 41.0) | | 55.000 (44.0, 65.25) |  |
| CTT 2 time |  | |  | **< 0.001** |
| Mean (SD) | 74.930 (16.736) | | 114.938 (28.899) |  |
| Median (Q1,Q3) | 76.0 (61.0, 85.0) | | 113.000 (98.0, 132.75) |  |
| **MRI measures** |  | |  |  |
| L Thalamus vol x10^-4^ |  | |  | **0.013** |
| Mean (SD) | 46.203 (4.575) | | 42.527 (6.791) |  |
| Median (Q1,Q3) | 45.425 (44.185, 48.413) | | 45.665 (36.793, 48.567) |  |
| R Thalamus vol x10^-4^ |  | |  | **< 0.001** |
| Mean (SD) | 43.147 (4.465) | | 38.455 (5.587) |  |
| Median (Q1,Q3) | 43.218 (41.424, 45.329) | | 38.083 (32.907, 43.159) |  |
| WMH vol x10^-4^ |  | |  | **0.004** |
| Mean (SD) | 27.754 (18.755) | | 50.121 (45.011) |  |
| Median (Q1,Q3) | 20.015 (13.760, 36.804) | | 32.830 (20.834, 61.08) |  |
| CC central division vol x10^-5^ |  | |  | 0.250 |
| Mean (SD) | 34.768 (11.433) | | 30.807 (14.203) |  |
| Median (Q1,Q3) | 32.791 (26.259, 42.969) | | 28.153 (19.368, 41.454) |  |
| NBV x10^-2^ |  | |  | 0.058 |
| Mean (SD) | 73.737 (4.129) | | 71.374 (5.002) |  |
| Median (Q1,Q3) | 74.269 (71.501, 75.965) | | 73.378 (67.091, 75.712) |  |
| L UNC AD x10^-5^ |  | |  | 0.119 |
| Mean (SD) | 112.743 (3.955) | | 114.754 (6.144) |  |
| Median (Q1,Q3) | 112.895 (109.91, 115.04) | | 114.091 (109.99, 119.77) |  |
| R UNC AD x10^-5^[mm^2^/s] |  | |  | 0.028 |
| Mean (SD) | 110.176 (5.247) | | 113.435 (4.674) |  |
| Median (Q1,Q3) | 110.777 (107.866, 113.615) | | 114.145 (110.21, 115.688) |  |
| R ILF FA x10^-2^ |  | |  | 0.127 |
| Mean (SD) | 39.271 (3.924) | | 37.577 (3.684) |  |
| Median (Q1,Q3) | 39.605 (36.977, 42.202) | | 37.959 (34.431, 40.543) |  |
| L CAB FA x10^-2^ |  | |  | 0.355 |
| Mean (SD) | 30.834 (4.949) | | 29.533 (4.910) |  |
| Median (Q1,Q3) | 30.291 (26.760, 34.225) | | 29.933 (25.998, 31.142) |  |
| L pars opercularis cortical thk.[mm] |  | |  | **< 0.001** |
| Mean (SD) | 2.373 (0.174) | | 2.195 (0.160) |  |
| Median (Q1,Q3) | 2.340 (2.267, 2.482) | | 2.203 (2.152, 2.275) |  |
| L insula cortical thk.[mm] |  | |  | 0.666 |
| Mean (SD) | 2.617 (0.177) | | 2.596 (0.166) |  |
| Median (Q1,Q3) | 2.626 (2.498, 2.732) | | 2.591 (2.463, 2.672) |  |
| L CAB AD x10^-2^[mm^2^/s] |  | |  | 0.693 |
| Mean (SD) | 108.495 (6.901) | | 109.259 (6.467) |  |
| Median (Q1,Q3) | 108.824 (104.442, 113.210) | | 107.972(105.003,113.112) |  |
| L CAB FA x10^-2^ |  | |  | 0.355 |
| Mean (SD) | 30.834 (4.949) | | 29.533 (4.910) |  |
| Median (Q1,Q3) | 30.291 (26.760, 34.225) | | 29.933 (25.998, 31.142) |  |
| L UNC AD x10^-5^[mm^2^/s] |  | |  | 0.119 |
| Mean (SD) | 112.743 (3.955) | | 114.754 (6.144) |  |
| Median (Q1,Q3) | 112.895 (109.909, 115.041) | | 114.091 (109.991, 119.77) |  |
| R ILF FA x10^-2^ |  | |  | 0.127 |
| Mean (SD) | 39.271 (3.924) | | 37.577 (3.684) |  |
| Median (Q1,Q3) | 39.605 (36.977, 42.202) | | 37.959 (34.431, 40.543) |  |
| R ILF AD x10^-5^[mm^2^/s] |  | |  | 0.708 |
| Mean (SD) | 118.735 (5.897) | | 119.359 (5.800) |  |
| Median (Q1,Q3) | 119.053 (115.387, 121.688) | | 118.478 (114.975, 122.18) |  |
| L isthmus cingulate thk.[mm]x10^-1^ |  | |  | 0.152 |
| Mean (SD) | 21.434 (1.952) | | 20.656 (1.676) |  |
| Median (Q1,Q3) | 21.390 (20.100, 22.470) | | 20.520 (19.652, 21.958) |  |
| R ATR AD x10^-3^[mm^2^/s] |  | |  | 0.168 |
| Mean (SD) | 1.119 (0.043) | | 1.137 (0.056) |  |
| Median (Q1,Q3) | 1.123 (1.095, 1.145) | | 1.142 (1.094, 1.181) |  |
| R ATR FA x10^-1^ |  | |  | 0.695 |
| Mean (SD) | 4.061 (0.230) | | 4.035 (0.239) |  |
| Median (Q1,Q3) | 4.060 (3.899, 4.223) | | 3.980 (3.899, 4.160) |  |
| L ATR FA x10^-1^ |  | |  | 0.484 |
| Mean (SD) | 4.126 (0.236) | | 4.174 (0.264) |  |
| Median (Q1,Q3) | 4.068 (3.974, 4.270) | | 4.182 (4.061, 4.346) |  |
| L ATR AD x10^-3^[mm^2^/s] |  | |  | 0.187 |
| Mean (SD) | 1.110 (0.050) | | 1.130 (0.055) |  |
| Median (Q1,Q3) | 1.110 (1.087, 1.138) | | 1.128 (1.079, 1.161) |  |
| L CCG AD x10^-3^[mm^2^/s] |  | |  | 0.079 |
| Mean (SD) | 1.200 (0.085) | | 1.244 (0.101) |  |
| Median (Q1,Q3) | 1.208 (1.155, 1.259) | | 1.250 (1.199, 1.317) |  |
| L CCG FA x10^-1^ |  | |  | 0.560 |
| Mean (SD) | 4.817 (0.405) | | 4.748 (0.474) |  |
| Median (Q1,Q3) | 4.836 (4.514, 5.127) | | 4.633 (4.357, 5.095) |  |
| R CCG AD x 10^-3^[mm^2^/s] |  | |  | 0.880 |
| Mean (SD) | 1.148 (0.079) | | 1.151 (0.060) |  |
| Median (Q1,Q3) | 1.142 (1.110, 1.202) | | 1.139 (1.125, 1.188) |  |
| R CCG FA x 10^-1^ |  | |  | 0.262 |
| Mean (SD) | 4.503 (0.499) | | 4.334 (0.623) |  |
| Median (Q1,Q3) | 4.465 (4.196, 4.778) | | 4.299 (3.924, 4.745) |  |
| L CST AD x 10^-3^[mm^2^/s] |  | |  | 0.459 |
| Mean (SD) | 1.164 (0.039) | | 1.173 (0.050) |  |
| Median (Q1,Q3) | 1.162 (1.140, 1.184) | | 1.164 (1.141, 1.196) |  |
| L CST FA x 10^-1^ |  | |  | 0.581 |
| Mean (SD) | 4.879 (0.238) | | 4.919 (0.306) |  |
| Median (Q1,Q3) | 4.867 (4.711, 5.035) | | 4.983 (4.679, 5.140) |  |
| R CST FA x 10^-1^ |  | |  | 0.254 |
| Mean (SD) | 4.666 (0.224) | | 4.743 (0.274) |  |
| Median (Q1,Q3) | 4.675 (4.549, 4.779) | | 4.726 (4.523, 4.968) |  |
| R CST AD x 10^-3^[mm^2^/s] |  | |  | 0.459 |
| Mean (SD) | 1.164 (0.039) | | 1.173 (0.050) |  |
| Median (Q1,Q3) | 1.162 (1.140, 1.184) | | 1.164 (1.141, 1.196) |  |
| FMAJ FA x 10^-1^ |  | |  | 0.066 |
| Mean (SD) | 5.33 (0.39) | | 5.09 (0.57) |  |
| Median (Q1,Q3) | 5.37 (5.11, 5.60) | | 5.19 (4.92, 5.42) |  |
| FMIN FA x 10^-1^ |  | |  | 0.981 |
| Mean (SD) | 4.65 (0.41) | | 4.65 (0.30) |  |
| Median (Q1,Q3) | 4.65 (4.34, 0.493) | | 4.63 (4.45, 4.82) |  |
| SLFP FA x 10^-1^ |  | |  | 0.775 |
| Mean (SD) | 3.71 (0.27) | | 3.69 (0.23) |  |
| Median (Q1,Q3) | 3.72 (3.51, 3.87) | | 3.72 (3.50, 3.88) |  |
| SLFT FA x10^-1^ |  | |  | 0.911 |
| Mean (SD) | 4.01 (0.29) | | 4.02 (0.28) |  |
| Median (Q1,Q3) | 3.98 (3.83, 4.22) | | 4.06 (3.87, 4.20) |  |
| **Abbreviations**: AD, axial diffusivity; ATR, anterior thalamic radiation; CAB, cingulum–angular (infracallosal) bundle; CC, corpus callosum; CCG, cingulum–cingulate gyrus (supracallosal) bundle;; CST, corticospinal tract; CTT, Color Trails Test; EDSS, Expanded Disability Status Score; e-TIV, estimated total intracranial volume; FA, fractional anisotropy; FMAJ, corpus callosum–forceps major; FMIN, corpus callosum–forceps minor; ILF, inferior longitudinal fasciculus; IPS, information processing speed; L, left hemisphere; mm, millimetres; NBV, normalized brain volume; PASAT, Paced Auditory Serial Additive Test; R, right hemisphere; s second; SDMT Symbol Digit Modalities Test; SLFP, superior longitudinal fasciculus–parietal bundle; SLFT, superior longitudinal fasciculus–temporal bundle; thk, thickness; UNC, uncinate fasciculus; vol, volume normalized to estimated total intracranial volume; WMH, white matter hypointensities. | | | | |
