# Supplementary material for: The association between white matter tract structural connectivity and information processing speed in relapsing-remitting multiple sclerosis
Source: Neurol Sci. 2023 Apr 27;44(9):3221–32. doi: 10.1007/s10072-023-06817-6 (PMC10415523; doi:10.1007/s10072-023-06817-6)
Supplement: Supplementary file 2 — Table 2. Univariate linear regression in subgroups: IPS preserved and IPS impaired, for the endpoint SDMT [file 10072_2023_6817_MOESM2_ESM.docx]

| **Table 2.** Univariate linear regression in subgroups: IPS preserved and IPS impaired, for the endpoint SDMT   \|  \| **SDMT** \| **Estimate**  **(Beta coefficient)** \| **Lower**  **95% CI** \| **Upper**  **95% CI** \| ***p*- value** \| **R^2^** \| \| --- \| --- \| --- \| --- \| --- \| --- \| --- \| \| **CP** \| L CAB AD x10^-5^[mm^2^/s] \| 0.217 \| -0.163 \| 0.598 \| 0.257 \| 0.023 \| \|  \| L CAB FA x10^-2^ \| -0.221 \| -0.754 \| 0.313 \| 0.411 \| 0.012 \| \|  \| L UNC AD x10^-5^[mm^2^/s] \| -0.749 \| -1.390 \| -0.109 \| **0.023** \| **0.091** \| \|  \| R ILF FA x10^-2^ \| 0.237 \| -0.437 \| 0.911 \| 0.484 \| 0.009 \| \|  \| R ILF FA x10^-5^ \| 0.372 \| -0.067 \| 0.811 \| 0.095 \| 0.050 \| \|  \| R UNC AD x10^-5^[mm^2^/s] \| -0.292 \| -0.792 \| 0.208 \| 0.247 \| 0.024 \| \|  \| R SLFP FA x10^-2^ \| -0.367 \| -1.443 \| 0.709 \| 0.496 \| 0.009 \| \|  \| L SLFT FA x10^-2^ \| -0.769 \| -1.730 \| 0.191 \| 0.114 \| 0.048 \| \|  \| FMAJ FA x10^-2^ \| -0.275 \| -1.009 \| 0.458 \| 0.454 \| 0.011 \| \|  \| FMIN FA x10^-2^ \| -0.698 \| -1.378 \| -0.018 \| **0.044** \| **0.077** \| \|  \| L pars opercularis thk[mm] x10^1^ \| 1.880 \| 0.437 \| 3.323 \| **0.012** \| **0.110** \| \|  \| L isthmus cingulate thk[mm] x10^1^ \| 1.638 \| 0.351 \| 2.925 \| **0.014** \| **0.106** \| \|  \| L insula thk[mm] x 10^1^ \| 2.016 \| 0.617 \| 3.415 \| **0.006** \| **0.132** \| \|  \| L thalamus vol x 10^4^ \| 0.738 \| 0.192 \| 1.283 \| **0.009** \| **0.118** \| \|  \| R thalamus vol x 10^4^ \| 0.698 \| 0.134 \| 1.262 \| **0.016** \| **0.100** \| \|  \| WMH vol x10^4^ \| -0.173 \| -0.307 \| -0.040 \| **0.012** \| **0.110** \| \|  \| CC Central vol x 10^5^ \| 0.273 \| 0.053 \| 0.493 \| **0.016** \| **0.101** \| \|  \| NBV x 10 \| 0.674 \| 0.057 \| 1.291 \| **0.033** \| **0.080** \| \|  \| R ATR AD x 10^-3^[mm^2^/s] \| 2.400 \| -2.707 \| 7.507 \| 0.350 \| 0.016 \| \|  \| R ATR FA x 10^-1^ \| -0.234 \| -1.184 \| 0.717 \| 0.624 \| 0.004 \| \|  \| L ATR FA x 10^-1^ \| -0.344 \| -1.267 \| 0.580 \| 0.459 \| 0.010 \| \|  \| L ATR AD x 10^-3^[mm^2^/s] \| 0.141 \| -4.231 \| 4.512 \| 0.949 \| 0.000 \| \|  \| L CCG AD x 10^-3^[mm^2^/s] \| -0.890 \| -3.469 \| 1.689 \| 0.492 \| 0.009 \| \|  \| L CCG FA x 10^-1^ \| -0.518 \| -1.040 \| 0.005 \| 0.052 \| 0.067 \| \|  \| R CCG AD x 10^-3^[mm^2^/s] \| 0.318 \| -2.449 \| 3.085 \| 0.819 \| 0.001 \| \|  \| R CCG FA x 10^-1^ \| -0.219 \| -0.654 \| 0.215 \| 0.316 \| 0.018 \| \|  \| L CST AD x 10^-3^[mm^2^/s] \| 3.647 \| -1.864 \| 9.157 \| 0.190 \| 0.031 \| \|  \| L CST FA x 10^-1^ \| 0.347 \| -0.568 \| 1.261 \| 0.451 \| 0.010 \| \|  \| R CST FA x 10^-1^ \| -0.335 \| -1.310 \| 0.639 \| 0.493 \| 0.009 \| \|  \| R CST AD x 10^-3^[mm^2^/s] \| -0.387 \| -7.230 \| 6.456 \| 0.910 \| 0.000 \| \| **CI** \| L CAB AD x 10^-5^[mm^2^/s] \| 0.089 \| -0.962 \| 1.141 \| 0.858 \| 0.002 \| \|  \| L CAB FA x10^-2^ \| 0.283 \| -1.093 \| 1.660 \| 0.665 \| 0.014 \| \|  \| L UNC AD x10^-5^[mm^2^/s] \| -0.924 \| -1.897 \| 0.049 \| 0.061 \| 0.229 \| \|  \| R ILF FA x10^-2^ \| 0.882 \| -0.895 \| 2.659 \| 0.305 \| 0.075 \| \|  \| R ILF AD x10^-5^[mm^2^/s] \| -0.999 \| -2.023 \| 0.024 \| 0.055 \| 0.238 \| \|  \| R UNC AD x10^-5^[mm^2^/s] \| -1.426 \| -2.630 \| -0.221 \| **0.024** \| **0.315** \| \|  \| R SLFP FA x10^-2^ \| 1.749 \| -1.305 \| 4.803 \| 0.238 \| 0.105 \| \|  \| L SLFT FA x10^-2^ \| 0.562 \| -2.025 \| 3.150 \| 0.647 \| 0.017 \| \|  \| FMAJ FA x10^-2^ \| 1.070 \| -0.055 \| 2.195 \| 0.061 \| 0.245 \| \|  \| FMIN FA x10^-2^ \| -0.364 \| -2.814 \| 2.086 \| 0.753 \| 0.008 \| \|  \| L pars opercularis thk [mm]x10^-1^ \| 3.599 \| -0.114 \| 7.313 \| 0.057 \| 0.236 \| \|  \| L isthmus cingulate thk[mm] x10^-1^ \| 1.768 \| -2.163 \| 5.698 \| 0.351 \| 0.062 \| \|  \| L insula thk[mm] x10^-1^ \| 3.927 \| 0.509 \| 7.344 \| **0.027** \| **0.303** \| \|  \| L thalamus vol x 10^-3^ \| 1.199 \| 0.470 \| 1.928 \| **0.003** \| **0.471** \| \|  \| R thalamus vol x 10^-4^ \| 1.336 \| 0.389 \| 2.283 \| **0.009** \| **0.395** \| \|  \| WMH vol x10^-4^ \| -0.109 \| -0.247 \| 0.028 \| 0.111 \| 0.171 \| \|  \| CC Central vol x10^-5^ \| 0.556 \| 0.199 \| 0.914 \| **0.005** \| **0.443** \| \|  \| NBV x10^-2^ \| 1.319 \| 0.188 \| 2.450 \| **0.025** \| **0.309** \| \|  \| R ATR AD x 10^-3^[mm^2^/s] \| -0.264 \| -6.210 \| 5.682 \| 0.926 \| 0.001 \| \|  \| R ATR FA x 10^-1^ \| -0.309 \| -1.679 \| 1.061 \| 0.636 \| 0.016 \| \|  \| L ATR FA x 10^-1^ \| -0.445 \| -1.669 \| 0.778 \| 0.448 \| 0.042 \| \|  \| L ATR AD x 10^-3^[mm^2^/s] \| -2.391 \| -8.253 \| 3.471 \| 0.396 \| 0.052 \| \|  \| L CCG AD x 10^-3^[mm^2^/s] \| 1.656 \| -1.474 \| 4.786 \| 0.275 \| 0.084 \| \|  \| L CCG FA x 10^-1^ \| -0.111 \| -0.804 \| 0.583 \| 0.737 \| 0.008 \| \|  \| R CCG AD x 10^-3^[mm^2^/s] \| 1.883 \| -3.530 \| 7.295 \| 0.468 \| 0.038 \| \|  \| R CCG FA x 10^-1^ \| -0.175 \| -0.695 \| 0.346 \| 0.484 \| 0.036 \| \|  \| L CST AD x 10^-4^[mm^2^/s] \| 0.124 \| -6.415 \| 6.664 \| 0.968 \| 0.000 \| \|  \| L CST FA x 10^-1^ \| -0.644 \| -1.659 \| 0.370 \| 0.195 \| 0.117 \| \|  \| R CST FA x 10^-1^ \| -0.699 \| -1.833 \| 0.436 \| 0.208 \| 0.111 \| \|  \| R CST AD x 10^-3^[mm^2^/s] \| -2.048 \| -7.610 \| 3.513 \| 0.443 \| 0.043 \| |
| --- | --- | --- | --- | --- | --- | --- | --- | --- | --- | --- | --- | --- | --- | --- | --- | --- | --- | --- | --- | --- | --- | --- | --- | --- | --- | --- | --- | --- | --- | --- | --- | --- | --- | --- | --- | --- | --- | --- | --- | --- | --- | --- | --- | --- | --- | --- | --- | --- | --- | --- | --- | --- | --- | --- | --- | --- | --- | --- | --- | --- | --- | --- | --- | --- | --- | --- | --- | --- | --- | --- | --- | --- | --- | --- | --- | --- | --- | --- | --- | --- | --- | --- | --- | --- | --- | --- | --- | --- | --- | --- | --- | --- | --- | --- | --- | --- | --- | --- | --- | --- | --- | --- | --- | --- | --- | --- | --- | --- | --- | --- | --- | --- | --- | --- | --- | --- | --- | --- | --- | --- | --- | --- | --- | --- | --- | --- | --- | --- | --- | --- | --- | --- | --- | --- | --- | --- | --- | --- | --- | --- | --- | --- | --- | --- | --- | --- | --- | --- | --- | --- | --- | --- | --- | --- | --- | --- | --- | --- | --- | --- | --- | --- | --- | --- | --- | --- | --- | --- | --- | --- | --- | --- | --- | --- | --- | --- | --- | --- | --- | --- | --- | --- | --- | --- | --- | --- | --- | --- | --- | --- | --- | --- | --- | --- | --- | --- | --- | --- | --- | --- | --- | --- | --- | --- | --- | --- | --- | --- | --- | --- | --- | --- | --- | --- | --- | --- | --- | --- | --- | --- | --- | --- | --- | --- | --- | --- | --- | --- | --- | --- | --- | --- | --- | --- | --- | --- | --- | --- | --- | --- | --- | --- | --- | --- | --- | --- | --- | --- | --- | --- | --- | --- | --- | --- | --- | --- | --- | --- | --- | --- | --- | --- | --- | --- | --- | --- | --- | --- | --- | --- | --- | --- | --- | --- | --- | --- | --- | --- | --- | --- | --- | --- | --- | --- | --- | --- | --- | --- | --- | --- | --- | --- | --- | --- | --- | --- | --- | --- | --- | --- | --- | --- | --- | --- | --- | --- | --- | --- | --- | --- | --- | --- | --- | --- | --- | --- | --- | --- | --- | --- | --- | --- | --- | --- | --- | --- | --- | --- | --- | --- | --- | --- | --- | --- | --- | --- | --- | --- | --- | --- | --- | --- | --- | --- | --- | --- | --- | --- | --- | --- | --- | --- | --- | --- | --- | --- | --- | --- | --- | --- | --- | --- | --- | --- | --- | --- | --- | --- | --- | --- | --- | --- | --- | --- | --- | --- | --- | --- | --- | --- | --- | --- | --- | --- | --- | --- | --- | --- | --- | --- | --- | --- | --- | --- | --- | --- | --- | --- | --- | --- | --- | --- | --- | --- | --- | --- | --- | --- | --- | --- | --- | --- | --- | --- | --- | --- | --- | --- | --- | --- | --- | --- | --- | --- | --- | --- | --- |
| **Abbreviations**: AD, axial diffusivity; ATR, anterior thalamic radiation; CAB, cingulum–angular (infracallosal) bundle; CC, corpus callosum; CCG, cingulum–cingulate gyrus (supracallosal) bundle; CST, corticospinal tract; e-TIV, estimated total intracranial volume; FA, fractional anisotropy; FMIN corpus callosum–forceps minor; ILF, inferior longitudinal fasciculus; IPS, information processing speed; L, left hemisphere; mm, millimetres; NBV, normalized brain volume; R, right hemisphere; s, second; SDMT, Symbol Digit Modalities Test; thk, thickness; UNC, uncinate fasciculus, FMAJ, corpus callosum–forceps major; vol, volume normalized to estimated total intracranial volume; WMH, white matter hypointensities. |

| **Table 2.** Univariate linear regression in subgroups: IPS preserved and IPS impaired, for the endpoint PASAT-3’ (z-cognitive)   \|  \| **PASAT-3, z-cognitive** \| **Estimate**  **(Beta coefficient)** \| **Lower**  **95% CI** \| **Upper**  **95% CI** \| **P value** \| **R2** \| \| --- \| --- \| --- \| --- \| --- \| --- \| --- \| \| **CP** \| L CAB AD x10^-5^[mm^2^/s] \| 0.043 \| -0.003 \| 0.088 \| 0.064 \| 0.061 \| \|  \| L CAB FA x10^-2^ \| 0.031 \| -0.034 \| 0.095 \| 0.346 \| 0.016 \| \|  \| L UNC AD x10^-5^[mm^2^/s] \| -0.003 \| -0.085 \| 0.078 \| 0.936 \| 0.000 \| \|  \| R ILF FA x10^-2^ \| -0.078 \| -0.158 \| 0.001 \| 0.053 \| 0.066 \| \|  \| R ILF FA x10^-5^ \| -0.016 \| -0.070 \| 0.039 \| 0.564 \| 0.006 \| \|  \| R UNC AD x10^-5^[mm^2^/s] \| 0.026 \| -0.036 \| 0.087 \| 0.406 \| 0.013 \| \|  \| R SLFP FA x10^-2^ \| 0.052 \| -0.027 \| 0.132 \| 0.191 \| 0.033 \| \|  \| L SLFT FA x10^-2^ \| 0.039 \| -0.034 \| 0.111 \| 0.292 \| 0.022 \| \|  \| Forceps major FA x10^-2^ \| 0.024 \| -0.031 \| 0.079 \| 0.381 \| 0.015 \| \|  \| Forceps minor FA x10^-2^ \| 0.013 \| -0.040 \| 0.065 \| 0.634 \| 0.004 \| \|  \| L pars opercularis thk[mm] x10^1^ \| -0.042 \| -0.227 \| 0.143 \| 0.650 \| 0.004 \| \|  \| L isthmus cingulate thk[mm] x10^1^ \| -0.111 \| -0.273 \| 0.052 \| 0.177 \| 0.033 \| \|  \| L insula thk[mm] x 10^1^ \| -0.042 \| -0.224 \| 0.140 \| 0.646 \| 0.004 \| \|  \| L thalamus vol x 10^4^ \| -0.042 \| -0.112 \| 0.028 \| 0.232 \| 0.026 \| \|  \| R thalamus vol x 10^4^ \| -0.035 \| -0.106 \| 0.037 \| 0.334 \| 0.017 \| \|  \| WM hypointensity vol x10^4^ \| 0.016 \| -0.001 \| 0.032 \| 0.065 \| 0.061 \| \|  \| CC Central vol x 10^5^ \| -0.017 \| -0.045 \| 0.011 \| 0.228 \| 0.026 \| \|  \| NBV x 10^2^ \| -0.030 \| -0.108 \| 0.047 \| 0.439 \| 0.011 \| \| **CI** \| L CAB AD x 10^-5^[mm^2^/s] \| 0.065 \| -0.235 \| 0.364 \| 0.650 \| 0.015 \| \|  \| L CAB FA x10^-2^ \| 0.142 \| -0.246 \| 0.531 \| 0.446 \| 0.042 \| \|  \| L UNC AD x10^-5^[mm^2^/s] \| -0.160 \| -0.463 \| 0.144 \| 0.279 \| 0.083 \| \|  \| R ILF FA x10^-2^ \| 0.089 \| -0.438 \| 0.616 \| 0.723 \| 0.009 \| \|  \| R ILF AD x10^-5^[mm^2^/s] \| -0.172 \| -0.493 \| 0.149 \| 0.270 \| 0.086 \| \|  \| R SLFP FA x10^-2^ \| -0.062 \| -0.272 \| 0.149 \| 0.539 \| 0.030 \| \|  \| L SLFT FA x10^-2^ \| 0.080 \| -0.086 \| 0.246 \| 0.316 \| 0.077 \| \|  \| Forceps major FA x10^-2^ \| -0.030 \| -0.114 \| 0.054 \| 0.459 \| 0.043 \| \|  \| Forceps minor FA x10^-2^ \| -0.102 \| -0.253 \| 0.050 \| 0.170 \| 0.139 \| \|  \| R UNC AD x10^-5^[mm^2^/s] \| -0.094 \| -0.508 \| 0.319 \| 0.632 \| 0.017 \| \|  \| L pars opercularis thk[mm] x10^1^ \| 0.519 \| -0.661 \| 1.699 \| 0.361 \| 0.060 \| \|  \| L isthmus cingulate thk[mm] x10^1^ \| 0.171 \| -0.988 \| 1.330 \| 0.756 \| 0.007 \| \|  \| L insula thk[mm] x10^1^ \| 1.275 \| 0.359 \| 2.192 \| **0.010** \| **0.389** \| \|  \| L thalamus vol x 10^3^ \| 0.180 \| -0.088 \| 0.448 \| 0.171 \| 0.130 \| \|  \| R thalamus vol x 10^4^ \| 0.257 \| -0.060 \| 0.573 \| 0.104 \| 0.178 \| \|  \| WM hypointensity vol x10^4^ \| -0.035 \| -0.074 \| 0.003 \| 0.067 \| 0.220 \| \|  \| CC Central vol x10^5^ \| 0.139 \| 0.027 \| 0.251 \| **0.018** \| **0.337** \| \|  \| NBV x10^2^ \| 0.218 \| -0.151 \| 0.587 \| 0.225 \| 0.103 \| |
| --- | --- | --- | --- | --- | --- | --- | --- | --- | --- | --- | --- | --- | --- | --- | --- | --- | --- | --- | --- | --- | --- | --- | --- | --- | --- | --- | --- | --- | --- | --- | --- | --- | --- | --- | --- | --- | --- | --- | --- | --- | --- | --- | --- | --- | --- | --- | --- | --- | --- | --- | --- | --- | --- | --- | --- | --- | --- | --- | --- | --- | --- | --- | --- | --- | --- | --- | --- | --- | --- | --- | --- | --- | --- | --- | --- | --- | --- | --- | --- | --- | --- | --- | --- | --- | --- | --- | --- | --- | --- | --- | --- | --- | --- | --- | --- | --- | --- | --- | --- | --- | --- | --- | --- | --- | --- | --- | --- | --- | --- | --- | --- | --- | --- | --- | --- | --- | --- | --- | --- | --- | --- | --- | --- | --- | --- | --- | --- | --- | --- | --- | --- | --- | --- | --- | --- | --- | --- | --- | --- | --- | --- | --- | --- | --- | --- | --- | --- | --- | --- | --- | --- | --- | --- | --- | --- | --- | --- | --- | --- | --- | --- | --- | --- | --- | --- | --- | --- | --- | --- | --- | --- | --- | --- | --- | --- | --- | --- | --- | --- | --- | --- | --- | --- | --- | --- | --- | --- | --- | --- | --- | --- | --- | --- | --- | --- | --- | --- | --- | --- | --- | --- | --- | --- | --- | --- | --- | --- | --- | --- | --- | --- | --- | --- | --- | --- | --- | --- | --- | --- | --- | --- | --- | --- | --- | --- | --- | --- | --- | --- | --- | --- | --- | --- | --- | --- | --- | --- | --- | --- | --- | --- | --- | --- | --- | --- | --- | --- | --- | --- | --- | --- | --- | --- | --- | --- | --- | --- | --- | --- |
| **Abbreviations**: AD, axial diffusivity; ATR, anterior thalamic radiation; CAB, cingulum–angular (infracallosal) bundle; CC, corpus callosum; CCG, cingulum–cingulate gyrus (supracallosal) bundle; CST, corticospinal tract; e-TIV, estimated total intracranial volume; FA, fractional anisotropy; ILF, inferior longitudinal fasciculus; IPS, information processing speed; L, left hemisphere; mm, millimetres; NBV, normalized brain volume; PASAT, Paced Auditory Serial Additive Test; R, right hemisphere; s, second; thk, thickness; UNC, uncinate fasciculus; vol, volume normalized to estimated total intracranial volume; WM, white matter. |

| **Tabel 3.** Univariate linear regression in subgroups: IPS preserved and IPS impaired, for the endpoints CTT1 and CTT2   \|  \| **CTT1** \| **Estimate**  **(Beta coefficient)** \| **Lower**  **95% CI** \| **Upper**  **95% CI** \| **P value** \| **R2** \| \| --- \| --- \| --- \| --- \| --- \| --- \| --- \| \| **CP** \| L CAB AD x10^-5^[mm^2^/s] \| -0.156 \| -0.603 \| 0.291 \| 0.487 \| 0.009 \| \|  \| L CAB FA x10^-2^ \| -0.044 \| -0.670 \| 0.582 \| 0.889 \| 0.000 \| \|  \| L UNC AD x10^-5^[mm^2^/s] \| -0.303 \| -1.082 \| 0.476 \| 0.440 \| 0.011 \| \|  \| R ILF FA x10^-2^ \| -0.077 \| -0.866 \| 0.713 \| 0.846 \| 0.001 \| \|  \| R ILF FA x10^-5^ \| -0.203 \| -0.726 \| 0.320 \| 0.440 \| 0.011 \| \|  \| R UNC AD x10^-5^[mm^2^/s] \| 0.133 \| -0.456 \| 0.723 \| 0.652 \| 0.004 \| \|  \| R ILF FA x10^-2^ \| -0.055 \| -1.348 \| 1.237 \| 0.932 \| <0.001 \| \|  \| R ILF AD x10^-5^[mm^2^/s] \| -0.069 \| -1.246 \| 1.108 \| 0.906 \| <0.001 \| \|  \| R SLFP FA x10^-2^ \| -0.109 \| -0.990 \| 0.771 \| 0.804 \| 0.001 \| \|  \| L SLFT FA x10^-2^ \| 0.264 \| -0.579 \| 1.107 \| 0.532 \| 0.008 \| \|  \| L pars opercularis thk[mm] x10 \| -1.860 \| -3.572 \| -0.147 \| **0.034** \| **0.079** \| \|  \| L isthmus cingulate thk[mm] x10 \| -0.555 \| -2.135 \| 1.026 \| 0.485 \| 0.009 \| \|  \| L insula thk[mm] x 10 \| -1.737 \| -3.424 \| -0.050 \| **0.044** \| **0.072** \| \|  \| L thalamus vol x 10^4^ \| -0.365 \| -1.035 \| 0.306 \| 0.280 \| 0.021 \| \|  \| R thalamus vol x 10^4^ \| -0.202 \| -0.893 \| 0.490 \| 0.562 \| 0.006 \| \|  \| WM hypointensity vol x10^4^ \| 0.124 \| -0.038 \| 0.286 \| 0.130 \| 0.041 \| \|  \| CC Central vol x 10^5^ \| -0.273 \| -0.534 \| -0.012 \| **0.041** \| **0.074** \| \|  \| NBV x 10^2^ \| -0.376 \| -1.120 \| 0.367 \| 0.315 \| 0.018 \| \| **CI** \| L CAB AD x 10^-5^[mm^2^/s] \| 0.341 \| -1.453 \| 2.135 \| 0.690 \| 0.012 \| \|  \| L CAB FA x10^-2^ \| 0.528 \| -1.830 \| 2.885 \| 0.639 \| 0.016 \| \|  \| L UNC AD x10^-5^[mm^2^/s] \| 0.291 \| -1.601 \| 2.183 \| 0.746 \| 0.008 \| \|  \| R ILF FA x10^-2^ \| -1.061 \| -4.170 \| 2.048 \| 0.476 \| 0.037 \| \|  \| R ILF AD x10^-5^[mm^2^/s] \| -0.910 \| -2.853 \| 1.033 \| 0.332 \| 0.067 \| \|  \| R UNC AD x10^-5^[mm^2^/s] \| 0.424 \| -2.061 \| 2.909 \| 0.720 \| 0.009 \| \|  \| R ILF FA x10^-2^ \| 2.750 \| -2.539 \| 8.038 \| 0.282 \| 0.088 \| \|  \| R ILF AD x10^-5^[mm^2^/s] \| 4.165 \| 0.448 \| 7.882 \| **0.031** \| **0.311** \| \|  \| R SLFP FA x10^-2^ \| -0.273 \| -2.488 \| 1.942 \| 0.794 \| 0.005 \| \|  \| L SLFT FA x10^-2^ \| -1.484 \| -5.610 \| 2.641 \| 0.451 \| 0.044 \| \|  \| L pars opercularis thk[mm] x10 \| 0.877 \| -6.392 \| 8.145 \| 0.800 \| 0.005 \| \|  \| L isthmus cingulate thk[mm] x10 \| -0.759 \| -7.707 \| 6.188 \| 0.818 \| 0.004 \| \|  \| L insula thk[mm] x10 \| -1.476 \| -8.443 \| 5.491 \| 0.656 \| 0.015 \| \|  \| L thalamus vol x 10^3^ \| -0.964 \| -2.591 \| 0.664 \| 0.225 \| 0.103 \| \|  \| R thalamus vol x 10^4^ \| -1.240 \| -3.204 \| 0.724 \| 0.197 \| 0.116 \| \|  \| WM hypointensity vol x10^4^ \| 0.171 \| -0.069 \| 0.411 \| 0.148 \| 0.143 \| \|  \| CC Central vol x10^5^ \| -0.129 \| -0.947 \| 0.690 \| 0.741 \| 0.008 \| \|  \| NBV x10^2^ \| -0.040 \| -2.373 \| 2.293 \| 0.971 \| 0.000 \| |
| --- | --- | --- | --- | --- | --- | --- | --- | --- | --- | --- | --- | --- | --- | --- | --- | --- | --- | --- | --- | --- | --- | --- | --- | --- | --- | --- | --- | --- | --- | --- | --- | --- | --- | --- | --- | --- | --- | --- | --- | --- | --- | --- | --- | --- | --- | --- | --- | --- | --- | --- | --- | --- | --- | --- | --- | --- | --- | --- | --- | --- | --- | --- | --- | --- | --- | --- | --- | --- | --- | --- | --- | --- | --- | --- | --- | --- | --- | --- | --- | --- | --- | --- | --- | --- | --- | --- | --- | --- | --- | --- | --- | --- | --- | --- | --- | --- | --- | --- | --- | --- | --- | --- | --- | --- | --- | --- | --- | --- | --- | --- | --- | --- | --- | --- | --- | --- | --- | --- | --- | --- | --- | --- | --- | --- | --- | --- | --- | --- | --- | --- | --- | --- | --- | --- | --- | --- | --- | --- | --- | --- | --- | --- | --- | --- | --- | --- | --- | --- | --- | --- | --- | --- | --- | --- | --- | --- | --- | --- | --- | --- | --- | --- | --- | --- | --- | --- | --- | --- | --- | --- | --- | --- | --- | --- | --- | --- | --- | --- | --- | --- | --- | --- | --- | --- | --- | --- | --- | --- | --- | --- | --- | --- | --- | --- | --- | --- | --- | --- | --- | --- | --- | --- | --- | --- | --- | --- | --- | --- | --- | --- | --- | --- | --- | --- | --- | --- | --- | --- | --- | --- | --- | --- | --- | --- | --- | --- | --- | --- | --- | --- | --- | --- | --- | --- | --- | --- | --- | --- | --- | --- | --- | --- | --- | --- | --- | --- | --- | --- | --- | --- | --- | --- | --- | --- | --- | --- | --- | --- | --- |
| \|  \| **CTT2** \| **Estimate**  **(Beta coefficient)** \| **Lower**  **95% CI** \| **Upper**  **95% CI** \| **P value** \| **R2** \| \| --- \| --- \| --- \| --- \| --- \| --- \| --- \| \| **CP** \| L CAB AD x10^-5^[mm^2^/s] \| -0.507 \| -1.147 \| 0.134 \| 0.119 \| 0.044 \| \|  \| L CAB FA x10^-2^ \| 0.290 \| -0.621 \| 1.200 \| 0.526 \| 0.007 \| \|  \| L UNC AD x10^-5^[mm^2^/s] \| 0.228 \| -0.914 \| 1.369 \| 0.691 \| 0.003 \| \|  \| R ILF FA x10^-2^ \| -0.312 \| -1.461 \| 0.838 \| 0.589 \| 0.005 \| \|  \| R ILF FA x10^-5^ \| -0.969 \| -1.690 \| -0.248 \| **0.009** \| **0.117** \| \|  \| R UNC AD x10^-5^[mm^2^/s] \| 0.075 \| -0.787 \| 0.936 \| 0.863 \| 0.001 \| \|  \| R ILF FA x10^-2^ \| 0.159 \| -1.648 \| 1.967 \| 0.860 \| 0.001 \| \|  \| R ILF AD x10^-5^[mm^2^/s] \| 0.637 \| -1.000 \| 2.274 \| 0.438 \| 0.012 \| \|  \| R SLFP FA x10^-2^ \| 0.139 \| -1.093 \| 1.372 \| 0.821 \| 0.001 \| \|  \| L SLFT FA x10^-2^ \| 0.266 \| -0.916 \| 1.447 \| 0.654 \| 0.004 \| \|  \| L pars opercularis thk[mm] x10^1^ \| -2.238 \| -4.772 \| 0.295 \| 0.082 \| 0.054 \| \|  \| L isthmus cingulate thk[mm] x10^1^ \| -3.128 \| -5.285 \| -0.970 \| **0.005** \| **0.133** \| \|  \| L insula thk[mm] x 10^1^ \| -2.639 \| -5.094 \| -0.184 \| **0.036** \| **0.078** \| \|  \| L thalamus x 10^4^ \| -1.145 \| -2.084 \| -0.206 \| **0.018** \| **0.098** \| \|  \| R thalamus x 10^4^ \| -0.584 \| -1.584 \| 0.417 \| 0.247 \| 0.024 \| \|  \| WM hypointensity vol x10^4^ \| 0.312 \| 0.086 \| 0.538 \| **0.008** \| **0.122** \| \|  \| CC Central vol x 10^5^ \| -0.492 \| -0.864 \| -0.119 \| **0.011** \| **0.113** \| \|  \| NBV x 10^2^ \| -1.157 \| -2.207 \| -0.108 \| **0.031** \| **0.082** \| \| **CI** \| L CAB AD x 10^-5^[mm^2^/s] \| 0.851 \| -1.664 \| 3.366 \| 0.480 \| 0.036 \| \|  \| L CAB FA x10^-2^ \| 1.067 \| -2.251 \| 4.385 \| 0.502 \| 0.033 \| \|  \| L UNC AD x10^-5^[mm^2^/s] \| 1.763 \| -0.737 \| 4.262 \| 0.153 \| 0.140 \| \|  \| R ILF FA x10^-2^ \| -4.599 \| -8.242 \| -0.956 \| **0.017** \| **0.344** \| \|  \| R ILF AD x10^-5^[mm^2^/s] \| -0.446 \| -3.291 \| 2.398 \| 0.742 \| 0.008 \| \|  \| R UNC AD x10^-5^[mm^2^/s] \| 2.702 \| -0.486 \| 5.890 \| 0.091 \| 0.191 \| \|  \| L pars opercularis thk[mm] x10^-1^ \| -8.195 \| -17.408 \| 1.018 \| 0.077 \| 0.206 \| \|  \| R ILF FA x10^-2^ \| -1.141 \| -8.973 \| 6.692 \| 0.758 \| 0.008 \| \|  \| R ILF AD x10^-5^ [mm^2^/s] \| 1.883 \| -4.371 \| 8.136 \| 0.527 \| 0.032 \| \|  \| R SLFP FA x10^-2^ \| -2.628 \| -5.359 \| 0.103 \| 0.058 \| 0.249 \| \|  \| L SLFT FA x10^-2^ \| -4.604 \| -9.921 \| 0.713 \| 0.084 \| 0.212 \| \|  \| L isthmus cingulate thk[mm] x 10^1^ \| -5.329 \| -14.727 \| 4.068 \| 0.244 \| 0.096 \| \|  \| L insula thk[mm] x10^1^ \| -9.549 \| -17.872 \| -1.226 \| **0.027** \| **0.302** \| \|  \| L thalamus vol x 10^3^ \| -2.808 \| -4.641 \| -0.976 \| **0.005** \| **0.436** \| \|  \| R thalamus vol x 10^4^ \| -3.770 \| -5.800 \| -1.739 \| **0.001** \| **0.531** \| \|  \| WM hypointensity vol x 10^4^ \| 0.348 \| 0.038 \| 0.657 \| **0.030** \| **0.293** \| \|  \| CC Central vol x 10^5^ \| -1.097 \| -2.079 \| -0.115 \| **0.031** \| **0.291** \| \|  \| NBV x10^2^ \| -3.393 \| -6.073 \| -0.713 \| **0.017** \| **0.345** \| |
| **Abbreviations**: AD, axial diffusivity; ATR, anterior thalamic radiation; CAB, cingulum–angular (infracallosal) bundle; CC, corpus callosum; CCG, cingulum–cingulate gyrus (supracallosal) bundle; CST, corticospinal tract; CTT, Color Trails Test; EDSS, Expanded Disability Status Score; e-TIV, estimated total intracranial volume; FA, fractional anisotropy; FMAJ, corpus callosum–forceps major; FMIN, corpus callosum–forceps minor; ILF, inferior longitudinal fasciculus; IPS, information processing speed; L, left hemisphere; mm, millimetres; NBV, normalized brain volume; PASAT, Paced Auditory Serial Additive Test; R, right hemisphere; s, second; SDMT, Symbol Digit Modalities Test; SLFP, superior longitudinal fasciculus–parietal bundle; SLFT, superior longitudinal fasciculus–temporal bundle; thk, thickness; UNC, uncinate fasciculus; vol, volume normalized to estimated total intracranial volume; WMH, white matter hypointensities. |
