# Supplementary material for: The association between white matter tract structural connectivity and information processing speed in relapsing-remitting multiple sclerosis
Source: Neurol Sci. 2023 Apr 27;44(9):3221–32. doi: 10.1007/s10072-023-06817-6 (PMC10415523; doi:10.1007/s10072-023-06817-6)
Supplement: Supplementary file 3 — Table 3. Multiple regression models with MRI measures, IPS preserved and IPS impaired subgroups, and interaction between them for cognitive tests [file 10072_2023_6817_MOESM3_ESM.docx]

**Table 3.** Multiple regression models with MRI measures, IPS preserved and IPS impaired subgroups, and interaction between them for cognitive tests

|  | Estimate  (Beta coefficient) | | | Lower  95% CI | | Upper  95% CI | | p-value | | | | R^2^ | |  |
| --- | --- | --- | --- | --- | --- | --- | --- | --- | --- | --- | --- | --- | --- | --- |
| **PASAT z-cognitive** | | | | | | | | | | | | | |  |
| CAB AD x10^-5^[mm^2^/s] | 0.003 | | -0.009 | | | 0.014 | | | 0.639 | | | 0.858 | |  |
| Subgroup IPS impaired (Ref. IPS preserved) | 0.535 | | -2.285 | | | 3.354 | | | 0.706 | | |  | |  |
| Interaction (CAB AD x10^-5^[mm^2^/s], Subgroup) | -0.004 | | -0.030 | | | 0.021 | | | 0.732 | | |  | |  |
| Age | 0.077 | | 0.069 | | | 0.085 | | | **< 0.001** | | |  | |  |
| Sex (Ref. Female) | 0.052 | | -0.101 | | | 0.205 | | | 0.497 | | |  | |  |
| Education (years) | -0.022 | | -0.052 | | | 0.007 | | | 0.137 | | |  | |  |
| EDSS | -0.011 | | -0.093 | | | 0.072 | | | 0.800 | | |  | |  |
| **SDMT** |  | |  | | |  | | |  | | |  | |  |
| CAB AD x10^-5^[mm^2^/s] | 0.155 | | -0.211 | | | 0.522 | | | 0.401 | | | 0.384 | |  |
| Subgroup IPS impaired (Ref. IPS preserved) | -2.700 | | -93.106 | | | 87.705 | | | 0.953 | | |  | |  |
| Interaction (CAB AD x10^-5^[mm^2^/s], Subgroup) | -0.075 | | -0.903 | | | 0.753 | | | 0.857 | | |  | |  |
| Age | -0.053 | | -0.306 | | | 0.200 | | | 0.678 | | |  | |  |
| Sex (Ref. Female) | -4.637 | | -9.534 | | | 0.259 | | | 0.063 | | |  | |  |
| Education (years) | 1.095 | | 0.148 | | | 2.041 | | | **0.024** | | |  | |  |
| EDSS | -4.018 | | -6.670 | | | -1.366 | | | **0.004** | | |  | |  |
| **CTT-1** |  | |  | | |  | | |  | | |  | |  |
| CAB AD x10^-5^[mm^2^/s] | -0.082 | | -0.619 | | | 0.456 | | | 0.763 | | | 0.316 | |  |
| Subgroup IPS impaired (Ref. IPS preserved) | -43.201 | | -175.662 | | | 89.259 | | | 0.517 | | |  | |  |
| Interaction (CAB AD x10^-5^[mm^2^/s], Subgroup) | 0.585 | | -0.628 | | | 1.797 | | | 0.339 | | |  | |  |
| Age | 0.181 | | -0.190 | | | 0.552 | | | 0.334 | | |  | |  |
| Sex (Ref. Female) | 7.518 | | 0.343 | | | 14.693 | | | **0.040** | | |  | |  |
| Education (years) | 0.976 | | -0.411 | | | 2.363 | | | 0.165 | | |  | |  |
| EDSS | 2.108 | | -1.778 | | | 5.994 | | | 0.283 | | |  | |  |
| **CTT-2** |  | |  | | |  | | |  | | |  | |  |
| CAB AD x10^-5^[mm^2^/s] | -0.360 | | -1.091 | | | 0.371 | | | 0.329 | | | 0.490 | |  |
| Subgroup CI (Ref. CP) | -123.995 | | -304.291 | | | 56.301 | | | 0.174 | | |  | |  |
| Interaction (CAB AD x10^-5^[mm^2^/s], Subgroup) | 1.471 | | -0.179 | | | 3.122 | | | 0.080 | | |  | |  |
| Age | 0.441 | | -0.064 | | | 0.946 | | | 0.086 | | |  | |  |
| Sex (Ref. Female) | 8.788 | | -0.978 | | | 18.554 | | | 0.077 | | |  | |  |
| Education (years) | 1.354 | | -0.534 | | | 3.243 | | | 0.157 | | |  | |  |
| EDSS | 6.629 | | 1.340 | | | 11.919 | | | **0.015** | | |  | |  |
| **PASAT z-cognitive** |  | |  | | |  | | |  | | |  | |  |
| CAB FA x10^-2^ | -0.012 | | -0.028 | | | 0.005 | | | 0.157 | | | 0.862 | |  |
| Subgroup IPS impaired (Ref. IPS preserved) | -0.227 | | -1.278 | | | 0.825 | | | 0.668 | | |  | |  |
| Interaction (CAB FAx10^-2^, Subgroup) | 0.009 | | -0.026 | | | 0.044 | | | 0.607 | | |  | |  |
| Age | 0.077 | | 0.070 | | | 0.085 | | | **< 0.001** | | |  | |  |
| Sex (Ref. Female) | 0.062 | | -0.092 | | | 0.215 | | | 0.424 | | |  | |  |
| Education | -0.026 | | -0.056 | | | 0.003 | | | 0.079 | | |  | |  |
| EDSS | -0.028 | | -0.110 | | | 0.055 | | | 0.506 | | |  | |  |
| **SDMT** |  | |  | | |  | | |  | | |  | |  |
| CAB FA x10^-2^ | -0.266 | | -0.789 | | | 0.256 | | | 0.313 | | | 0.390 | |  |
| Subgroup IPS impaired (Ref. IPS preserved) | -27.388 | | -61.415 | | | 6.640 | | | 0.113 | | |  | |  |
| Interaction (CAB FA x10^-2^, Subgroup) | 0.553 | | -0.571 | | | 1.678 | | | 0.329 | | |  | |  |
| Age | -0.029 | | -0.281 | | | 0.222 | | | 0.817 | | |  | |  |
| Sex (Ref. Female) | -4.831 | | -9.791 | | | 0.130 | | | 0.056 | | |  | |  |
| Education | 0.949 | | -0.008 | | | 1.906 | | | 0.052 | | |  | |  |
| EDSS | -4.498 | | -7.173 | | | -1.823 | | | **0.001** | | |  | |  |
| **CTT-1** |  | |  | | |  | | |  | | |  | |  |
| CAB FA x10^-2^ | 0.084 | | -0.690 | | | 0.857 | | | 0.830 | | | 0.308 | |  |
| Subgroup IPS impaired (Ref. IPS preserved) | 14.980 | | -35.396 | | | 65.356 | | | 0.555 | | |  | |  |
| Interaction (CAB FA x10^-2^, Subgroup) | 0.193 | | -1.473 | | | 1.858 | | | 0.818 | | |  | |  |
| Age | 0.165 | | -0.207 | | | 0.537 | | | 0.378 | | |  | |  |
| Sex (Ref. Female) | 7.039 | | -0.305 | | | 14.383 | | | 0.060 | | |  | |  |
| Education | 0.944 | | -0.473 | | | 2.361 | | | 0.188 | | |  | |  |
| EDSS | 2.307 | | -1.653 | | | 6.267 | | | 0.249 | | |  | |  |
| **CTT-2** |  | |  | | |  | | |  | | |  | |  |
| CAB FA x10^-2^ | 0.651 | | -0.402 | | | 1.704 | | | 0.221 | | | 0.484 | |  |
| Subgroup IPS impaired (Ref. IPS preserved) | 28.284 | | -40.292 | | | 96.859 | | | 0.413 | | |  | |  |
| Interaction (CAB FA x10^-2^, Subgroup) | 0.295 | | -1.972 | | | 2.562 | | | 0.796 | | |  | |  |
| Age | 0.384 | | -0.123 | | | 0.890 | | | 0.135 | | |  | |  |
| Sex (Ref. Female) | 7.191 | | -2.806 | | | 17.189 | | | 0.156 | | |  | |  |
| Education | 1.454 | | -0.475 | | | 3.384 | | | 0.137 | | |  | |  |
| EDSS | 7.875 | | 2.484 | | | 13.266 | | | **0.005** | | |  | |  |
| **PASAT z-cognitive** |  | |  | | |  | | |  | | |  | |  |
| LH UNC AD x10^-5^[mm^2^/s] | 0.005 | | -0.015 | | | 0.026 | | | 0.599 | | | 0.858 | |  |
| Subgroup IPS impaired (Ref. IPS preseved) | 0.748 | | -2.857 | | | 4.354 | | | 0.680 | | |  | |  |
| Interaction (LH UNC AD x10^-5^[mm^2^/s], Subgroup) | -0.006 | | -0.038 | | | 0.025 | | | 0.699 | | |  | |  |
| Age | 0.077 | | 0.069 | | | 0.085 | | | **< 0.001** | | |  | |  |
| Sex (Ref. Female) | 0.049 | | -0.103 | | | 0.202 | | | 0.522 | | |  | |  |
| Education | -0.020 | | -0.051 | | | 0.010 | | | 0.187 | | |  | |  |
| EDSS | -0.013 | | -0.095 | | | 0.069 | | | 0.756 | | |  | |  |
| **SDMT** |  | |  | | |  | | |  | | |  | |  |
| LH UNC AD x10^-5^[mm^2^/s] | -0.583 | | -1.205 | | | 0.039 | | | 0.066 | | | 0.438 | |  |
| Subgroup IPS impaired (Ref. IPS preserved) | 5.185 | | -105.303 | | | 115.672 | | | 0.926 | | |  | |  |
| Interaction (L UNC ADx10^-5^[mm^2^/s], Subgroup) | -0.132 | | -1.100 | | | 0.837 | | | 0.787 | | |  | |  |
| Age | -0.065 | | -0.306 | | | 0.176 | | | 0.591 | | |  | |  |
| Sex (Ref. Female) | -4.407 | | -9.084 | | | 0.271 | | | 0.064 | | |  | |  |
| Education (years) | 0.782 | | -0.153 | | | 1.717 | | | 0.100 | | |  | |  |
| EDSS | -3.943 | | -6.464 | | | -1.421 | | | **0.003** | | |  | |  |
| **CTT-1** |  | |  | | |  | | |  | | |  | |  |
| L UNC AD x10^-5^[mm^2^/s] | -0.203 | | -1.161 | | | 0.754 | | | 0.673 | | | 0.309 | |  |
| Subgroup IPS impaired CI (Ref. IPS preserved) | -28.074 | | -198.277 | | | 142.129 | | | 0.743 | | |  | |  |
| Interaction (L UNC AD x10^-5^[mm^2^/s], Subgroup) | 0.428 | | -1.065 | | | 1.920 | | | 0.569 | | |  | |  |
| Age | 0.162 | | -0.209 | | | 0.533 | | | 0.387 | | |  | |  |
| Sex (Ref. Female) | 7.429 | | 0.224 | | | 14.634 | | | 0.043 | | |  | |  |
| Education (years) | 0.864 | | -0.577 | | | 2.305 | | | 0.235 | | |  | |  |
| EDSS | 2.054 | | -1.830 | | | 5.939 | | | 0.295 | | |  | |  |
| **CTT-2** |  | |  | | |  | | |  | | |  | |  |
| L UNC AD x10^-5^[mm^2^/s] | 0.453 | | -0.833 | | | 1.739 | | | 0.484 | | | 0.499 | |  |
| Subgroup IPS impaired (Ref. IPS preserved) | -90.863 | | -319.378 | | | 137.652 | | | 0.430 | | |  | |  |
| Interaction (L UNC AD x10^-5^[mm^2^/s], Subgroup) | 1.106 | | -0.898 | | | 3.110 | | | 0.275 | | |  | |  |
| Age | 0.419 | | -0.079 | | | 0.917 | | | 0.098 | | |  | |  |
| Sex (Ref. Female) | 8.160 | | -1.514 | | | 17.834 | | | 0.097 | | |  | |  |
| Education (years) | 1.564 | | -0.370 | | | 3.499 | | | 0.111 | | |  | |  |
| EDSS | 6.331 | | 1.116 | | | 11.546 | | | **0.018** | | |  | |  |
| **PASAT z-cognitive** |  | |  | | |  | | |  | | |  | |  |
| R ILF FA x10^-2^ | -0.017 | | -0.037 | | | 0.002 | | | 0.079 | | | 0.864 | |  |
| Subgroup IPS impaired (Ref. IPS preserved) | -0.410 | | -2.091 | | | 1.270 | | | 0.627 | | |  | |  |
| Interaction (R ILF FA x10^-2^, Subgroup) | 0.012 | | -0.032 | | | 0.056 | | | 0.600 | | |  | |  |
| Age | 0.076 | | 0.068 | | | 0.084 | | | **< 0.001** | | |  | |  |
| Sex (Ref. Female) | 0.051 | | -0.098 | | | 0.200 | | | 0.499 | | |  | |  |
| Education (years) | -0.025 | | -0.054 | | | 0.004 | | | 0.085 | | |  | |  |
| EDSS | -0.023 | | -0.103 | | | 0.058 | | | 0.576 | | |  | |  |
| **SDMT** |  | |  | | |  | | |  | | |  | |  |
| R ILF FA x10^-2^ | 0.154 | | -0.481 | | | 0.788 | | | 0.631 | | | 0.396 | |  |
| Subgroup IPS impaired (Ref. IPS preserved) | -38.008 | | -92.567 | | | 16.551 | | | 0.169 | | |  | |  |
| Interaction (R ILF FA x10^-2^, Subgroup) | 0.731 | | -0.701 | | | 2.164 | | | 0.312 | | |  | |  |
| Age | -0.026 | | -0.277 | | | 0.225 | | | 0.839 | | |  | |  |
| Sex (Ref. Female) | -4.961 | | -9.812 | | | -0.110 | | | **0.045** | | |  | |  |
| Education (years) | 1.127 | | 0.188 | | | 2.066 | | | 0.019 | | |  | |  |
| EDSS | -4.001 | | -6.614 | | | -1.389 | | | **0.003** | | |  | |  |
| **CTT-1** |  | |  | | |  | | |  | | |  | |  |
| R ILF FA x10^-2^ | 0.079 | | -0.859 | | | 1.016 | | | 0.867 | | | 0.318 | |  |
| Subgroup CI (Ref. IPS preserved) | 61.928 | | -18.669 | | | 142.525 | | | 0.130 | | |  | |  |
| Interaction (R ILF FA x10^-2^, Subgroup) | -1.095 | | -3.211 | | | 1.021 | | | 0.305 | | |  | |  |
| Age | 0.154 | | -0.216 | | | 0.525 | | | 0.409 | | |  | |  |
| Sex (Ref. Female) | 7.641 | | 0.475 | | | 14.806 | | | 0.037 | | |  | |  |
| Education (years) | 0.894 | | -0.493 | | | 2.281 | | | 0.203 | | |  | |  |
| EDSS | 2.050 | | -1.809 | | | 5.909 | | | 0.293 | | |  | |  |
| **CTT-2** |  | |  | | |  | | |  | | |  | |  |
| R ILF FA x10^-2^ | 0.029 | | -1.169 | | | 1.228 | | | 0.961 | | | **0.552** | |  |
| Subgroup IPS impaired (Ref. IPS preserved) | 201.126 | | 98.089 | | | 304.164 | | | **< 0.001** | | |  | |  |
| Interaction (R ILF FA x 10^-2^, Subgroup) | -4.378 | | -7.083 | | | -1.673 | | | **0.002** | | |  | |  |
| Age | 0.337 | | -0.137 | | | 0.811 | | | 0.160 | | |  | |  |
| Sex (Ref. Female) | 9.696 | | 0.535 | | | 18.857 | | | 0.038 | | |  | |  |
| Education (years) | 1.077 | | -0.696 | | | 2.850 | | | 0.230 | | |  | |  |
| EDSS | 6.315 | | 1.381 | | | 11.248 | | | **0.013** | | |  | |  |
| **z PASAT z-cognitive** |  | |  | | |  | | |  | | |  | |  |
| R ILF AD x10^-5^[mm^2^/s] | -0.003 | | -0.016 | | | 0.011 | | | 0.672 | | | 0.858 | |  |
| Subgroup IPS impaired (Ref. IPS preserved) | -0.005 | | -3.529 | | | 3.519 | | | 0.998 | | |  | |  |
| Interaction (R ILF AD x10^-5^[mm^2^/s], Subgroup) | 0.001 | | -0.029 | | | 0.030 | | | 0.973 | | |  | |  |
| Age | 0.077 | | 0.069 | | | 0.085 | | | **< 0.001** | | |  | |  |
| Sex (Ref. Female) | 0.049 | | -0.105 | | | 0.204 | | | 0.524 | | |  | |  |
| Education (years) | -0.023 | | -0.053 | | | 0.007 | | | 0.125 | | |  | |  |
| EDSS | -0.018 | | -0.101 | | | 0.066 | | | 0.676 | | |  | |  |
| **SDMT** |  | |  | | |  | | |  | | |  | |  |
| R ILF AD x10^-5^[mm^2^/s] | 0.228 | | -0.184 | | | 0.639 | | | 0.274 | | | **0.439** | |  |
| Subgroup IPS impaired (Ref. IPS preserved) | 133.466 | | 25.631 | | | 241.301 | | | **0.016** | | |  | |  |
| Interaction (R ILF AD x10^-5^[mm^2^/s], Subgroup) | -1.212 | | -2.117 | | | -0.306 | | | **0.009** | | |  | |  |
| Age | -0.055 | | -0.295 | | | 0.186 | | | 0.652 | | |  | |  |
| Sex (Ref. Female) | -5.599 | | -10.317 | | | -0.881 | | | **0.021** | | |  | |  |
| Education (years) | 0.884 | | -0.031 | | | 1.799 | | | 0.058 | | |  | |  |
| EDSS | -3.849 | | -6.408 | | | -1.291 | | | **0.004** | | |  | |  |
| **CTT-1** |  | |  | | |  | | |  | | |  | |  |
| R ILF AD x10^-5^[mm^2^/s] | -0.096 | | -0.727 | | | 0.535 | | | 0.762 | | | 0.317 | |  |
| Subgroup IPS impaired (Ref. IPS preserved) | 83.819 | | -81.485 | | | 249.123 | | | 0.315 | | |  | |  |
| Interaction (R ILF AD x10^-5^[mm^2^/s], Subgroup) | -0.529 | | -1.917 | | | 0.858 | | | 0.449 | | |  | |  |
| Age | 0.149 | | -0.220 | | | 0.518 | | | 0.424 | | |  | |  |
| Sex (Ref. Female) | 6.792 | | -0.440 | | | 14.024 | | | 0.065 | | |  | |  |
| Education (years) | 0.795 | | -0.607 | | | 2.197 | | | 0.262 | | |  | |  |
| EDSS | 2.079 | | -1.842 | | | 6.001 | | | 0.294 | | |  | |  |
| **CTT-2** |  | |  | | |  | | |  | | |  | |  |
| R ILF AD x10^-5^[mm^2^/s] | -0.687 | | -1.551 | | | 0.178 | | | 0.118 | | | 0.485 | |  |
| Subgroup IPS impaired (Ref. IPS preserved) | -35.699 | | -262.134 | | | 190.736 | | | 0.754 | | |  | |  |
| Interaction (R ILF AD x10^5^[mm^2^/s], Subgroup) | 0.611 | | -1.290 | | | 2.512 | | | 0.523 | | |  | |  |
| Age | 0.367 | | -0.139 | | | 0.872 | | | 0.152 | | |  | |  |
| Sex (Ref. Female) | 8.396 | | -1.511 | | | 18.302 | | | 0.095 | | |  | |  |
| Education (years) | 1.200 | | -0.720 | | | 3.121 | | | 0.216 | | |  | |  |
| EDSS | 6.065 | | 0.693 | | | 11.437 | | | **0.028** | | |  | |  |
| **PASAT z-cognitive** |  | |  | | |  | | |  | | |  | |  |
| R UNC AD x10^-5^[mm^2^/s] | 0.006 | | -0.009 | | | 0.021 | | | 0.402 | | | 0.859 | |  |
| Subgroup IPS impaired (Ref. IPS preserved) | 0.343 | | -3.792 | | | 4.478 | | | 0.869 | | |  | |  |
| Interaction (R UNC AD x10^-5^[mm^2^/s], Subgroup) | -0.003 | | -0.039 | | | 0.034 | | | 0.881 | | |  | |  |
| Age | 0.077 | | 0.069 | | | 0.085 | | | **< 0.001** | | |  | |  |
| Sex (Ref. Female) | 0.050 | | -0.107 | | | 0.206 | | | 0.530 | | |  | |  |
| Education (years) | -0.023 | | -0.052 | | | 0.006 | | | 0.122 | | |  | |  |
| EDSS | -0.010 | | -0.093 | | | 0.072 | | | 0.804 | | |  | |  |
| **SDMT** |  | |  | | |  | | |  | | |  | |  |
| R UNC AD x10^-5^[mm^2^/s] | -0.340 | | -0.781 | | | 0.102 | | | 0.129 | | | **0.477** | |  |
| Subgroup IPS impaired (Ref. IPS preserved) | 124.929 | | 2.317 | | | 247.541 | | | **0.046** | | |  | |  |
| Interaction (R UNC AD x10^-5^[mm^2^/s], Subgroup) | -1.187 | | -2.273 | | | -0.100 | | | **0.033** | | |  | |  |
| Age | -0.031 | | -0.263 | | | 0.202 | | | 0.794 | | |  | |  |
| Sex (Ref. Female) | -6.094 | | -10.743 | | | -1.444 | | | **0.011** | | |  | |  |
| Education (years) | 1.111 | | 0.243 | | | 1.979 | | | **0.013** | | |  | |  |
| EDSS | -3.925 | | -6.381 | | | -1.470 | | | **0.002** | | |  | |  |
| **CTT-1** |  | |  | | |  | | |  | | |  | |  |
| R UNC AD x10^-5^[mm^2^/s] | 0.086 | | -0.617 | | | 0.788 | | | 0.808 | | | 0.315 | |  |
| Subgroup IPS impaired (Ref. IPS preserved) | -46.814 | | -241.979 | | | 148.351 | | | 0.634 | | |  | |  |
| Interaction (R UNC AD x10^-5^[mm^2^/s], Subgroup) | 0.592 | | -1.138 | | | 2.322 | | | 0.497 | | |  | |  |
| Age | 0.161 | | -0.209 | | | 0.531 | | | 0.389 | | |  | |  |
| Sex (Ref. Female) | 8.014 | | 0.614 | | | 15.414 | | | **0.034** | | |  | |  |
| Education (years) | 0.915 | | -0.466 | | | 2.297 | | | 0.190 | | |  | |  |
| EDSS | 2.002 | | -1.907 | | | 5.911 | | | 0.310 | | |  | |  |
| **CTT-2** |  | |  | | |  | | |  | | |  | |  |
| R UNC AD x10^-5^[mm^2^/s] | 0.054 | | -0.869 | | | 0.977 | | | 0.908 | | | **0.524** | |  |
| Subgroup IPS impaired (Ref. IPS preserved) | -287.102 | | -543.466 | | | -30.738 | | | **0.029** | | |  | |  |
| Interaction (R UNC AD x10^-5^[mm^2^/s], Subgroup) | 2.853 | | 0.580 | | | 5.125 | | | **0.015** | | |  | |  |
| Age | 0.395 | | -0.092 | | | 0.881 | | | 0.110 | | |  | |  |
| Sex (Ref. Female) | 11.641 | | 1.920 | | | 21.361 | | | **0.020** | | |  | |  |
| Education (years) | 1.232 | | -0.582 | | | 3.047 | | | 0.180 | | |  | |  |
| EDSS | 6.015 | | 0.881 | | | 11.150 | | | **0.022** | | |  | |  |
| **PASAT z-cognitive** |  | |  | | |  | | |  | | |  | |  |
| SLFP FA x10^-2^ | 0.001 | | -0.029 | | | 0.031 | | | 0.935 | | | 0.858 | |  |
| Subgroup CI (Ref. IPS preserved) | 0.288 | | -1.956 | | | 2.531 | | | 0.799 | | |  | |  |
| Interaction (SLFP FA x10^-2^, Subgroup) | -0.006 | | -0.067 | | | 0.055 | | | 0.834 | | |  | |  |
| Age | 0.077 | | 0.069 | | | 0.085 | | | **< 0.001** | | |  | |  |
| Sex (Ref. Female) | 0.053 | | -0.102 | | | 0.207 | | | 0.497 | | |  | |  |
| Education (years) | -0.022 | | -0.052 | | | 0.007 | | | 0.138 | | |  | |  |
| EDSS | -0.014 | | -0.096 | | | 0.068 | | | 0.735 | | |  | |  |
| **SDMT** |  | |  | | |  | | |  | | |  | |  |
| SLFP FA x10^-2^ | 0.404 | | -0.536 | | | 1.345 | | | 0.394 | | | 0.403 | |  |
| Subgroup IPS impaired (Ref. IPS preserved) | -40.934 | | -111.644 | | | 29.776 | | | 0.252 | | |  | |  |
| Interaction (SLFP FA x10^-2^, Subgroup) | 0.838 | | -1.085 | | | 2.761 | | | 0.387 | | |  | |  |
| Age | -0.025 | | -0.274 | | | 0.224 | | | 0.840 | | |  | |  |
| Sex (Ref. Female) | -5.351 | | -10.218 | | | -0.483 | | | **0.032** | | |  | |  |
| Education (years) | 1.160 | | 0.219 | | | 2.100 | | | **0.016** | | |  | |  |
| EDSS | -4.155 | | -6.729 | | | -1.581 | | | **0.002** | | |  | |  |
| **CTT-1** |  | |  | | |  | | |  | | |  | |  |
| SLFP FA x10^-2^ | -0.445 | | -1.832 | | | 0.942 | | | 0.524 | | | 0.328 | |  |
| Subgroup IPS impaired (Ref. CP) | -55.366 | | -159.641 | | | 48.909 | | | 0.293 | | |  | |  |
| Interaction (SLFP FA x10^-2^, Subgroup) | 2.073 | | -0.763 | | | 4.909 | | | 0.149 | | |  | |  |
| Age | 0.157 | | -0.210 | | | 0.525 | | | 0.396 | | |  | |  |
| Sex (Ref. Female) | 7.061 | | -0.117 | | | 14.239 | | | 0.054 | | |  | |  |
| Education (years) | 0.904 | | -0.483 | | | 2.291 | | | 0.198 | | |  | |  |
| EDSS | 2.220 | | -1.576 | | | 6.016 | | | 0.247 | | |  | |  |
| **CTT-2** |  | |  | | |  | | |  | | |  | |  |
| SLFP FA x10^-2^ | -0.520 | | -2.465 | | | 1.425 | | | 0.595 | | | 0.468 | |  |
| Subgroup IPS impaired (Ref. IPS preserved) | 42.480 | | -103.743 | | | 188.702 | | | 0.564 | | |  | |  |
| Interaction (SLFP FA x10^-2^, Subgroup) | -0.180 | | -4.157 | | | 3.798 | | | 0.928 | | |  | |  |
| Age | 0.369 | | -0.146 | | | 0.885 | | | 0.157 | | |  | |  |
| Sex (Ref. Female) | 8.995 | | -1.070 | | | 19.060 | | | 0.079 | | |  | |  |
| Education (years) | 1.183 | | -0.762 | | | 3.128 | | | 0.229 | | |  | |  |
| EDSS | 6.962 | | 1.640 | | | 12.285 | | | 0.011 | | |  | |  |
| **PASAT z-cognitive** |  | |  | | |  | | |  | | |  | |  |
| SLFT FA x10^-2^ | 0.013 | | -0.017 | | | 0.043 | | | 0.390 | | | **0.859** | |  |
| Subgroup IPS impaired (Ref. IPS preserved) | 0.533 | | -1.819 | | | 2.885 | | | 0.652 | | |  | |  |
| Interaction (SLFP FA x10^-2^, Subgroup) | -0.012 | | -0.071 | | | 0.047 | | | 0.687 | | |  | |  |
| Age | 0.078 | | 0.070 | | | 0.086 | | | **< 0.001** | | |  | |  |
| Sex (Ref. Female) | 0.033 | | -0.127 | | | 0.192 | | | 0.684 | | |  | |  |
| Education (years) | -0.017 | | -0.049 | | | 0.014 | | | 0.276 | | |  | |  |
| EDSS | -0.013 | | -0.094 | | | 0.069 | | | 0.759 | | |  | |  |
| **SDMT** |  | |  | | |  | | |  | | |  | |  |
| SLFT FA x10^-2^ | 0.230 | | -0.735 | | | 1.195 | | | 0.636 | | | 0.384 | |  |
| Subgroup IPS impaired (Ref. IPS preserved) | -23.913 | | -99.639 | | | 51.813 | | | 0.530 | | |  | |  |
| Interaction (SLFP FA x10^-2^, Subgroup) | 0.334 | | -1.554 | | | 2.221 | | | 0.725 | | |  | |  |
| Age | -0.026 | | -0.287 | | | 0.235 | | | 0.842 | | |  | |  |
| Sex (Ref. Female) | -5.336 | | -10.473 | | | -0.200 | | | **0.042** | | |  | |  |
| Education (years) | 1.112 | | 0.090 | | | 2.134 | | | 0.033 | | |  | |  |
| EDSS | -4.237 | | -6.857 | | | -1.618 | | | 0.002 | | |  | |  |
| **CTT-1** |  | |  | | |  | | |  | | |  | |  |
| SLFT FA x10^-2^ | -0.191 | | -1.545 | | | 1.162 | | | 0.779 | | | **0.373** | |  |
| Subgroup CI (Ref. IPS preserved) | -106.328 | | -212.512 | | | -0.144 | | | **0.050** | | |  | |  |
| Interaction (SLFP FA x10^2^, Subgroup) | 3.180 | | 0.533 | | | 5.827 | | | **0.019** | | |  | |  |
| Age | 0.151 | | -0.215 | | | 0.517 | | | 0.412 | | |  | |  |
| Sex (Ref. Female) | 6.192 | | -1.010 | | | 13.395 | | | 0.091 | | |  | |  |
| Education (years) | 0.642 | | -0.791 | | | 2.076 | | | 0.374 | | |  | |  |
| EDSS | 1.919 | | -1.754 | | | 5.591 | | | 0.301 | | |  | |  |
| **CTT-2** |  | |  | | |  | | |  | | |  | |  |
| SLFT FA x10^-2^ | -0.114 | | -2.081 | | | 1.853 | | | 0.908 | | | 0.467 | |  |
| Subgroup IPS impaired (Ref. IPS preserved) | -1.288 | | -155.624 | | | 153.047 | | | 0.987 | | |  | |  |
| Interaction (SLFP FA x10^-2^, Subgroup) | 0.943 | | -2.905 | | | 4.790 | | | 0.626 | | |  | |  |
| Age | 0.381 | | -0.151 | | | 0.913 | | | 0.158 | | |  | |  |
| Sex (Ref. Female) | 8.263 | | -2.205 | | | 18.732 | | | 0.120 | | |  | |  |
| Education (years) | 1.181 | | -0.902 | | | 3.264 | | | 0.262 | | |  | |  |
| EDSS | 6.929 | | 1.591 | | | 12.267 | | | 0.012 | | |  | |  |
| **PASAT z-cognitive** |  | |  | | |  | | |  | | |  | |  |
| FMAJ FA x10^-2^ | -0.007 | | -0.028 | | | 0.014 | | | 0.497 | | | 0.859 | |  |
| Subgroup IPS impaired (Ref. IPS preserved) | -0.161 | | -1.864 | | | 1.541 | | | 0.850 | | |  | |  |
| Interaction (FMAJ FA x10^-2^, Subgroup) | 0.004 | | -0.029 | | | 0.036 | | | 0.818 | | |  | |  |
| Age | 0.077 | | 0.069 | | | 0.085 | | | **< 0.001** | | |  | |  |
| Sex (Ref. Female) | 0.063 | | -0.092 | | | 0.218 | | | 0.422 | | |  | |  |
| Education (years) | -0.025 | | -0.055 | | | 0.005 | | | 0.104 | | |  | |  |
| EDSS | -0.012 | | -0.093 | | | 0.070 | | | 0.774 | | |  | |  |
| **SDMT** |  | |  | | |  | | |  | | |  | |  |
| FMAJ FA x10^-2^ | 0.288 | | -0.350 | | | 0.927 | | | 0.370 | | | 0.437 | |  |
| Subgroup IPS impaired (Ref. IPS preserved) | -44.872 | | -97.155 | | | 7.410 | | | 0.091 | | |  | |  |
| Interaction (FMAJ FA x10^-2^, Subgroup) | 0.696 | | -0.309 | | | 1.701 | | | 0.172 | | |  | |  |
| Age | -0.016 | | -0.257 | | | 0.225 | | | 0.896 | | |  | |  |
| Sex (Ref. Female) | -5.349 | | -10.117 | | | -0.582 | | | **0.028** | | |  | |  |
| Education (years) | 1.235 | | 0.316 | | | 2.153 | | | **0.009** | | |  | |  |
| EDSS | -4.210 | | -6.718 | | | -1.702 | | | **0.001** | | |  | |  |
| **CTT-1** |  | |  | | |  | | |  | | |  | |  |
| FMAJ FA x10^-2^ | -0.195 | | -1.178 | | | 0.789 | | | 0.694 | | | 0.308 | |  |
| Subgroup IPS impaired (Ref. IPS preserved) | 18.058 | | -62.509 | | | 98.624 | | | 0.656 | | |  | |  |
| Interaction (FMAJ FA x10^-2^, Subgroup) | 0.038 | | -1.511 | | | 1.587 | | | 0.961 | | |  | |  |
| Age | 0.157 | | -0.215 | | | 0.529 | | | 0.402 | | |  | |  |
| Sex (Ref. Female) | 7.668 | | 0.321 | | | 15.015 | | | 0.041 | | |  | |  |
| Education | 0.867 | | -0.549 | | | 2.282 | | | 0.226 | | |  | |  |
| EDSS | 2.226 | | -1.638 | | | 6.091 | | | 0.254 | | |  | |  |
| **CTT-2** |  | |  | | |  | | |  | | |  | |  |
| FMAJ FA x10^-2^ | 0.049 | | -1.246 | | | 1.344 | | | 0.940 | | | **0.518** | |  |
| Subgroup CI (Ref. CP) | 146.881 | | 40.833 | | | 252.929 | | | **0.007** | | |  | |  |
| Interaction (FMAJ FA x10^-2^, Subgroup) | -2.187 | | -4.226 | | | -0.149 | | | **0.036** | | |  | |  |
| Age | 0.337 | | -0.152 | | | 0.826 | | | 0.174 | | |  | |  |
| Sex (Ref. Female) | 8.823 | | -0.848 | | | 18.494 | | | 0.073 | | |  | |  |
| Education (years) | 1.083 | | -0.780 | | | 2.946 | | | 0.250 | | |  | |  |
| EDSS | 6.778 | | 1.692 | | | 11.865 | | | **0.010** | | |  | |  |
| **PASAT z-cognitive** |  | |  | | |  | | |  | | |  | |  |
| FMIN FA x10^-2^ | -0.007 | | -0.028 | | | 0.013 | | | 0.482 | | | 0.859 | |  |
| Subgroup IPS impaired (Ref. IPS preserved) | 0.369 | | -1.978 | | | 2.715 | | | 0.755 | | |  | |  |
| Interaction (FMIN FA x10^-2^, Subgroup) | -0.007 | | -0.058 | | | 0.044 | | | 0.785 | | |  | |  |
| Age | 0.076 | | 0.069 | | | 0.084 | | | **< 0.001** | | |  | |  |
| Sex (Ref. Female) | 0.075 | | -0.086 | | | 0.235 | | | 0.356 | | |  | |  |
| Education (years) | -0.026 | | -0.057 | | | 0.004 | | | 0.090 | | |  | |  |
| EDSS | -0.014 | | -0.095 | | | 0.067 | | | 0.732 | | |  | |  |
| **SDMT** |  | |  | | |  | | |  | | |  | |  |
| FMIN FA x10^-2^ | -0.365 | | -1.021 | | | 0.292 | | | 0.271 | | | 0.391 | |  |
| Subgroup IPS impaired (Ref. IPS preserved) | -40.757 | | -115.898 | | | 34.385 | | | 0.283 | | |  | |  |
| Interaction (FMIN FA x10^-2^, Subgroup) | 0.649 | | -0.978 | | | 2.276 | | | 0.429 | | |  | |  |
| Age | -0.056 | | -0.309 | | | 0.198 | | | 0.663 | | |  | |  |
| Sex (Ref. Female) | -4.223 | | -9.363 | | | 0.918 | | | 0.106 | | |  | |  |
| Education (years) | 0.989 | | 0.010 | | | 1.968 | | | **0.048** | | |  | |  |
| EDSS | -4.282 | | -6.884 | | | -1.680 | | | **0.002** | | |  | |  |
| **CTT-1** |  | |  | | |  | | |  | | |  | |  |
| FMIN FA x10^-2^ | 0.155 | | -0.806 | | | 1.116 | | | 0.748 | | | 0.324 | |  |
| Subgroup IPS impaired (Ref. IPS preserved) | 93.551 | | -16.440 | | | 203.543 | | | 0.094 | | |  | |  |
| Interaction (FMIN FA x10^2^, Subgroup) | -1.584 | | -3.966 | | | 0.797 | | | 0.189 | | |  | |  |
| Age | 0.147 | | -0.223 | | | 0.518 | | | 0.430 | | |  | |  |
| Sex (Ref. Female) | 7.967 | | 0.442 | | | 15.492 | | | **0.038** | | |  | |  |
| Education (years) | 0.803 | | -0.630 | | | 2.236 | | | 0.267 | | |  | |  |
| EDSS | 2.281 | | -1.527 | | | 6.090 | | | 0.236 | | |  | |  |
| **CTT-2** |  | |  | | |  | | |  | | |  | |  |
| FMIN FA x10^-2^ | 0.157 | | -1.116 | | | 1.430 | | | 0.806 | | | **0.523** | |  |
| Subgroup IPS impaired (Ref. IPS preserved) | 235.649 | | 89.924 | | | 381.374 | | | **0.002** | | |  | |  |
| Interaction (FMIN FA x10^-2^, Subgroup) | -4.331 | | -7.486 | | | -1.176 | | | **0.008** | | |  | |  |
| Age | 0.329 | | -0.162 | | | 0.820 | | | 0.186 | | |  | |  |
| Sex (Ref. Female) | 10.864 | | 0.895 | | | 20.834 | | | **0.033** | | |  | |  |
| Education (years) | 0.820 | | -1.079 | | | 2.719 | | | 0.392 | | |  | |  |
| EDSS | 7.265 | | 2.219 | | | 12.311 | | | **0.005** | | |  | |  |
| **PASAT z- cognitive** |  | |  | | |  | | |  | | |  | |  |
| L pars opercularis thk x10[mm] | -0.014 | | -0.061 | | | 0.034 | | | 0.569 | | | 0.859 | |  |
| Subgroup IPS impaired (Ref. IPS preserved) | -0.006 | | -2.349 | | | 2.337 | | | 0.996 | | |  | |  |
| Interaction (L pars opercularis thk[mm]x10, Subgroup) | 0.002 | | -0.103 | | | 0.107 | | | 0.973 | | |  | |  |
| Age | 0.077 | | 0.069 | | | 0.085 | | | **< 0.001** | | |  | |  |
| Sex (Ref. Female) | 0.043 | | -0.112 | | | 0.198 | | | 0.580 | | |  | |  |
| Education | -0.022 | | -0.052 | | | 0.008 | | | 0.146 | | |  | |  |
| EDSS | -0.021 | | -0.106 | | | 0.064 | | | 0.619 | | |  | |  |
| **SDMT** |  | |  | | |  | | |  | | |  | |  |
| L pars opercularis thk x10[mm] | 0.926 | | -0.519 | | | 2.372 | | | 0.205 | | | 0.437 | |  |
| Subgroup CI (Ref. IPS preserved) | -64.221 | | -136.103 | | | 7.661 | | | 0.079 | | |  | |  |
| Interaction (L pars opercularis thk x10[mm], Subgroup) | 2.494 | | -0.727 | | | 5.715 | | | 0.127 | | |  | |  |
| Age | -0.038 | | -0.278 | | | 0.203 | | | 0.755 | | |  | |  |
| Sex (Ref. Female) | -4.102 | | -8.856 | | | 0.652 | | | 0.090 | | |  | |  |
| Education | 1.137 | | 0.222 | | | 2.052 | | | 0.016 | | |  | |  |
| EDSS | -3.320 | | -5.932 | | | -0.709 | | | 0.014 | | |  | |  |
| **CTT-1** |  | |  | | |  | | |  | | |  | |  |
| L pars opercularis thk x10[mm] | -1.427 | | -3.619 | | | 0.765 | | | 0.198 | | | 0.330 | |  |
| Subgroup IPS impaired (Ref. IPS preserved) | -51.156 | | -160.153 | | | 57.840 | | | 0.352 | | |  | |  |
| Interaction (L pars opercularis thk x10[mm], Subgroup) | 3.172 | | -1.711 | | | 8.056 | | | 0.199 | | |  | |  |
| Age | 0.158 | | -0.206 | | | 0.522 | | | 0.390 | | |  | |  |
| Sex (Ref. Female) | 6.508 | | -0.701 | | | 13.717 | | | 0.076 | | |  | |  |
| Education | 1.117 | | -0.270 | | | 2.505 | | | 0.113 | | |  | |  |
| EDSS | 1.819 | | -2.141 | | | 5.779 | | | 0.362 | | |  | |  |
| **CTT-2** |  | |  | | |  | | |  | | |  | |  |
| L pars opercularis thk x10[mm] | -1.121 | | -4.089 | | | 1.848 | | | 0.454 | | | 0.506 | |  |
| Subgroup IPS impaired (Ref. IPS preserved) | 156.775 | | 9.184 | | | 304.367 | | | 0.038 | | |  | |  |
| Interaction (L pars opercularis thk x10[mm], Subgroup) | -5.550 | | -12.163 | | | 1.063 | | | 0.099 | | |  | |  |
| Age | 0.381 | | -0.113 | | | 0.874 | | | 0.128 | | |  | |  |
| Sex (Ref. Female) | 7.676 | | -2.086 | | | 17.437 | | | 0.121 | | |  | |  |
| Education | 1.086 | | -0.793 | | | 2.964 | | | 0.253 | | |  | |  |
| EDSS | 5.557 | | 0.195 | | | 10.919 | | | 0.042 | | |  | |  |
| **PASAT z-cognitive** |  | |  | | |  | | |  | | |  | |  |
| L isthmus cingulate thk x10[mm] | 0.027 | | -0.016 | | | 0.070 | | | 0.212 | | | 0.861 | |  |
| Subgroup IPS impaired (Ref. IPS preserved) | 0.712 | | -1.308 | | | 2.731 | | | 0.484 | | |  | |  |
| Interaction (L isthmus cingulate thk x10[mm], Subgroup) | -0.031 | | -0.128 | | | 0.065 | | | 0.519 | | |  | |  |
| Age | 0.078 | | 0.070 | | | 0.086 | | | < 0.001 | | |  | |  |
| Sex (Ref. Female) | 0.054 | | -0.097 | | | 0.205 | | | 0.479 | | |  | |  |
| Education | -0.025 | | -0.054 | | | 0.005 | | | 0.096 | | |  | |  |
| EDSS | 0.001 | | -0.083 | | | 0.084 | | | 0.990 | | |  | |  |
| **SDMT** |  | |  | | |  | | |  | | |  | |  |
| L isthmus cingulate thk x10[mm] | 0.828 | | -0.541 | | | 2.197 | | | 0.231 | | | 0.397 | |  |
| Subgroup IPS impaired (Ref. IPS preserved) | -20.581 | | -85.305 | | | 44.143 | | | 0.528 | | |  | |  |
| Interaction (L isthmus cingulate thk x10[mm], Subgroup) | 0.488 | | -2.620 | | | 3.597 | | | 0.755 | | |  | |  |
| Age | 0.008 | | -0.250 | | | 0.266 | | | 0.951 | | |  | |  |
| Sex (Ref. Female) | -4.459 | | -9.312 | | | 0.394 | | | 0.071 | | |  | |  |
| Education (years) | 1.006 | | 0.068 | | | 1.944 | | | 0.036 | | |  | |  |
| EDSS | -3.727 | | -6.417 | | | -1.037 | | | 0.007 | | |  | |  |
| **CTT-1** |  | |  | | |  | | |  | | |  | |  |
| L isthmus cingulate thk x10[mm] | -0.244 | | -2.285 | | | 1.797 | | | 0.812 | | | 0.306 | |  |
| Subgroup CI (Ref. IPS preserved) | 17.444 | | -79.062 | | | 113.950 | | | 0.719 | | |  | |  |
| Interaction (L isthmus cingulate thk x10[mm], Subgroup) | 0.149 | | -4.486 | | | 4.784 | | | 0.949 | | |  | |  |
| Age | 0.152 | | -0.233 | | | 0.537 | | | 0.434 | | |  | |  |
| Sex (Ref. Female) | 7.310 | | 0.074 | | | 14.547 | | | **0.048** | | |  | |  |
| Education (years) | 0.952 | | -0.447 | | | 2.350 | | | 0.179 | | |  | |  |
| EDSS | 2.045 | | -1.967 | | | 6.056 | | | 0.312 | | |  | |  |
| **CTT-2** |  | |  | | |  | | |  | | |  | |  |
| L isthmus cingulate thk x10[mm] | -2.298 | | -5.025 | | | 0.429 | | | 0.097 | | | 0.502 | |  |
| Subgroup IPS impaired (Ref. IPS preserved) | 77.261 | | -51.693 | | | 206.215 | | | 0.236 | | |  | |  |
| Interaction (L isthmus cingulate thk x10[mm], Subgroup) | -2.008 | | -8.201 | | | 4.185 | | | 0.520 | | |  | |  |
| Age | 0.238 | | -0.277 | | | 0.752 | | | 0.360 | | |  | |  |
| Sex (Ref. Female) | 7.625 | | -2.045 | | | 17.294 | | | 0.120 | | |  | |  |
| Education (years) | 1.435 | | -0.434 | | | 3.304 | | | 0.130 | | |  | |  |
| EDSS | 5.632 | | 0.272 | | | 10.992 | | | **0.040** | | |  | |  |
| **PASAT z-cognitive** |  | |  | | |  | | |  | | |  | |  |
| L insula thk x10[mm] | 0.010 | | -0.038 | | | 0.058 | | | 0.668 | | | 0.858 | |  |
| Subgroup CI (Ref. IPS preserved) | 0.608 | | -2.071 | | | 3.288 | | | 0.652 | | |  | |  |
| Interaction (L insula thk x10[mm] , Subgroup) | -0.021 | | -0.125 | | | 0.082 | | | 0.679 | | |  | |  |
| Age | 0.077 | | 0.069 | | | 0.085 | | | **< 0.001** | | |  | |  |
| Sex (Ref. Female) | 0.058 | | -0.097 | | | 0.213 | | | 0.456 | | |  | |  |
| Education (years) | -0.023 | | -0.053 | | | 0.007 | | | 0.133 | | |  | |  |
| EDSS | -0.008 | | -0.097 | | | 0.081 | | | 0.858 | | |  | |  |
| **SDMT** |  | |  | | |  | | |  | | |  | |  |
| L insula thk x10[mm] | 1.229 | | -0.159 | | | 2.616 | | | 0.082 | | | **0.497** | |  |
| Subgroup IPS impaired (Ref. IPS preserved) | -103.767 | | -181.428 | | | -26.107 | | | **0.010** | | |  | |  |
| Interaction (L insula thk x10[mm],^,^ Subgroup) | 3.580 | | 0.589 | | | 6.571 | | | **0.020** | | |  | |  |
| Age | -0.057 | | -0.286 | | | 0.173 | | | **0.623** | | |  | |  |
| Sex (Ref. Female) | -5.269 | | -9.751 | | | -0.787 | | | **0.022** | | |  | |  |
| Education | 1.372 | | 0.506 | | | 2.238 | | | **0.002** | | |  | |  |
| EDSS | -2.785 | | -5.359 | | | -0.212 | | | **0.034** | | |  | |  |
| **CTT-1** |  | |  | | |  | | |  | | |  | |  |
| L insula thk x10[mm] | -1.112 | | -3.353 | | | 1.128 | | | 0.325 | | | 0.321 | |  |
| Subgroup IPS impaired (Ref. IPS preserved) | 31.024 | | -94.387 | | | 156.434 | | | 0.623 | | |  | |  |
| Interaction (L insula thk x10[mm], Subgroup) | -0.395 | | -5.225 | | | 4.434 | | | 0.871 | | |  | |  |
| Age | 0.155 | | -0.215 | | | 0.525 | | | 0.407 | | |  | |  |
| Sex (Ref. Female) | 7.235 | | -0.003 | | | 14.473 | | | **0.050** | | |  | |  |
| Education | 0.809 | | -0.589 | | | 2.207 | | | 0.252 | | |  | |  |
| EDSS | 1.229 | | -2.927 | | | 5.385 | | | 0.557 | | |  | |  |
| **CTT-2** |  | |  | | |  | | |  | | |  | |  |
| L insula thk x10[mm] | -0.881 | | -3.755 | | | 1.994 | | | 0.543 | | | **0.550** | |  |
| Subgroup CI (Ref. IPS preserved) | 265.470 | | 104.547 | | | 426.393 | | | 0.002 | | |  | |  |
| Interaction (L insula thk x10[mm], Subgroup) | -8.826 | | -15.023 | | | -2.629 | | | **0.006** | | |  | |  |
| Age | 0.443 | | -0.032 | | | 0.918 | | | 0.067 | | |  | |  |
| Sex (Ref. Female) | 10.186 | | 0.899 | | | 19.473 | | | **0.032** | | |  | |  |
| Education | 0.716 | | -1.078 | | | 2.510 | | | 0.428 | | |  | |  |
| EDSS | 5.226 | | -0.107 | | | 10.559 | | | 0.055 | | |  | |  |
| **PASAT z-cognitive** |  | |  | | |  | | |  | | |  | |  |
| L thalamus vol. x10^4^ | 0.001 | | -0.017 | | | 0.019 | | | 0.923 | | | 0.858 | |  |
| Subgroup IPS impaired (Ref. IPS preserved) | 0.069 | | -1.230 | | | 1.367 | | | 0.916 | | |  | |  |
| Interaction (L thalamus vol. x10^4^, Subgroup) | -0.000 | | -0.029 | | | 0.029 | | | 0.983 | | |  | |  |
| Age | 0.077 | | 0.069 | | | 0.085 | | | < 0.001 | | |  | |  |
| Sex (Ref. Female) | 0.054 | | -0.104 | | | 0.211 | | | 0.501 | | |  | |  |
| Education | -0.022 | | -0.052 | | | 0.007 | | | 0.132 | | |  | |  |
| EDSS | -0.014 | | -0.098 | | | 0.070 | | | 0.745 | | |  | |  |
| **SDMT** |  | |  | | |  | | |  | | |  | |  |
| L thalamus vol. x10^-4^ | 0.684 | | 0.179 | | | 1.189 | | | **0.009** | | | **0.507** | |  |
| Subgroup IPS impaired (Ref. IPS preserved) | -21.305 | | -58.469 | | | 15.860 | | | 0.256 | | |  | |  |
| Interaction (L thalamus vol. x10^-4^, Subgroup) | 0.300 | | -0.534 | | | 1.133 | | | 0.476 | | |  | |  |
| Age | -0.025 | | -0.254 | | | 0.204 | | | 0.828 | | |  | |  |
| Sex (Ref. Female) | -3.457 | | -7.973 | | | 1.060 | | | 0.131 | | |  | |  |
| Education | 1.041 | | 0.198 | | | 1.884 | | | **0.016** | | |  | |  |
| EDSS | -3.737 | | -6.139 | | | -1.334 | | | **0.003** | | |  | |  |
| **CTT-1** |  | |  | | |  | | |  | | |  | |  |
| L thalamus vol. x10^4^ | -0.151 | | -0.962 | | | 0.661 | | | 0.712 | | | 0.342 | |  |
| Subgroup IPS impaired (Ref. IPS preserved) | 54.473 | | -5.227 | | | 114.174 | | | 0.073 | | |  | |  |
| Interaction (L thalamus vol. x10^4^, Subgroup) | -0.801 | | -2.141 | | | 0.538 | | | 0.237 | | |  | |  |
| Age | 0.183 | | -0.185 | | | 0.550 | | | 0.325 | | |  | |  |
| Sex (Ref. Female) | 7.247 | | -0.008 | | | 14.501 | | | 0.050 | | |  | |  |
| Education | 0.973 | | -0.381 | | | 2.327 | | | 0.156 | | |  | |  |
| EDSS | 1.499 | | -2.361 | | | 5.359 | | | 0.441 | | |  | |  |
| **CTT-2** |  | |  | | |  | | |  | | |  | |  |
| L thalamus vol. x10^-4^ | -0.922 | | -1.927 | | | 0.083 | | | 0.072 | | | **0.594** | |  |
| Subgroup IPS impaired (Ref. IPS preserved) | 106.583 | | 32.617 | | | 180.549 | | | 0.005 | | |  | |  |
| Interaction (L thalamus vol. x10^-4^, Subgroup) | -1.708 | | -3.368 | | | -0.049 | | | **0.044** | | |  | |  |
| Age | 0.405 | | -0.050 | | | 0.861 | | | 0.080 | | |  | |  |
| Sex (Ref. Female) | 7.085 | | -1.903 | | | 16.074 | | | 0.120 | | |  | |  |
| Education | 1.377 | | -0.301 | | | 3.055 | | | 0.106 | | |  | |  |
| EDSS | 5.343 | | 0.561 | | | 10.125 | | | 0.029 | | |  | |  |
| **PASAT z-cognitive** |  | |  | | |  | | |  | | |  | |  |
| R thalamus vol. x10^3^ | -0.009 | | -0.186 | | | 0.168 | | | 0.917 | | | 0.858 | |  |
| Subgroup IPS impaired (Ref. IPS preserved) | 0.087 | | -1.258 | | | 1.432 | | | 0.898 | | |  | |  |
| Interaction (R thalamus vol. x10^3^, Subgroup) | -0.010 | | -0.343 | | | 0.323 | | | 0.952 | | |  | |  |
| Age | 0.077 | | 0.069 | | | 0.085 | | | **< 0.001** | | |  | |  |
| Sex (Ref. Female) | 0.052 | | -0.106 | | | 0.209 | | | 0.515 | | |  | |  |
| Education | -0.023 | | -0.052 | | | 0.007 | | | 0.130 | | |  | |  |
| EDSS | -0.015 | | -0.099 | | | 0.069 | | | 0.722 | | |  | |  |
| **SDMT** |  | |  | | |  | | |  | | |  | |  |
| R thalamus vol. x10^3^ | 6.109 | | 1.007 | | | 11.211 | | | **0.020** | | | **0.500** | |  |
| Subgroup IPS impaired (Ref. IPS preserved) | -32.713 | | -71.506 | | | 6.080 | | | 0.097 | | |  | |  |
| Interaction (R thalamus vol. x10^3^, Subgroup) | 6.386 | | -3.226 | | | 15.998 | | | 0.189 | | |  | |  |
| Age | -0.056 | | -0.283 | | | 0.172 | | | 0.626 | | |  | |  |
| Sex (Ref. Female) | -4.583 | | -9.119 | | | -0.047 | | | 0.048 | | |  | |  |
| Education | 1.149 | | 0.299 | | | 1.998 | | | 0.009 | | |  | |  |
| EDSS | -3.414 | | -5.834 | | | -0.994 | | | 0.006 | | |  | |  |
| **CTT-1** |  | |  | | |  | | |  | | |  | |  |
| R thalamus vol. x10^3^ | 0.072 | | -8.018 | | | 8.162 | | | 0.986 | | | 0.349 | |  |
| Subgroup IPS impaired (Ref. IPS preserved) | 71.796 | | 10.281 | | | 133.310 | | | 0.023 | | |  | |  |
| Interaction (R thalamus vol. x10^3^, Subgroup) | -13.227 | | -28.468 | | | 2.015 | | | 0.088 | | |  | |  |
| Age | 0.193 | | -0.168 | | | 0.554 | | | 0.289 | | |  | |  |
| Sex (Ref. Female) | 8.333 | | 1.141 | | | 15.526 | | | 0.024 | | |  | |  |
| Education | 0.874 | | -0.473 | | | 2.221 | | | 0.200 | | |  | |  |
| EDSS | 1.269 | | -2.568 | | | 5.106 | | | 0.511 | | |  | |  |
| **CTT-2** |  | |  | | |  | | |  | | |  | |  |
| R thalamus vol. x10^-3^ | -3.045 | | -12.965 | | | 6.875 | | | 0.542 | | | **0.606** | |  |
| Subgroup CI (Ref. IPS preserved) | 166.811 | | 91.381 | | | 242.241 | | | < 0.001 | | |  | |  |
| Interaction (R thalamus vol. x10^-3^, Subgroup) | -34.072 | | -52.761 | | | -15.383 | | | **< 0.001** | | |  | |  |
| Age | 0.464 | | 0.022 | | | 0.907 | | | 0.040 | | |  | |  |
| Sex (Ref. Female) | 10.698 | | 1.878 | | | 19.518 | | | 0.018 | | |  | |  |
| Education | 1.104 | | -0.548 | | | 2.756 | | | 0.187 | | |  | |  |
| EDSS | 4.473 | | -0.233 | | | 9.178 | | | 0.062 | | |  | |  |
| **PASAT z-cognitive** |  | |  | | |  | | |  | | |  | |  |
| WMH vol. x10^-4^ | 0.003 | | -0.001 | | | 0.007 | | | 0.166 | | | 0.862 | |  |
| Subgroup IPS impaired (Ref. IPS preserved) | 0.121 | | -0.139 | | | 0.382 | | | 0.356 | | |  | |  |
| Interaction (WMH vol. x10^4^, Subgroup) | -0.003 | | -0.008 | | | 0.003 | | | 0.329 | | |  | |  |
| Age | 0.076 | | 0.068 | | | 0.084 | | | < 0.001 | | |  | |  |
| Sex (Ref. Female) | 0.049 | | -0.102 | | | 0.200 | | | 0.523 | | |  | |  |
| Education (years) | -0.024 | | -0.053 | | | 0.005 | | | 0.107 | | |  | |  |
| EDSS | -0.020 | | -0.101 | | | 0.061 | | | 0.623 | | |  | |  |
| **SDMT** |  | |  | | |  | | |  | | |  | |  |
| WMH vol. x10^4^ | -0.152 | | -0.279 | | | -0.025 | | | **0.020** | | | **0.456** | |  |
| Subgroup IPS impaired (Ref. IPS preserved) | -10.276 | | -18.240 | | | -2.312 | | | **0.012** | | |  | |  |
| Interaction (WMH vol. x10^4^, Subgroup) | 0.052 | | -0.108 | | | 0.213 | | | 0.518 | | |  | |  |
| Age | 0.018 | | -0.222 | | | 0.258 | | | 0.883 | | |  | |  |
| Sex (Ref. Female) | -4.920 | | -9.534 | | | -0.307 | | | **0.037** | | |  | |  |
| Education (years) | 1.123 | | 0.237 | | | 2.010 | | | **0.014** | | |  | |  |
| EDSS | -3.675 | | -6.156 | | | -1.194 | | | **0.004** | | |  | |  |
| **CTT-1** |  | |  | | |  | | |  | | |  | |  |
| WMH vol. x10^-4^ | 0.096 | | -0.094 | | | 0.285 | | | 0.316 | | | **0.370** | |  |
| Subgroup CI (Ref. IPS preserved) | 14.637 | | 2.720 | | | 26.554 | | | **0.017** | | |  | |  |
| Interaction (WMH vol. x10^4^, Subgroup) | 0.084 | | -0.156 | | | 0.324 | | | 0.489 | | |  | |  |
| Age | 0.111 | | -0.248 | | | 0.470 | | | 0.540 | | |  | |  |
| Sex (Ref. Female) | 7.947 | | 1.043 | | | 14.850 | | | **0.025** | | |  | |  |
| Education (years) | 0.902 | | -0.425 | | | 2.229 | | | 0.179 | | |  | |  |
| EDSS | 1.544 | | -2.168 | | | 5.257 | | | 0.409 | | |  | |  |
| **CTT-2** |  | |  | | |  | | |  | | |  | |  |
| WMH vol. x10^-4^ | 0.243 | | -0.005 | | | 0.491 | | | 0.054 | | | **0.566** | |  |
| Subgroup IPS impaired (Ref. IPS preserved) | 27.008 | | 11.425 | | | 42.592 | | | **< 0.001** | | |  | |  |
| Interaction (WMH vol. x10^4^, Subgroup) | 0.094 | | -0.220 | | | 0.408 | | | 0.552 | | |  | |  |
| Age | 0.270 | | -0.200 | | | 0.739 | | | 0.256 | | |  | |  |
| Sex (Ref. Female) | 9.521 | | 0.493 | | | 18.548 | | | 0.039 | | |  | |  |
| Education (years) | 1.200 | | -0.535 | | | 2.935 | | | 0.172 | | |  | |  |
| EDSS | 5.696 | | 0.841 | | | 10.551 | | | **0.022** | | |  | |  |
| **PASAT z-cognitive** |  |  |  | |  | |  | | | |  |  |  |  |
| CC central vol. x10^5^ | 0.003 | | -0.004 | | | 0.010 | | | | 0.331 | | | 0.860 |  |
| Subgroup CI (Ref. IPS preserved) | 0.124 | | -0.317 | | | 0.565 | | | | 0.577 | | |  |  |
| Interaction (CC central vol. x10^5^, Subgroup) | -0.002 | | -0.015 | | | 0.011 | | | | 0.750 | | |  |  |
| Age | 0.077 | | 0.069 | | | 0.085 | | | | **< 0.001** | | |  |  |
| Sex (Ref. Female) | 0.072 | | -0.085 | | | 0.229 | | | | 0.361 | | |  |  |
| Education (years) | -0.022 | | -0.051 | | | 0.007 | | | | 0.137 | | |  |  |
| EDSS | -0.006 | | -0.090 | | | 0.078 | | | | 0.882 | | |  |  |
| **SDMT** |  |  |  | |  | |  | | | |  |  |  |  |
| CC central vol. x10^5^ | 0.208 | | -0.002 | | | 0.418 | | | | 0.052 | | | **0.469** |  |
| Subgroup IPS impaired (Ref. IPS preserved ) | -18.072 | | -31.285 | | | -4.860 | | | | **0.008** | | |  |  |
| Interaction (CC central vol. x10^5^, Subgroup) | 0.247 | | -0.131 | | | 0.625 | | | | 0.196 | | |  |  |
| Age | -0.084 | | -0.320 | | | 0.152 | | | | 0.478 | | |  |  |
| Sex (Ref. Female) | -3.152 | | -7.850 | | | 1.546 | | | | 0.185 | | |  |  |
| Education (years) | 1.037 | | 0.162 | | | 1.913 | | | | **0.021** | | |  |  |
| EDSS | -3.124 | | -5.636 | | | -0.611 | | | | **0.016** | | |  |  |
| **CTT-1** |  |  |  | |  | |  | | | |  |  |  |  |
| CC central vol. x10^-5^ | -0.173 | | -0.503 | | | 0.158 | | | | 0.301 | | | 0.318 |  |
| Subgroup IPS impaired (Ref. IPS preserved) | 17.204 | | -3.612 | | | 38.021 | | | | 0.104 | | |  |  |
| Interaction (CC central vol. x10^5^, Subgroup) | 0.095 | | -0.500 | | | 0.691 | | | | 0.750 | | |  |  |
| Age | 0.165 | | -0.207 | | | 0.537 | | | | 0.379 | | |  |  |
| Sex (Ref. Female) | 6.313 | | -1.089 | | | 13.715 | | | | 0.093 | | |  |  |
| Education (years) | 0.912 | | -0.468 | | | 2.292 | | | | 0.192 | | |  |  |
| EDSS | 1.790 | | -2.168 | | | 5.747 | | | | 0.370 | | |  |  |
| **CTT-2** |  |  |  | |  | |  | | | |  |  |  |  |
| CC central vol. x10^-5^ | -0.359 | | -0.783 | | | 0.065 | | | | 0.095 | | | 0.549 |  |
| Subgroup IPS impaired (Ref. IPS preserved) | 56.653 | | 29.971 | | | 83.336 | | | | **< 0.001** | | |  |  |
| Interaction (CC central vol. x10^5^, Subgroup) | -0.666 | | -1.430 | | | 0.097 | | | | 0.086 | | |  |  |
| Age | 0.485 | | 0.008 | | | 0.961 | | | | **0.046** | | |  |  |
| Sex (Ref. Female) | 5.534 | | -3.954 | | | 15.021 | | | | 0.248 | | |  |  |
| Education (years) | 1.360 | | -0.409 | | | 3.129 | | | | 0.130 | | |  |  |
| EDSS | 4.711 | | -0.362 | | | 9.784 | | | | 0.068 | | |  |  |
| **PASAT z-cognitive** |  | |  | | |  | | | |  | | |  |  |
| NBV x10^-2^ | 0.009 | | -0.010 | | | 0.029 | | | | 0.328 | | | 0.860 |  |
| Subgroup IPS impaired (Ref. IPS preserved) | 0.822 | | -1.728 | | | 3.373 | | | | 0.522 | | |  |  |
| Interaction (NBV x10^2^, Subgroup) | -0.011 | | -0.046 | | | 0.025 | | | | 0.553 | | |  |  |
| Age | 0.077 | | 0.070 | | | 0.085 | | | | < 0.001 | | |  |  |
| Sex (Ref. Female) | 0.062 | | -0.091 | | | 0.216 | | | | 0.419 | | |  |  |
| Education (years) | -0.023 | | -0.052 | | | 0.006 | | | | 0.121 | | |  |  |
| EDSS | -0.009 | | -0.092 | | | 0.075 | | | | 0.835 | | |  |  |
| **SDMT** |  | |  | | |  | | | |  | | |  |  |
| NBV x10^-2^ | 0.432 | | -0.155 | | | 1.019 | | | | 0.146 | | | 0.441 |  |
| Subgroup IPS impaired (Ref. IPS preserved) | -57.617 | | -135.973 | | | 20.739 | | | | 0.147 | | |  |  |
| Interaction (NBV x10^2^, Subgroup) | 0.665 | | -0.420 | | | 1.750 | | | | 0.225 | | |  |  |
| Age | -0.031 | | -0.271 | | | 0.209 | | | | 0.798 | | |  |  |
| Sex (Ref. Female) | -3.892 | | -8.608 | | | 0.824 | | | | 0.104 | | |  |  |
| Education (years) | 1.121 | | 0.221 | | | 2.021 | | | | 0.015 | | |  |  |
| EDSS | -3.355 | | -5.924 | | | -0.786 | | | | 0.011 | | |  |  |
| **CTT-1** |  | |  | | |  | | | |  | | |  |  |
| NBV x10^2^ | -0.166 | | -1.073 | | | 0.741 | | | | 0.716 | | | 0.309 |  |
| Subgroup IPS impaired (Ref. IPS preserved) | -12.147 | | -133.217 | | | 108.923 | | | | 0.842 | | |  |  |
| Interaction (NBVx10^2^, Subgroup) | 0.454 | | -1.223 | | | 2.130 | | | | 0.591 | | |  |  |
| Age | 0.156 | | -0.215 | | | 0.528 | | | | 0.404 | | |  |  |
| Sex (Ref. Female) | 7.244 | | -0.042 | | | 14.531 | | | | 0.051 | | |  |  |
| Education (years) | 0.961 | | -0.429 | | | 2.352 | | | | 0.172 | | |  |  |
| EDSS | 2.238 | | -1.730 | | | 6.207 | | | | 0.264 | | |  |  |
| **CTT-2** |  | |  | | |  | | | |  | | |  |  |
| NBV x10^2^ | -0.813 | | -1.974 | | | 0.348 | | | | 0.167 | | | 0.544 |  |
| Subgroup IPS impaired (Ref. IPS preserved) | 182.585 | | 27.511 | | | 337.659 | | | | 0.022 | | |  |  |
| Interaction (NBV x10^2^, Subgroup) | -2.061 | | -4.208 | | | 0.086 | | | | 0.060 | | |  |  |
| Age | 0.367 | | -0.108 | | | 0.843 | | | | 0.128 | | |  |  |
| Sex (Ref. Female) | 6.653 | | -2.681 | | | 15.986 | | | | 0.159 | | |  |  |
| Education | 1.115 | | -0.666 | | | 2.896 | | | | 0.216 | | |  |  |
| EDSS | 4.944 | | -0.140 | | | 10.027 | | | | 0.056 | | |  |  |
| **PASAT z-cognitive** |  | |  | | |  | | | |  | | |  |  |
| R ATR AD x10^-3^[mm^2^/s] | 1.049 | | -0.773 | | | 2.870 | | | | 0.254 | | | 0.861 |  |
| Subgroup IPS impaired (Ref. IPS preserved) | 1.668 | | -2.047 | | | 5.384 | | | | 0.373 | | |  |  |
| Interaction (R ATR AD x10^-3^[mm^2^/s], Subgroup) | -1.435 | | -4.715 | | | 1.845 | | | | 0.385 | | |  |  |
| Age | 0.077 | | 0.069 | | | 0.084 | | | | **< 0.001** | | |  |  |
| Sex (Ref. Female) | 0.052 | | -0.100 | | | 0.203 | | | | 0.498 | | |  |  |
| Education (years) | -0.024 | | -0.053 | | | 0.006 | | | | 0.117 | | |  |  |
| EDSS | -0.020 | | -0.102 | | | 0.061 | | | | 0.619 | | |  |  |
| **SDMT** |  | |  | | |  | | | |  | | |  |  |
| R ATR AD x10^-3^[mm^2^/s] | 32.174 | | -25.882 | | | 90.230 | | | | 0.272 | | | 0.402 |  |
| Subgroup IPS impaired (Ref. IPS preserved) | 85.804 | | -32.621 | | | 204.230 | | | | 0.153 | | |  |  |
| Interaction (R ATR AD x10^-3^[mm^2^/s], Subgroup) | -85.361 | | -189.907 | | | 19.185 | | | | 0.108 | | |  |  |
| Age | -0.062 | | -0.310 | | | 0.187 | | | | 0.623 | | |  |  |
| Sex (Ref. Female) | -4.897 | | -9.722 | | | -0.071 | | | | **0.047** | | |  |  |
| Education (years) | 0.937 | | -0.010 | | | 1.884 | | | | 0.052 | | |  |  |
| EDSS | -4.428 | | -7.029 | | | -1.827 | | | | **0.001** | | |  |  |
| **CTT-1** |  | |  | | |  | | | |  | | |  |  |
| R ATR AD x10^-3^[mm^2^/s] | -55.332 | | -140.670 | | | 30.005 | | | | 0.200 | | | 0.331 |  |
| Subgroup IPS impaired (Ref. IPS preserved) | 22.702 | | -151.373 | | | 196.777 | | | | 0.795 | | |  |  |
| Interaction (R ATR AD x10^-3^[mm^2^/s], Subgroup) | -1.206 | | -154.879 | | | 152.467 | | | | 0.988 | | |  |  |
| Age | 0.167 | | -0.198 | | | 0.533 | | | | 0.364 | | |  |  |
| Sex (Ref. Female) | 7.062 | | -0.031 | | | 14.155 | | | | 0.051 | | |  |  |
| Education (years) | 0.825 | | -0.567 | | | 2.217 | | | | 0.241 | | |  |  |
| EDSS | 2.515 | | -1.308 | | | 6.338 | | | | 0.194 | | |  |  |
| **CTT-2** |  | |  | | |  | |  | | | | |  |  |
| R ATR AD x10^-3^[mm^2^/s] | -107.936 | | -224.638 | | | 8.767 | | | | 0.069 | | | 0.497 |  |
| Subgroup IPS impaired (Ref. IPS preserved) | -170.334 | | -408.390 | | | 67.721 | | | | 0.158 | | |  |  |
| Interaction (R ATR AD x10^-3^[mm^2^/s], Subgroup) | 183.259 | | -26.896 | | | 393.414 | | | | 0.086 | | |  |  |
| Age | 0.431 | | -0.069 | | | 0.931 | | | | 0.090 | | |  |  |
| Sex (Ref. Female) | 8.636 | | -1.064 | | | 18.336 | | | | 0.080 | | |  |  |
| Education | 1.479 | | -0.424 | | | 3.383 | | | | 0.125 | | |  |  |
| EDSS | 7.701 | | 2.473 | | | 12.929 | | | | **0.005** | | |  |  |
| **PASAT z-cognitive** |  | |  | | |  | | | |  | | |  |  |
| R ATR FA x10^-1^ | -0.124 | | -0.468 | | | 0.221 | | | | 0.476 | | | 0.859 |  |
| Subgroup IPS impaired (Ref. IPS preserved) | 0.069 | | -2.824 | | | 2.961 | | | | 0.962 | | |  |  |
| Interaction (R ATR FA x10^-1^, Subgroup) | -0.006 | | -0.722 | | | 0.709 | | | | 0.987 | | |  |  |
| Age | 0.077 | | 0.069 | | | 0.085 | | | | **< 0.001** | | |  |  |
| Sex (Ref. Female) | 0.062 | | -0.092 | | | 0.216 | | | | 0.426 | | |  |  |
| Education | -0.023 | | -0.053 | | | 0.006 | | | | 0.121 | | |  |  |
| EDSS | -0.006 | | -0.089 | | | 0.078 | | | | 0.887 | | |  |  |
| **SDMT** |  | |  | | |  | | | |  | | |  |  |
| R ATR FA x10^-1^ | 2.393 | | -8.595 | | | 13.381 | | | | 0.665 | | | 0.395 |  |
| Subgroup IPS impaired (Ref. IPS preserved) | 53.196 | | -38.981 | | | 145.374 | | | | 0.253 | | |  |  |
| Interaction (R ATR FA x10^-1^, Subgroup) | -15.852 | | -38.652 | | | 6.949 | | | | 0.170 | | |  |  |
| Age | -0.055 | | -0.306 | | | 0.195 | | | | 0.660 | | |  |  |
| Sex (Ref. Female) | -4.451 | | -9.365 | | | 0.462 | | | | 0.075 | | |  |  |
| Education | 0.955 | | 0.009 | | | 1.901 | | | | 0.048 | | |  |  |
| EDSS | -4.151 | | -6.811 | | | -1.490 | | | | **0.003** | | |  |  |
| **CTT-1** |  | |  | | |  | | | |  | | |  |  |
| R ATR FA x10^-1^ | -2.683 | | -18.951 | | | 13.586 | | | | 0.743 | | | 0.314 |  |
| Subgroup IPS impaired (Ref. IPS preserved) | 60.001 | | -76.473 | | | 196.474 | | | | 0.383 | | |  |  |
| Interaction (R ATR FA x10^-1^, Subgroup) | -9.838 | | -43.595 | | | 23.919 | | | | 0.563 | | |  |  |
| Age | 0.147 | | -0.224 | | | 0.517 | | | | 0.431 | | |  |  |
| Sex (Ref. Female) | 7.865 | | 0.590 | | | 15.140 | | | | 0.035 | | |  |  |
| Education | 0.838 | | -0.563 | | | 2.239 | | | | 0.236 | | |  |  |
| EDSS | 2.478 | | -1.461 | | | 6.418 | | | | 0.213 | | |  |  |
| **CTT-2** |  | |  | | |  | | | |  | | |  |  |
| R ATR FA x10^-1^ | 1.497 | | -21.152 | | | 24.146 | | | | 0.895 | | | 0.465 |  |
| Subgroup IPS impaired (Ref. IPS preserved) | 30.123 | | -159.878 | | | 220.124 | | | | 0.753 | | |  |  |
| Interaction (R ATR FA x10^-1^, Subgroup) | 1.575 | | -45.422 | | | 48.572 | | | | 0.947 | | |  |  |
| Age | 0.393 | | -0.123 | | | 0.909 | | | | 0.133 | | |  |  |
| Sex (Ref. Female) | 8.329 | | -1.799 | | | 18.458 | | | | 0.105 | | |  |  |
| Education | 1.308 | | -0.643 | | | 3.258 | | | | 0.185 | | |  |  |
| EDSS | 6.892 | | 1.408 | | | 12.377 | | | | 0.015 | | |  |  |
| **PASAT z-cognitive** |  | |  | | |  | | | |  | | |  |  |
| L ATR FA x10^-1^ | 0.012 | | -0.321 | | | 0.345 | | | | 0.944 | | | 0.858 |  |
| Subgroup IPS impaired (Ref. IPS preserved) | 0.359 | | -2.393 | | | 3.111 | | | | 0.795 | | |  |  |
| Interaction (L ATR FA x10^-1^, Subgroup) | -0.074 | | -0.735 | | | 0.587 | | | | 0.824 | | |  |  |
| Age | 0.077 | | 0.069 | | | 0.085 | | | | **< 0.001** | | |  |  |
| Sex (Ref. Female) | 0.054 | | -0.100 | | | 0.207 | | | | 0.487 | | |  |  |
| Education | -0.023 | | -0.052 | | | 0.007 | | | | 0.134 | | |  |  |
| EDSS | -0.013 | | -0.096 | | | 0.071 | | | | 0.764 | | |  |  |
| **SDMT** |  | |  | | |  | | | |  | | |  |  |
| L ATR FA x10^-1^ | 5.652 | | -4.963 | | | 16.267 | | | | 0.292 | | | 0.390 |  |
| Subgroup IPS impaired (Ref. IPS preserved) | 32.971 | | -54.658 | | | 120.600 | | | | 0.455 | | |  |  |
| Interaction (L ATR FA x10^-1^, Subgroup) | -10.531 | | -31.581 | | | 10.519 | | | | 0.321 | | |  |  |
| Age | -0.030 | | -0.284 | | | 0.224 | | | | 0.817 | | |  |  |
| Sex (Ref. Female) | -4.551 | | -9.445 | | | 0.343 | | | | 0.068 | | |  |  |
| Education | 1.109 | | 0.164 | | | 2.055 | | | | **0.022** | | |  |  |
| EDSS | -4.206 | | -6.851 | | | -1.562 | | | | **0.002** | | |  |  |
| **CTT-1** |  | |  | | |  | | | |  | | |  |  |
| L ATR FA x10^-1^ | -2.075 | | -17.500 | | | 13.351 | | | | 0.789 | | | 0.334 |  |
| Subgroup IPS impaired (Ref. IPS preserved) | 103.671 | | -23.670 | | | 231.012 | | | | 0.109 | | |  |  |
| Interaction (L ATR FA x10^-1^, Subgroup) | -19.955 | | -50.545 | | | 10.634 | | | | 0.197 | | |  |  |
| Age | 0.128 | | -0.241 | | | 0.497 | | | | 0.492 | | |  |  |
| Sex (Ref. Female) | 8.024 | | 0.913 | | | 15.136 | | | | **0.028** | | |  |  |
| Education | 0.846 | | -0.528 | | | 2.220 | | | | 0.223 | | |  |  |
| EDSS | 2.740 | | -1.104 | | | 6.583 | | | | 0.159 | | |  |  |
| **CTT-2** |  | |  | | |  | | | |  | | |  |  |
| L ATR FA x10^-1^ | -6.715 | | -28.448 | | | 15.019 | | | | 0.539 | | | 0.468 |  |
| Subgroup CI (Ref. IPS preserved) | 20.184 | | -159.230 | | | 199.598 | | | | 0.823 | | |  |  |
| Interaction (L ATR FA x10^-1^, Subgroup) | 3.926 | | -39.173 | | | 47.024 | | | | 0.856 | | |  |  |
| Age | 0.361 | | -0.159 | | | 0.881 | | | | 0.170 | | |  |  |
| Sex (Ref. Female) | 8.542 | | -1.478 | | | 18.562 | | | | 0.093 | | |  |  |
| Education (years) | 1.204 | | -0.732 | | | 3.140 | | | | 0.219 | | |  |  |
| EDSS | 7.200 | | 1.786 | | | 12.615 | | | | **0.010** | | |  |  |
| **PASAT z-cognitive** |  | |  | | |  | | | |  | | |  |  |
| L ATR AD x10^-3^[mm^2^/s] | 1.116 | | -0.419 | | | 2.651 | | | | 0.151 | | | 0.863 |  |
| Subgroup IPS impaired (Ref. IPS preserved) | 1.898 | | -1.653 | | | 5.448 | | | | 0.290 | | |  |  |
| Interaction (L ATR AD x10^-3^[mm^2^/s], Subgroup) | -1.653 | | -4.809 | | | 1.504 | | | | 0.300 | | |  |  |
| Age | 0.077 | | 0.069 | | | 0.085 | | | | **< 0.001** | | |  |  |
| Sex (Ref. Female) | 0.060 | | -0.092 | | | 0.212 | | | | 0.431 | | |  |  |
| Education (years) | -0.023 | | -0.053 | | | 0.007 | | | | 0.128 | | |  |  |
| EDSS | -0.017 | | -0.098 | | | 0.064 | | | | 0.672 | | |  |  |
| **SDMT** |  | |  | | |  | | | |  | | |  |  |
| L ATR AD x10^-3^[mm^2^/s] | 13.326 | | -35.950 | | | 62.602 | | | | 0.591 | | | 0.401 |  |
| Subgroup IPS impaired (Ref. CP) | 80.908 | | -33.088 | | | 194.905 | | | | 0.161 | | |  |  |
| Interaction (L ATR AD x10^-3^[mm^2^/s], Subgroup) | -81.474 | | -182.811 | | | 19.862 | | | | 0.113 | | |  |  |
| Age | -0.069 | | -0.321 | | | 0.183 | | | | 0.586 | | |  |  |
| Sex (Ref. Female) | -5.021 | | -9.901 | | | -0.141 | | | | **0.044** | | |  |  |
| Education (years) | 0.878 | | -0.086 | | | 1.842 | | | | 0.073 | | |  |  |
| EDSS | -4.114 | | -6.708 | | | -1.521 | | | | **0.002** | | |  |  |
| **CTT-1** |  | |  | | |  | | | |  | | |  |  |
| L ATR AD x10^-3^[mm^2^/s] | -38.435 | | -110.792 | | | 33.921 | | | | 0.293 | | | 0.332 |  |
| Subgroup IPS impaired (Ref. IPS preserved) | 68.885 | | -98.506 | | | 236.276 | | | | 0.414 | | |  |  |
| Interaction (L ATR AD x10^-3^[mm^2^/s], Subgroup) | -42.316 | | -191.117 | | | 106.485 | | | | 0.572 | | |  |  |
| Age | 0.110 | | -0.261 | | | 0.480 | | | | 0.556 | | |  |  |
| Sex (Ref. Female) | 6.438 | | -0.727 | | | 13.604 | | | | 0.077 | | |  |  |
| Education (years) | 0.675 | | -0.741 | | | 2.090 | | | | 0.345 | | |  |  |
| EDSS | 2.526 | | -1.283 | | | 6.334 | | | | 0.190 | | |  |  |
| **CTT-2** |  | |  | | |  | | | |  | | |  |  |
| L ATR AD x10^-3^[mm^2^/s] | -74.956 | | -175.290 | | | 25.379 | | | | 0.141 | | | 0.483 |  |
| Subgroup IPS impaired (Ref. IPS preserved) | -69.722 | | -301.838 | | | 162.394 | | | | 0.551 | | |  |  |
| Interaction (L ATR AD x10^-3^[mm^2^/s], Subgroup) | 95.148 | | -111.190 | | | 301.486 | | | | 0.360 | | |  |  |
| Age | 0.366 | | -0.147 | | | 0.880 | | | | 0.159 | | |  |  |
| Sex (Ref. Female) | 7.824 | | -2.112 | | | 17.760 | | | | 0.121 | | |  |  |
| Education (years) | 1.287 | | -0.676 | | | 3.250 | | | | 0.195 | | |  |  |
| EDSS | 7.276 | | 1.994 | | | 12.557 | | | | **0.008** | | |  |  |
| **PASAT z-cognitive** |  | |  | | |  | | | |  | | |  |  |
| L CCG AD x10^-3^[mm^2^/s] | -0.491 | | -1.412 | | | 0.430 | | | | 0.291 | | | 0.860 |  |
| Subgroup IPS impaired (Ref. IPS preserved) | -0.677 | | -2.836 | | | 1.482 | | | | 0.533 | | |  |  |
| Interaction (L CCG AD x10^-3^[mm^2^/s], Subgroup) | 0.606 | | -1.146 | | | 2.357 | | | | 0.492 | | |  |  |
| Age | 0.077 | | 0.069 | | | 0.085 | | | | **< 0.001** | | |  |  |
| Sex (Ref. Female) | 0.061 | | -0.091 | | | 0.214 | | | | 0.425 | | |  |  |
| Education (years) | -0.023 | | -0.052 | | | 0.006 | | | | 0.124 | | |  |  |
| EDSS | -0.020 | | -0.102 | | | 0.062 | | | | 0.628 | | |  |  |
| **SDMT** |  | |  | | |  | | | |  | | |  |  |
| L CCG AD x10^-3^[mm^2^/s] | 3.185 | | -26.699 | | | 33.069 | | | | 0.832 | | | 0.378 |  |
| Subgroup IPS impaired (Ref. IPS preserved) | -0.682 | | -70.736 | | | 69.372 | | | | 0.985 | | |  |  |
| Interaction (L CCG AD x10^-3^[mm^2^/s], Subgroup) | -8.198 | | -65.033 | | | 48.637 | | | | 0.774 | | |  |  |
| Age | -0.040 | | -0.294 | | | 0.213 | | | | 0.753 | | |  |  |
| Sex (Ref. Female) | -4.813 | | -9.757 | | | 0.131 | | | | 0.056 | | |  |  |
| Education (years) | 1.062 | | 0.116 | | | 2.009 | | | | **0.028** | | |  |  |
| EDSS | -4.162 | | -6.816 | | | -1.508 | | | | **0.003** | | |  |  |
| **CTT-1** |  | |  | | |  | | | |  | | |  |  |
| L CCG AD x10^-3^[mm^2^/s] | 6.258 | | -37.446 | | | 49.963 | | | | 0.776 | | | 0.311 |  |
| Subgroup IPS impaired (Ref. IPS preserved) | 56.855 | | -45.597 | | | 159.307 | | | | 0.272 | | |  |  |
| Interaction (L CCG AD x10^-3^[mm^2^/s], Subgroup) | -29.428 | | -112.548 | | | 53.692 | | | | 0.482 | | |  |  |
| Age | 0.175 | | -0.196 | | | 0.546 | | | | 0.348 | | |  |  |
| Sex (Ref. Female) | 7.169 | | -0.061 | | | 14.398 | | | | 0.052 | | |  |  |
| Education (years) | 0.931 | | -0.454 | | | 2.315 | | | | 0.184 | | |  |  |
| EDSS | 2.321 | | -1.561 | | | 6.203 | | | | 0.237 | | |  |  |
| **CTT-2** |  | |  | | |  | | | |  | | |  |  |
| L CCG AD x10^-3^[mm^2^/s] | -25.778 | | -86.023 | | | 34.466 | | | | 0.396 | | | 0.474 |  |
| Subgroup IPS impaired (Ref. IPS preserved) | -30.812 | | -172.036 | | | 110.411 | | | | 0.664 | | |  |  |
| Interaction (L CCG AD x10^-3^[mm^2^/s], Subgroup) | 55.066 | | -59.509 | | | 169.642 | | | | 0.341 | | |  |  |
| Age | 0.369 | | -0.143 | | | 0.880 | | | | 0.155 | | |  |  |
| Sex (Ref. Female) | 9.062 | | -0.904 | | | 19.028 | | | | 0.074 | | |  |  |
| Education (years) | 1.278 | | -0.630 | | | 3.186 | | | | 0.186 | | |  |  |
| EDSS | 6.615 | | 1.264 | | | 11.966 | | | | 0.016 | | |  |  |
| **PASAT z-cognitive** |  | |  | | |  | | | |  | | |  |  |
| L CCG FA x10^-1^ | -0.133 | | -0.326 | | | 0.061 | | | | 0.176 | | | 0.862 |  |
| Subgroup IPS impaired (Ref. IPS preserved) | -0.434 | | -2.192 | | | 1.324 | | | | 0.624 | | |  |  |
| Interaction (L CCG FA x10^-1^, Subgroup) | 0.100 | | -0.267 | | | 0.468 | | | | 0.587 | | |  |  |
| Age | 0.076 | | 0.068 | | | 0.084 | | | | **< 0.001** | | |  |  |
| Sex (Ref. Female) | 0.065 | | -0.090 | | | 0.219 | | | | 0.407 | | |  |  |
| Education (years) | -0.024 | | -0.053 | | | 0.005 | | | | 0.109 | | |  |  |
| EDSS | -0.013 | | -0.093 | | | 0.068 | | | | 0.755 | | |  |  |
| **SDMT** |  | |  | | |  | | | |  | | |  |  |
| L CCG FA x10^-1^ | 0.307 | | -5.781 | | | 6.394 | | | | 0.920 | | | **0.423** |  |
| Subgroup IPS impaired (Ref. IPS preserved) | -63.093 | | -118.352 | | | -7.834 | | | | **0.026** | | |  |  |
| Interaction (L CCG FA x10^-1^, Subgroup) | 11.046 | | -0.502 | | | 22.595 | | | | 0.061 | | |  |  |
| Age | -0.037 | | -0.286 | | | 0.212 | | | | 0.766 | | |  |  |
| Sex (Ref. Female) | -5.902 | | -10.759 | | | -1.046 | | | | **0.018** | | |  |  |
| Education (years) | 1.053 | | 0.140 | | | 1.966 | | | | **0.024** | | |  |  |
| EDSS | -4.230 | | -6.760 | | | -1.700 | | | | **0.001** | | |  |  |
| **CTT-1** |  | |  | | |  | | | |  | | |  |  |
| L CCG FA x10^-1^ | 6.377 | | -2.760 | | | 15.514 | | | | 0.168 | | | 0.327 |  |
| Subgroup CI (Ref. IPS preserved) | 61.234 | | -21.712 | | | 144.180 | | | | 0.145 | | |  |  |
| Interaction (L CCG FA x10^-1^, Subgroup) | -8.469 | | -25.804 | | | 8.866 | | | | 0.333 | | |  |  |
| Age | 0.217 | | -0.157 | | | 0.590 | | | | 0.251 | | |  |  |
| Sex (Ref. Female) | 7.099 | | -0.190 | | | 14.389 | | | | 0.056 | | |  |  |
| Education (years) | 0.992 | | -0.379 | | | 2.362 | | | | 0.153 | | |  |  |
| EDSS | 2.128 | | -1.669 | | | 5.926 | | | | 0.267 | | |  |  |
| **CTT-2** |  | |  | | |  | | | |  | | |  |  |
| L CCG FA x10^-1^ | 1.283 | | -11.458 | | | 14.025 | | | | 0.841 | | | 0.474 |  |
| Subgroup IPS impaired (Ref. IPS preserved) | 92.713 | | -22.957 | | | 208.383 | | | | 0.114 | | |  |  |
| Interaction (L CCG FA x10^-1^, Subgroup) | -11.859 | | -36.033 | | | 12.315 | | | | 0.331 | | |  |  |
| Age | 0.396 | | -0.124 | | | 0.917 | | | | 0.133 | | |  |  |
| Sex (Ref. Female) | 9.467 | | -0.698 | | | 19.633 | | | | 0.067 | | |  |  |
| Education (years) | 1.307 | | -0.604 | | | 3.219 | | | | 0.177 | | |  |  |
| EDSS | 7.008 | | 1.712 | | | 12.304 | | | | **0.010** | | |  |  |
| **PASAT z-cognitive** |  | |  | | |  | | | |  | | |  |  |
| R CCG AD x10^-3^[mm^2^/s] | -0.119 | | -1.132 | | | 0.895 | | 0.816 | | | | | | 0.858 |
| Subgroup IPS impaired (Ref. IPS preserved) | -0.216 | | -3.317 | | | 2.886 | | | | 0.890 | | |  |  |
| Interaction (R CCG AD x10^-3^[mm^2^/s], Subgroup) | 0.232 | | -2.459 | | | 2.924 | | | | 0.864 | | |  |  |
| Age | 0.077 | | 0.069 | | | 0.085 | | | | **< 0.001** | | |  |  |
| Sex (Ref. Female) | 0.055 | | -0.101 | | | 0.212 | | | | 0.483 | | |  |  |
| Education (years) | -0.023 | | -0.053 | | | 0.007 | | | | 0.127 | | |  |  |
| EDSS | -0.013 | | -0.095 | | | 0.068 | | | | 0.743 | | |  |  |
| **SDMT** |  | |  | | |  | | | |  | | |  |  |
| R CCG AD x10^-3^[mm^2^/s] | 3.090 | | -29.112 | | | 35.292 | | | | 0.849 | | | 0.393 |  |
| Subgroup IPS impaired (Ref. IPS preserved) | -67.598 | | -166.142 | | | 30.946 | | | | 0.175 | | |  |  |
| Interaction (R CCG AD x10^-3^[mm^2^/s], Subgroup) | 49.493 | | -36.034 | | | 135.019 | | | | 0.252 | | |  |  |
| Age | -0.055 | | -0.305 | | | 0.195 | | | | 0.662 | | |  |  |
| Sex (Ref. Female) | -5.018 | | -9.989 | | | -0.046 | | | | **0.048** | | |  |  |
| Education (years) | 1.061 | | 0.116 | | | 2.006 | | | | **0.028** | | |  |  |
| EDSS | -4.309 | | -6.908 | | | -1.710 | | | | **0.002** | | |  |  |
| **CTT-1** |  | |  | | |  | | | |  | | |  |  |
| R CCG AD x10^-3^[mm^2^/s] | 18.010 | | -28.721 | | | 64.742 | | | | 0.444 | | | 0.339 |  |
| Subgroup IPS impaired (Ref. IPS preserved) | 149.244 | | 6.237 | | | 292.251 | | | | **0.041** | | |  |  |
| Interaction (R CCG AD x10^-3^[mm^2^/s], Subgroup) | -111.805 | | -235.920 | | | 12.311 | | | | 0.077 | | |  |  |
| Age | 0.184 | | -0.179 | | | 0.547 | | | | 0.315 | | |  |  |
| Sex (Ref. Female) | 7.069 | | -0.146 | | | 14.283 | | | | 0.055 | | |  |  |
| Education (years) | 1.036 | | -0.335 | | | 2.407 | | | | 0.136 | | |  |  |
| EDSS | 2.258 | | -1.513 | | | 6.030 | | | | 0.236 | | |  |  |
| **CTT-2** |  | |  | | |  | | | |  | | |  |  |
| R CCG AD x10^-3^[mm^2^/s] | -7.102 | | -73.116 | | | 58.912 | | | | 0.831 | | | 0.469 |  |
| Subgroup IPS impaired (Ref. IPS preserved) | -38.471 | | -240.487 | | | 163.545 | | | | 0.705 | | |  |  |
| Interaction (R CCG AD x10^-3^[mm^2^/s], Subgroup) | 65.056 | | -110.274 | | | 240.386 | | | | 0.461 | | |  |  |
| Age | 0.376 | | -0.137 | | | 0.888 | | | | 0.148 | | |  |  |
| Sex (Ref. Female) | 8.538 | | -1.653 | | | 18.730 | | | | 0.099 | | |  |  |
| Education (years) | 1.239 | | -0.698 | | | 3.176 | | | | 0.206 | | |  |  |
| EDSS | 6.943 | | 1.615 | | | 12.271 | | | | **0.011** | | |  |  |
| **PASAT z-cognitive** |  | |  | | |  | | | |  | | |  |  |
| R CCG FA x10^-1^ | -0.082 | | -0.243 | | | 0.079 | | | | 0.311 | | | 0.860 |  |
| Subgroup IPS impaired (Ref. IPS preserved) | -0.243 | | -1.504 | | | 1.019 | | | | 0.702 | | |  |  |
| Interaction (R CCG FA x10^1^, Subgroup) | 0.064 | | -0.221 | | | 0.348 | | | | 0.655 | | |  |  |
| Age | 0.076 | | 0.069 | | | 0.084 | | | | **< 0.001** | | |  |  |
| Sex (Ref. Female) | 0.069 | | -0.086 | | | 0.224 | | | | 0.380 | | |  |  |
| Education | -0.025 | | -0.054 | | | 0.005 | | | | 0.099 | | |  |  |
| EDSS | -0.009 | | -0.091 | | | 0.073 | | | | 0.825 | | |  |  |
| **SDMT** |  | |  | | |  | | | |  | | |  |  |
| R CCG FA x10^-1^ | 1.330 | | -3.732 | | | 6.392 | | | | 0.601 | | | 0.413 |  |
| Subgroup IPS impaired (Ref. IPS preserved) | -36.200 | | -75.942 | | | 3.542 | | | | 0.073 | | |  |  |
| Interaction (R CCG FA x10^-1^, Subgroup) | 5.962 | | -2.999 | | | 14.923 | | | | 0.189 | | |  |  |
| Age | -0.018 | | -0.266 | | | 0.230 | | | | 0.886 | | |  |  |
| Sex (Ref. Female) | -5.254 | | -10.136 | | | -0.372 | | | | **0.035** | | |  |  |
| Education (years) | 1.108 | | 0.179 | | | 2.037 | | | | **0.020** | | |  |  |
| EDSS | -4.440 | | -7.011 | | | -1.869 | | | | **< 0.001** | | |  |  |
| **CTT-1** |  | |  | | |  | | | |  | | |  |  |
| R CCG FA x10^-1^ | 5.002 | | -2.512 | | | 12.517 | | | | 0.188 | | | 0.330 |  |
| Subgroup IPS impaired (Ref. IPS preserved) | 24.027 | | -34.975 | | | 83.030 | | | | 0.419 | | |  |  |
| Interaction (R CCG FA x10^-1^, Subgroup) | -0.533 | | -13.836 | | | 12.771 | | | | 0.937 | | |  |  |
| Age | 0.207 | | -0.162 | | | 0.575 | | | | 0.267 | | |  |  |
| Sex (Ref. Female) | 6.210 | | -1.038 | | | 13.458 | | | | 0.092 | | |  |  |
| Education (years) | 1.072 | | -0.308 | | | 2.451 | | | | 0.126 | | |  |  |
| EDSS | 1.821 | | -1.996 | | | 5.638 | | | | 0.344 | | |  |  |
| **CTT-2** |  | |  | | |  | | | |  | | |  |  |
| R CCG FA x10^-1^ | 2.633 | | -7.908 | | | 13.174 | | | | 0.620 | | | 0.470 |  |
| Subgroup IPS impaired (Ref. IPS preserved) | 27.596 | | -55.164 | | | 110.356 | | | | 0.508 | | |  |  |
| Interaction (R CCG FA x10^-1^, Subgroup) | 2.164 | | -16.497 | | | 20.824 | | | | 0.818 | | |  |  |
| Age | 0.417 | | -0.100 | | | 0.934 | | | | 0.112 | | |  |  |
| Sex (Ref. Female) | 7.813 | | -2.354 | | | 17.979 | | | | 0.130 | | |  |  |
| Education (years) | 1.362 | | -0.572 | | | 3.297 | | | | 0.164 | | |  |  |
| EDSS | 6.767 | | 1.413 | | | 12.120 | | | | 0.014 | | |  |  |
| **PASAT z-cognitive** |  | |  | | |  | | | |  | | |  |  |
| L CST AD x10^-3^[mm^2^/s] | 0.002 | | -2.042 | | | 2.046 | | | | 0.999 | | | 0.858 |  |
| Subgroup IPS impaired (Ref. IPS preserved) | 0.297 | | -3.972 | | | 4.565 | | | | 0.890 | | |  |  |
| Interaction (L CST AD x10^-3^[mm^2^/s], Subgroup) | -0.209 | | -3.857 | | | 3.440 | | | | 0.909 | | |  |  |
| Age | 0.077 | | 0.069 | | | 0.085 | | | | **< 0.001** | | |  |  |
| Sex (Ref. Female) | 0.051 | | -0.104 | | | 0.205 | | | | 0.515 | | |  |  |
| Education | -0.023 | | -0.053 | | | 0.007 | | | | 0.133 | | |  |  |
| EDSS | -0.013 | | -0.096 | | | 0.069 | | | | 0.748 | | |  |  |
| **SDMT** |  | |  | | |  | | | |  | | |  |  |
| L CST AD x10^-3^[mm^2^/s] | -14.882 | | -79.909 | | | 50.146 | | | | 0.649 | | | 0.391 |  |
| Subgroup IPS impaired (Ref. IPS preserved) | 36.709 | | -99.090 | | | 172.509 | | | | 0.591 | | |  |  |
| Interaction (L CST AD x10^-3^[mm^2^/s], Subgroup) | -40.411 | | -156.497 | | | 75.675 | | | | 0.489 | | |  |  |
| Age | -0.041 | | -0.295 | | | 0.212 | | | | 0.747 | | |  |  |
| Sex (Ref. Female) | -4.897 | | -9.813 | | | 0.018 | | | | 0.051 | | |  |  |
| Education | 0.960 | | 0.009 | | | 1.910 | | | | 0.048 | | |  |  |
| EDSS | -4.194 | | -6.823 | | | -1.565 | | | | **0.002** | | |  |  |
| **CTT-1** |  | |  | | |  | | | |  | | |  |  |
| L CST AD x10^-3^[mm^2^/s] | 11.753 | | -83.170 | | | 106.675 | | | | 0.805 | | | 0.329 |  |
| Subgroup IPS impaired (Ref. IPS preserved) | 155.161 | | -43.069 | | | 353.392 | | | | 0.123 | | |  |  |
| Interaction (L CST AD x10^-3^[mm^2^/s], Subgroup) | -114.976 | | -284.430 | | | 54.478 | | | | 0.180 | | |  |  |
| Age | 0.143 | | -0.227 | | | 0.512 | | | | 0.444 | | |  |  |
| Sex (Ref. Female) | 6.707 | | -0.469 | | | 13.882 | | | | 0.066 | | |  |  |
| Education | 0.799 | | -0.589 | | | 2.187 | | | | 0.255 | | |  |  |
| EDSS | 2.436 | | -1.401 | | | 6.273 | | | | 0.209 | | |  |  |
| **CTT-2** |  | |  | | |  | | | |  | | |  |  |
| L CST AD x10^-3^[mm^2^/s] | -48.173 | | -181.219 | | | 84.872 | | | | 0.472 | | | 0.470 |  |
| Subgroup IPS impaired (Ref. IPS preserved) | -49.913 | | -327.755 | | | 227.930 | | | | 0.721 | | |  |  |
| Interaction (L CST AD x10^-3^[mm^2^/s], Subgroup) | 73.953 | | -163.556 | | | 311.462 | | | | 0.536 | | |  |  |
| Age | 0.422 | | -0.096 | | | 0.941 | | | | 0.109 | | |  |  |
| Sex (Ref. Female) | 9.057 | | -1.000 | | | 19.114 | | | | 0.077 | | |  |  |
| Education | 1.254 | | -0.692 | | | 3.199 | | | | 0.203 | | |  |  |
| EDSS | 6.692 | | 1.314 | | | 12.070 | | | | **0.016** | | |  |  |
| **PASAT z-cognitive** |  | |  | | |  | | | |  | | |  |  |
| R CST FA x10^-1^ | -0.352 | | -0.690 | | | -0.015 | | | | **0.041** | | | 0.867 |  |
| Subgroup IPS impaired (Ref. IPS preserved) | -1.632 | | -4.655 | | | 1.391 | | | | 0.285 | | |  |  |
| Interaction (R CST FA x10^-1^, Subgroup) | 0.361 | | -0.279 | | | 1.001 | | | | 0.264 | | |  |  |
| Age | 0.077 | | 0.069 | | | 0.085 | | | | **< 0.001** | | |  |  |
| Sex (Ref. Female) | 0.035 | | -0.114 | | | 0.184 | | | | 0.641 | | |  |  |
| Education | -0.024 | | -0.053 | | | 0.004 | | | | 0.095 | | |  |  |
| EDSS | -0.017 | | -0.097 | | | 0.063 | | | | 0.674 | | |  |  |
| **SDMT** |  | |  | | |  | | | |  | | |  |  |
| R CST FA x10^-1^ | 6.733 | | -4.071 | | | 17.537 | | | | 0.218 | | | **0.422** |  |
| Subgroup IPS impaired (Ref. IPS preserved) | 98.257 | | 1.466 | | | 195.048 | | | | **0.047** | | |  |  |
| Interaction (R CST FA x10^-1^, Subgroup) | -23.105 | | -43.593 | | | -2.616 | | | | **0.028** | | |  |  |
| Age | -0.065 | | -0.310 | | | 0.179 | | | | 0.596 | | |  |  |
| Sex (Ref. Female) | -4.275 | | -9.038 | | | 0.487 | | | | 0.078 | | |  |  |
| Education | 1.170 | | 0.253 | | | 2.087 | | | | **0.013** | | |  |  |
| EDSS | -3.790 | | -6.352 | | | -1.227 | | | | **0.004** | | |  |  |
| **CTT-1** |  | |  | | |  | | | |  | | |  |  |
| R CST FA x10^-1^ | -4.505 | | -20.798 | | | 11.789 | | | | 0.583 | | | **0.320** |  |
| Subgroup IPS impaired (Ref. IPS preserved) | 61.553 | | -84.416 | | | 207.521 | | | | 0.403 | | |  |  |
| Interaction (R CST FA x10^-1^, Subgroup) | -8.571 | | -39.470 | | | 22.328 | | | | 0.581 | | |  |  |
| Age | 0.142 | | -0.228 | | | 0.511 | | | | 0.447 | | |  |  |
| Sex (Ref. Female) | 7.251 | | 0.069 | | | 14.433 | | | | **0.048** | | |  |  |
| Education | 0.970 | | -0.413 | | | 2.353 | | | | 0.166 | | |  |  |
| EDSS | 2.435 | | -1.430 | | | 6.299 | | | | 0.213 | | |  |  |
| **CTT-2** |  | |  | | |  | | | |  | | |  |  |
| R CST FA x10^-1^ | 4.241 | | -17.941 | | | 26.423 | | | | 0.704 | | | 0.493 |  |
| Subgroup IPS impaired (Ref. IPS preserved) | -99.352 | | -298.071 | | | 99.367 | | | | 0.322 | | |  |  |
| Interaction (R CST FA x10^-1^, Subgroup) | 28.575 | | -13.490 | | | 70.640 | | | | 0.180 | | |  |  |
| Age | 0.440 | | -0.063 | | | 0.943 | | | | 0.085 | | |  |  |
| Sex (Ref. Female) | 8.411 | | -1.367 | | | 18.189 | | | | 0.091 | | |  |  |
| Education | 1.156 | | -0.727 | | | 3.039 | | | | 0.225 | | |  |  |
| EDSS | 6.305 | | 1.044 | | | 11.566 | | | | **0.020** | | |  |  |
| **PASAT z-cognitive** |  | |  | | |  | | | |  | | |  |  |
| R CST AD x10^-3^[mm^2^/s] | -0.607 | | -3.036 | | | 1.822 | | | | 0.619 | | | 0.858 |  |
| Subgroup IPS impaired (Ref. IPS preserved) | -0.451 | | -4.564 | | | 3.661 | | | | 0.827 | | |  |  |
| Interaction (R CST AD x10^-3^[mm^2^/s], Subgroup) | 0.440 | | -3.103 | | | 3.983 | | | | 0.805 | | |  |  |
| Age | 0.077 | | 0.069 | | | 0.085 | | | | **< 0.001** | | |  |  |
| Sex (Ref. Female) | 0.049 | | -0.103 | | | 0.202 | | | | 0.520 | | |  |  |
| Education (years) | -0.023 | | -0.053 | | | 0.006 | | | | 0.119 | | |  |  |
| EDSS | -0.014 | | -0.096 | | | 0.068 | | | | 0.731 | | |  |  |
| **SDMT** |  | |  | | |  | | | |  | | |  |  |
| R CST AD x10^-3^[mm^2^/s] | 0.674 | | -76.367 | | | 77.716 | | | | 0.986 | | | 0.398 |  |
| Subgroup IPS impaired (Ref. IPS preserved) | 61.738 | | -68.691 | | | 192.167 | | | | 0.348 | | |  |  |
| Interaction (R CST AD x10^-3^[mm^2^/s], Subgroup) | -62.065 | | -174.423 | | | 50.294 | | | | 0.274 | | |  |  |
| Age | -0.063 | | -0.312 | | | 0.187 | | | | 0.619 | | |  |  |
| Sex (Ref. Female) | -5.008 | | -9.857 | | | -0.159 | | | | **0.043** | | |  |  |
| Education (years) | 1.014 | | 0.075 | | | 1.953 | | | | **0.035** | | |  |  |
| EDSS | -4.037 | | -6.635 | | | -1.440 | | | | **0.003** | | |  |  |
| **CTT-1** |  | |  | | |  | | | |  | | |  |  |
| R CST AD x10^-3^[mm^2^/s] | -40.823 | | -154.398 | | | 72.752 | | | | 0.475 | | | 0.322 |  |
| Subgroup IPS impaired (Ref. IPS preserved) | 47.368 | | -144.911 | | | 239.647 | | | | 0.624 | | |  |  |
| Interaction (R CST AD x10^-3^[mm^2^/s], Subgroup) | -22.347 | | -187.987 | | | 143.292 | | | | 0.788 | | |  |  |
| Age | 0.140 | | -0.228 | | | 0.509 | | | | 0.450 | | |  |  |
| Sex (Ref. Female) | 6.970 | | -0.178 | | | 14.118 | | | | 0.056 | | |  |  |
| Education (years) | 0.828 | | -0.556 | | | 2.212 | | | | 0.236 | | |  |  |
| EDSS | 2.302 | | -1.527 | | | 6.132 | | | | 0.234 | | |  |  |
| **CTT-2** |  | |  | | |  | | | |  | | |  |  |
| R CST AD x10^-3^[mm^2^/s] | -44.866 | | -199.848 | | | 110.117 | | | | 0.565 | | | 0.492 |  |
| Subgroup IPS impaired (Ref. IPS preserved) | -188.085 | | -450.467 | | | 74.297 | | | | 0.157 | | |  |  |
| Interaction (R CST AD x10^-3^[mm^2^/s], Subgroup) | 192.878 | | -33.151 | | | 418.907 | | | | 0.093 | | |  |  |
| Age | 0.431 | | -0.071 | | | 0.934 | | | | 0.091 | | |  |  |
| Sex (Ref. Female) | 9.036 | | -0.718 | | | 18.791 | | | | 0.069 | | |  |  |
| Education (years) | 1.345 | | -0.544 | | | 3.234 | | | | 0.160 | | |  |  |
| EDSS | 6.518 | | 1.293 | | | 11.743 | | | | **0.015** | | |  |  |
| \| **Abbreviations**: AD, axial diffusivity; ATR, anterior thalamic radiation; CAB, cingulum–angular (infracallosal) bundle; CC, corpus callosum; CCG, cingulum–cingulate gyrus (supracallosal) bundle; CI, confidence interval; CST, corticospinal tract; CTT, Color Trails Test; e-TIV, estimated total intracranial volume; FA, fractional anisotropy; ILF, inferior longitudinal fasciculus; IPS, information processing speed; L, left hemisphere; mm, millimetres; NBV, normalized brain volume; R, right hemisphere; R^2^, coefficient of determination; s, second; SDMT, Symbol Digit Modalities Test; SLFP, superior longitudinal fasciculus–parietal bundle; SLFT, superior longitudinal fasciculus–temporal bundle; thk, thickness; UNC, uncinate fasciculus; vol, volume normalized to estimated total intracranial volume; WMH, white matter hypointensities. \| \| --- \| | | | | | | | | | | | | | |  |
